# Supplementary material for: Common elements of service delivery models that optimise quality of life and health service use among older people with advanced progressive conditions: a tertiary systematic review
Source: BMJ Open. 2021 Nov 30;11(12):e048417. doi: 10.1136/bmjopen-2020-048417 (PMC8638152; doi:10.1136/bmjopen-2020-048417)
Supplement: Supplementary data [file bmjopen-2020-048417supp001.pdf]

Supplementary material 1. Eligibility criteria for WHO 'Rapid review of service delivery models for older people at the end of life to maximise quality of life.'

	Inclusion		Exclusion
A	Participants at the end of life or living with advanced disease	Where information is available patients described as being in the last 1-2 years of life, or with advanced disease defined as advanced or metastatic cancer; chronic respiratory disease GOLD stage III-IV / grade C-D; heart failure New York Heart Association stage III or IV; progressive neurological disease; and frailty (excluding pre-frail)	Participants not described as being at the end of life or do not have advanced disease
B	Participants are older people	Where information is available at least 50% of the population must be greater than 60 years old or mean age greater than 60 years old	Where the information is available less than 50% of participants are older than 60 years old or mean age greater than 60 years old
C	Intervention must be a service delivery model aiming to improve quality of life	Service model must be an overarching model of health care provision with multiple components and interacting elements	Intervention is a single component intervention or focussing on post death intervention.
D	Outcome must be focussed on quality of life, function and dignity or cost-effectiveness	Outcomes of quality of life, function and dignity to include wellbeing, resilience, personal satisfaction, empowerment, goal attainment, autonomy, independence, mastery, adaptation, symptoms including pain, breathlessness, anxiety, depression, constipation, falls, any measure of psychosocial or spiritual distress, patient and caregiver satisfaction<br>Outcome of cost effectiveness	Outcome not focussed on quality of life, function or dignity
E	Design must be a review	Review must have searched at least 2 sources, one of which must be an electronic database	Non-review level paper e.g., primary intervention
F	Review may include controlled or non-controlled trials	Review can include trials that are randomised (cluster, parallel, single-stage or cross-over design), non-randomised trials, controlled before-after studies, interrupted time series studies and repeated measures studies. Control group can include usual care, attention control, active control or no control	Review focussing on opinion piece, case studies, case series or descriptive studies

Supplementary material 2. Search Strategy for Medline

The search strategy was adapted for searches on The Cochrane Database of Systematic Reviews, CINAHL and Embase databases [14] and included studies published between January 2000 and October 2017.

	Population EoL /advanced disease	Intervention e.g. hospital	Outcome
MESH terms	Exp Terminally ill /<br>Exp Terminal care/<br>Palliative Care/<br>Frailty/	Exp Patient admission/<br>Exp Patient readmission/<br>Geriatric nursing/<br>Primary nursing/<br>Hospice and palliative care nursing/<br>Exp Nursing services/<br>Symptom Assessment/<br>Geriatric Assessment/<br>Needs assessment/<br>Hospital volunteers/<br>Nursing process/<br>Exp Patient care planning/<br>Exp Progressive patient care/<br>Exp Caregivers/<br>Exp Home care services/<br>Exp Hospice care/<br>Exp Patient Care Team<br>Exp Continuity of Patient Care/	Exp Quality of life/<br>Exp Pain/<br>Exp Pain management/<br>Exp Dyspnea/<br>Exp Anxiety/<br>Exp Anxiety disorders/<br>Depression/<br>Exp Depressive disorder/<br>Personal satisfaction/<br>Exp Activities of daily living/<br>Constipation/<br>Accidental Falls/<br>Exp Mental health/<br>Exp Social isolation/<br>Exp Social support/<br>Exp Patient satisfaction/<br>Exp Budgets/<br>Exp Costs and cost analysis/<br>Economics/<br>Exp Economics, hospital/<br>Exp Economics, medical/<br>Economics, nursing/<br>Exp Fees and charges/<br>Exp Resource allocation/<br>Value of life/
Key terms	EoL.tw<br>End?of?life.tw<br>Dying.tw<br>Palliative.tw<br>Last adj4 life.tw<br>Hospice.tw<br>Life limit*tw<br>Advanced disease*tw<br>Palliative treatment.tw<br>Palliative medicine.tw<br>Terminal care.tw<br>Terminally ill.tw<br>End-of-life care.tw<br>Hospice care.tw<br>Palliation.tw.<br>Palliative care$.tw.<br>Multi*morbidity.tw<br>Co*morbidity.tw<br>((Frail old*) AND (people OR adult* OR person*)).ti,ab<br>Frail*.tw<br>Frail elder*.ti,ab<br>Frailty syndrome*.ti,ab<br>Advanced illness.tw	Integrated care.tw<br>Model adj4 care.tw<br>Multi?disciplin*tw<br>Multi?disciplinary team.tw<br>Volunteer* tw<br>Volunt*tw<br>Hospital adj3 home.tw<br>Comprehensive assess*tw<br>Holistic assess*<br>(special$ adj2 palliat$).tw.<br>Nurse-led.tw<br>Co?ordination adj3 care.tw<br>Care plans.tw<br>Care?giver*.tw<br>Person?centr*.tw<br>Self?manage*.tw<br>Community health worker*.tw<br>Service delivery.tw<br>Community?based.tw<br>Home visit*.tw<br>Case management.tw<br>Care management.tw	Good death.tw<br>Symptom*.tw<br>Concern*.tw<br>Attainment<br>Dignity.tw<br>Empowerment.tw<br>Transition*.tw<br>Pain.tw<br>Dyspn?ea.tw<br>Breathless*.tw<br>Anxiety.tw<br>Anxious.tw<br>Depress*.tw<br>Quality of life.tw<br>Qol.tw<br>(quality adj2 life).tw.<br>Distress.tw<br>Wellbeing.tw<br>ADL*.tw<br>Activities of daily living.tw<br>Constipat*.tw<br>Fall*.tw<br>Mobil*.tw<br>Symptom management.tw.<br>Psychosocial.tw.<br>(psycho adj social).tw.<br>Psychological distress.tw.<br>Enablement.tw<br>Mastery.tw<br>Resilience.tw<br>Stress.tw<br>Financ*tw

			(Cost* or economic*).ti<br>(Cost* adj2 (effective* or utilit* or benefit* or minimi*)).ab.<br>Economic model*.tw<br>(Budget* or fee* or financ* or pricing or price* or resource* allocat* or (value adj2 (monetary or money))).ti,ab
BOLEAN TERMS	OR	OR	OR
	AND		
LIMIT	((Overview*.ti OR Review.ti OR Synthesis.ti OR Summary.ti OR Cochrane.ti OR Analysis.ti) AND (reviews.ti OR meta-analyses.ti OR articles.ti OR umbrella.ti)) OR ''umbrella review''.ti,ab OR (meta-review.ti.ab ORMetareview.ti,ab) OR ((overview*.ti OR Reviews.ti) AND (systematic.ti OR Cochrane.ti)) OR (reviews.ti,ab and (meta.ti,ab OR Published.ti,ab OR Quality.ti,ab OR Included.ti,ab OR summar*.ti,ab)) OR (''cochrane reviews''.ti,ab) OR (evidence.ti AND (reviews.ti OR meta-analyses.ti))		

Supplementary material 3. Data extraction framework: CATWOE elements

Service Delivery Model area (CATWOE)	Model elements / processes	Operational definition
**C(customers):** Target population and case mix	Population needs assessment	Population targeted by the intervention
	Setting	Where intervention is delivered: *Hospital in-patients/ hospital out-patients/ home/ primary care/community / mixed settings*
**A(actors):** Workforce including professions, level of skill and training	Multi-disciplinary team care	Multi-disciplinery team comprises ≥3 disciplines
	Rehabilitation expertise or training	Recognised rehabilitation expertise or training (i.e. Allied Health Professionals)
	End of life expertise or training	Recognised Palliative Care expertise or training (i.e. Palliative Care physician/or specialist Palliative Clinical Nurse Specialist or explicit statement of palliative and end of life care training)
	Professional education	Persons delivering intervention are educated and trained to nationally recognised standards and regulations.
**T(transformation process):** Service model elements / components	Comprehensive Assessment	I.e. comprehensive assessment- across multiple domains including physical/psychological/social/spiritual
	Case Management	Each person's overall care assigned to a team or individual
	Collaborative Working	Working across disciplines to plan services and deliver care to meet needs
	Route(s) of access, source and criteria for referral	How are participants recruited or eligible to participate?
	Professional psychosocial support	Explicit psychological support offered as component of intervention (i.e. psychologist/counsellor/Social Worker)
	Contact established with primary care or attending physician	Does interventionist contact physician as part of intervention?
	Patient and family education	Education for patient &/or family caregiver
	Individual multi-disciplinary care plan	Explicit description of multi-disciplinary team care plan
	Medical intervention	Medical intervention part of intervention, not alongside
	Team case rounds	Intervention includes team meetings, not usual care meetings
	Practical support	Any practical help i.e. in home, with medication boxes, equipment

	Early rehabilitation assessment	Intervention includes rehabilitation early in course of persons integrated geriatric care or integrated palliative care
	Systematic risk screening	Risk screening part of intervention delivery
	Discharge planning	Discharge planning a component of intervention
	Bereavement support	As stated
	Spiritual support	As stated
	Advance care planning	Formal advanced care planning
	Emergency response plan	Emergency only or plan for acute changes, i.e. worsening symptoms
	Self-management	As stated
	Medication review	Review part of intervention
	Complexity/medication management	Ongoing management of medication during intervention
**T:** Mode of delivery	Physician home visits	As part of intervention
	Physician available around the clock	As stated
	Interaction between professional and patient	Face to face/telephone/online or combination
	Access to dedicated inpatient beds	As stated
	Around the clock home visits available	As stated
	Ongoing assessment	Intervention includes multiple points of or ongoing assessment
**T:** Operational tools & guidance to support practice, e.g. assessment or decision support tools	Chart in the home	Diary, manual, medical/nursing record
	Medical review: standardized admission assessment	Explicitly reports standardised assessment is used
	Patient-centred care: standardized comprehensive assessment	Evidence of use comprehensive assessment tools or guidance relating to patient needs
**W (worldview):** Methods of integrated working	Joint provision across health and social care	Care involves explicit links between health and social care (in residential/nursing home care or home) providers
	Linkage with hospital	Intervention involves links with hospital services or is provided by hospital
	Linkage between community services	Intervention involves links with community services
	Expert consultation with other providers	Intervention involves consultation with other multi-disciplinary teams.
	Linkage with residential hospice	As stated
	One contact number	As stated- but reports contact number given

Bayly J, *et al. BMJ Open* 2022; 11:e048417. doi: 10.1136/bmjopen-2020-048417

	Ongoing / continuous care	Ongoing care following the intervention made explicit
**W**: Conceptual model	Patient directed goal driven care	Patient involved in setting goals
	Centrality of patient* needs	Intervention focuses on individual patients needs
	Care mandate -service driven or needs- and benefits-driven	Service driven intervention = same intervention delivered to everyone with customisation and tailoring<br>Needs driven = patients' needs determine delivery of individualised intervention components
	Joint decision-making	Patient involved in decision making during delivery of intervention
	Active patient participation	Involves client or patient actively participating in behaviours
	Patient engagement	Intervention targets patient
	Caregiver engagement	Intervention targets caregiver
**W:** Provider Sector(s)	Visiting volunteer sectors	Volunteers explicitly involved in delivery of intervention
**O (Owners)**	Location	Country name
	World Bank status	High, Upper middle, Low Middle, Low
	Health service funding	State, private for profit, private non-profit, voluntary sector, other
**E (environmental constraints):** Country setting, sites, human resources,	Enabling environment	Policy, infrastructure, workforce training, rural or urban settings
	Resource requirements -human resources	Human resources- name all professionals involved in intervention delivery

*for consistency, we decided to use term 'patient' while acknowledging that in some settings the term client may be interchangeable or preferred.

Supplementary material 4. Mapping CATWOE domains to Logic Model template domains

CATWOE Domains	→	Logic Model Domains
**C**<br>**(Customers)**		**Population**<br>Target population and case mix
**A**<br>**(Actors)**		**Service delivery**<br>Workforce including professions, level of skill and training
**T**<br>**(Transformation processes)**		**Service delivery**<br>Mode of delivery<br>**Service components**<br>Service model elements/components<br>Operational tools to support practice
**W**<br>**(Worldview)**		**Service components**<br>Methods of integrated working<br>**Approach to service delivery**<br>Conceptual model
**E**<br>**(Environmental constraints)**		**Context**<br>Setting, sites, size of population served, infrastructure<br>**Implementation**<br>Policy, workforce, training<br>resource requirements

Supplementary material 5. Included study characteristics

Author / year	WHO Region	Country	WBC Income status	Population	Setting	Sample Size	QoL Outcome Measure	HSU
**Integrated Geriatric Care**								
Applegate 1990[1]	Americas	USA	High	Acutely ill older people	Hospital in-pts	156	Basic self-care activities	n/a
Asplund 2000[2]	Europe	Sweden	High	Acutely ill older people	Hospital in-pts	190	n/a	Shorter hospital length of stay and reduced hospital readmissions
Austin 2005[3]	Europe	UK	High	People with heart failure	Hospital out-pts	200	Functional performance (6MWT), perceived exertion (Borg RPE), Minnesota living with heart failure	n/a
Barnes 2012[4]	Americas	USA	High	Acutely ill older people	Hospital in-pts	1632	n/a	Shorter hospital length of stay and reduced hospital readmissions
Blue 2001[5]	Europe	UK	High	People with heart failure	Home	165	n/a	All cause and Heart Failure hospital readmission rates
Burton 2013[6]	W. Pacific	Australia	High	Older people	Home	80	Physical activity tests (i.e. sit to stand), Late life Disability Instrument, Late life function Instrument.	n/a
Capomollo 2002[7]	Europe	Italy	High	People with heart failure	Hospital out-pts	234	n/a	All cause hospital readmission rates
Chang 2005 [8]	Americas	USA	High	People with heart failure	Hospital out-pts	95	Minnosota Living with Heart Failure (MLwHF) and peace subscale of the spiritual quality of life.	n/a
Clark 2013[9]	Americas	USA	High	People with advanced cancer	Home	129	Functional Assessment of Cancer Therapy-General (FACT-G) scale	n/a
Clemson 2004[10]	W. Pacific	Australia	High	Older people	Community	310	Number of falls; falls & mobility efficacy scales; Physical Activity Scale for the	n/a

							Elderly; worry scale; SF36 (physical components and mental components)	
Clemson 2012[11]	W. Pacific	Australia	High	Older people	Home	317	Number of falls, balance and strength, EQ-5D, EQ-VAS, National Health and Nutrition Examination Survey (I), Late life function index, PASE.	n/a
Cline 1998[12]	Europe	Sweden	High	People with heart failure	Mixed secttings (IP, OP)	190	The quality of life in heart failure questionnaire; Nottingham health profile, patient global health assessment	All cause hospital readmission rate
Close 1999[13]	Europe	UK	High	Acutely ill older people	Mixed settings (ER, H)	397	Number of falls, Barthel Index	n/a
Collard 1985[14]	Americas	USA	High	Acutely ill older people	Hospital in-pts	720	Fewer Falls	n/a
Counsell 2007[15]	Americas	USA	High	Older people	Home	951	SR36; Assets & Health Dynamics of the oldest old (AHEAD) survey	Shorter hospital length of stay and reduced hospital readmissions
Covinsky 1997[16]	Americas	USA	High	Acutely ill older people	Hospital in-pts	650	n/a	Shorter hospital length of stay and reduced hospital readmissions
de Lusignan 2001[17]	Europe	UK	High	People with heart failure	Mixed settings (OP, H)	20	General Health Questionnaire and Chronic heart failure symptomology questionnaire	n/a
Doughty 2002[18]	W. Pacific	New Zealand	High	People with heart failure	Mixed settings (IP, H, OP)	197	Minnesota Living with Heart Failure Questionnaire	Hospital Readmission rate for Heart Failure,
Dunbar 2015[19]	Americas	USA	High	People with heart failure	Mixed settings (IP, H, OP)	134	Minnesota Living with Heart Failure Questionnaire, EQ-5D, 6 minute walk test	n/a
Ekman 1998[20]	Europe	Sweden	High	People with heart failure	Hospital out-pts	158	n/a	All cause hospital readmission rate
Fretwell 1990[21]	Americas	USA	High	Acutely ill older people	Mixed settings (IP, OP)	436	n/a	Hospital length of stay

Gary 2010[22]	Americas	USA	High	People with heart failure	Home	74	Minnesota Living with Heart Failure Questionnaire	n/a
Gitlin 2006[23]	Americas	USA	High	Older people	Home	319	Falls Efficacy Scale; three items from Activities-specific Balance Confidence Scale (confident walking up/down stairs, bending picking up slipper from floor., getting in/out of car without falling).	n/a
Goldberg 2003[24]	Americas	USA	High	People with heart failure	Home	282	Medical Outcome Study 12 Item Short Form (SF-12), Medical Outcomes Study Health Distress Scale, Minnesota Living with Heart Failure Questionnaire, and overall Patient Satisfaction (single item) with heart failure care.	n/a
Harrison 2002[25]	Americas	Canada	High	People with heart failure	Mixed settings (IP, H)	192	Minnesota living with Heart Failure Questionnaire, SF 36	Hospital Readmission rate
Jaarsma 1999[26]	Europe	Netherlands	High	People with heart failure	Mixed settings (IP, H)	179	Heart failure Self-Care Behaviour	Hospital Readmission rate
Jerant 2001[27]	Americas	USA	High	People with heart failure	Home	37	n/a	All cause and Heart Failure hospital readmission rate
Kasper 2002[28]	Americas	USA	High	People with heart failure	Mixed settings (IP, OP)	200	Minnesota living with heart failure, Duke activity status index	Readmissions
Krumholz 2002[29]	Americas	USA	High	People with heart failure	Mixed settings	88	n/a	Hospital Readmission rate
Lang 2018[30]	Europe	UK	High	People with heart failure	Home	50	Minnesota Living with Heart Failure Questionnaire; Hospital anxiety and depression scale; EQ-5D	n/a
Laramee 2003[31]	Americas	USA	High	People with heart failure	Mixed settings (IP, H)	287	n/a	Hospital Readmission rate for Heart Failure
Ledwidge 2003[32]	Europe	Ireland	High	People with heart failure	Mixed settings (IP, H)	98	n/a	Heart Failure hospital readmission rates

Luskin 2002[33]	Americas	USA	High	People with heart failure	Hospital out-pts	33	Geriatric Depression Scale, Perceived Stress Scale, Life Orientation Test, State Trait Anxiety Inventory, Medical Outcome Survey Questions 3 and 9, Minnesota Living with Heart Failure, Self-report physical fitness, Six-minute walk,	n/a
Markle-Reid 2010[34]	Americas	Canada	High	Older people	Home	109	Mean number of falls during 6-month Follow-Up	n/a
McVey 1989[35]	Americas	USA	High	Acutely ill older people	Hospital in-pts	178	Measurements of Activities of Daily Living	n/a
Naylor 1994[36]	Americas	USA	High	Acutely ill older people	Mixed settings (IP, H)	276	n/a	Hospital Readmission rate
Naylor 1999[37]	Americas	USA	High	Acutely ill older people	Mixed settings (IP, OP, H)	363	n/a	All cause hospital readmission rate
Northouse 2007[38]	Americas	USA	High	People with cancer	Home	263	Functional Assessment of Cancer Therapy-General (FACT-G) scale	n/a
Pugh 2001[39]	Americas	USA	High	People with heart failure	Mixed settings	58	n/a	Hospital Readmission rate for Heart Failure
Rainville 1999[40]	Americas	USA	High	People with heart failure	Mixed settings (OP, H)	34	n/a	Heart Failure hospital readmission rate
Rich 1995[41]	Americas	USA	High	People with heart failure	Mixed settings (IP, OP)	282	Chronic Heart Failure Questionnaire	All cause hospital readmission rate
Rich 1993[42]	Americas	USA	High	People with heart failure	Mixed setting (IP, OP)	98	Chronic Heart Failure Questionnaire	Readmission during follow-up
Riegel 2002[43]	Americas	USA	High	People with heart failure	Home	358	n/a	All cause and Heart Failure hospital readmission rate
Rubenstein 1984[44]	Americas	USA	High	Acutely ill older people	Hospital in-pts	123	Personal Self-maintenance scale;	n/a
Rubin 1993[45]	Americas	USA	High	Acutely ill older people	Community	200	Katz Activities of daily living Index, Instrumental Activities of Daily Living (Five-Item OARS Scale)	n/a

Saltvedt 2006[46]	Europe	Norway	High	Acutely ill older people	Hospital in-pts	254	Barthel Index	n/a
Serxner 1998[47]	Americas	USA	High	People with heart failure	Hospital out-pts	109	n/a	Hospital Readmission rate
Sherwood 2017[48]	Americas	USA	High	People with heart failure	Community	180	Global score Kansas City Cardiomyopathy Questionnaire (KCCQ); Beck Depression Inventory II; Speilberger State-Trait Anxiety Inventory; Heart Failure Attitudes about Impairment Questionnaire, 6-minute walking test.	Reducing worsening heart failure hospitalisations
Stewart S 1998[49]	W. Pacific	Australia	High	People with heart failure	Mixed settings (IP, H)	97	n/a	All cause and Heart Failure hospital readmission rate
Stewart M 1999 [50]	Americas	USA	High	Acutely ill older people	Hospital in-pts	61	n/a	All cause and Heart Failure hospital readmission rate
Stromberg 2003[51]	Europe	Sweden	High	People with heart failure	Hospital out-pts	106	n/a	Heart Failure hospital readmission rate
Thomas 1993[52]	Americas	USA	High	Acutely ill older people	Hospital in-pts	120	Katz Functional activity rating scale ADL	Hospital length of stay
Trochu 2004[53]	Europe	France	High	People with heart failure	Mixed settings (OP, H)	202	n/a	All cause and Heart Failure hospital readmission rate
Tsuyuki 2004[54]	Americas	Canada	High	People with heart failure	Mixed settings (IP, OP, H)	276	n/a	Hospital Readmission rate for Heart Failure
Varma 1999[55]	Europe	UK	High	People with heart failure	Mixed settings (OP, H)	83	Minnesota living with Heart Failure Questionnaire and the SF-36	n/a
Vidan 2009[56]	Europe	Spain	High	Acutely ill older people	Hospital in-pts	542	Independence in 6 basic Activities of daily living, bathing, dressing, toileting, transferring from bed to chair, continence, and eating.	Length of hospital stay
Wang 2016[57]	SE Asia	Taiwan	High	People with heart failure	Hospital out-pts	92	Piper fatigue Scale (PFS) , Minnesota living with HF	n/a

							questionnaire (MLHFQ) symptom distress, anxiety	
Yu 2010[58]	SE Asia	Hong Kong, China	High	People with heart failure	Hospital out-pts	158	World Health Organization Quality of Life Questionnaire (WHOQOL-BREF-HK)	n/a
Zelada 2009[59]	Americas	Peru	High middle	Acutely ill older people	Hospital in-pts	143	Katz Scale	Length of hospital stay
**Integrated Palliative Care**								
Bakitas 2009[60]	Americas	USA	High	People with advanced cancer	Home	322	Functional Assessment of Chronic Illness Therapy for Palliative Care; Edmonton Symptom Assessment Scale	n/a
Bakitas 2015[61]	Americas	USA	High	People with advanced cancer	Mixed settings (OP, H)	207	Functional Assessment of Chronic Illness Therapy for Palliative Care (FACIT-PAL); FACIT-PAL Treatment Outcome Index; Quality at End of Life (Qual-E), Center for Epidemiological Studies-Depression Scale (CES-D)	n/a
Brannstrom 2014[62]	Europe	Sweden	High	People with heart failure	Mixed settings (OP, H)	72	Edmonton Symptom Assessment System (ESAS), EQ-5D, Kansas City Cardiomyopathy Questionnaire (KCCQ)	n/a
Edmonds 2010[63]	Europe	UK	High	People with multiple sclerosis	Mixed settings (OP, H)	52	Mulitple Sclerosis Impact Scale (MSIS), Palliative Outcome Scale, Modified Lawton Positivity Questionnaire	n/a
Given 2002[64]	Americas	USA	High	People with cancer	Home	113	SF-36	n/a
Higginson 2014[65]	Europe	UK	High	People with advanced diseases	Mixed settings (OP, H)	105	Chronic Respiratory Disease Querstionnaire (mastery), Breathlessness severity, London Chest Actiities of Daily Living Questionnaire, EQ-5D & EQ-VAS, Palliative	n/a

							outcome scale, Hospital anxiety and depression scale.	
Jordhoy 2001[66]	Europe	Norway	High	People with advanced cancer	Mixed settings (IP, OP, H)	434	European Organisation for Research and Treatment of Cancer Quality of Life Questionnaire C30 (EORTC QLQ-C30)	n/a
Lowther 2015[67]	Africa	Kenya	Low middle	People with HIV	Hospital out-pts	120	African Palliative Care Outcome Scale	n/a
Maltoni 2016[68]	Europe	Italy	High	People with advanced cancer	Hospital out-pts	207	Functional Assessment of Cancer Therapy-Hepato-biliary (FACT-HEP) and FACT-HEP Trial Outcome Index.	n/a
Ozcelik 2014[69]	Europe	Turkey	High middle	People with advanced cancer	Mixed settings (IP, OP, H)	44	Edmonton Symptom Assessment Scale (ESAS) and European Organisation for Research and Treatment of Cancer Quality of Life Questionnaire C30 (EORTC QLQ C30)	n/a
Rogers 2017[70]	Americas	USA	High	People with heart failure	Mixed settings (IP, OP, H)	150	Kansas City Cardiomyopathy questionnaire (KCCQ), Functional assessment of chronic illness therapy palliative care scale (FACIT-Pal) assessed at 6 months. Hospital Anxiety and Depression Scale (HADS), Functional Assessment of Chronic Illness Therapy—Spiritual Well-Being [FACIT-Sp)	n/a
Rummans 2006[71]	Americas	USA	High	People with advanced cancer	Hospital out-pts	115	Spitzer QoL Uniscale and Linear analog scales of assessment	n/a
Sidebottom 2015[72]	Americas	USA	High	People with heart failure	Hospital in-pts	232	Minnesota living with Heart Failure Questionnaire	n/a
Steel 2016[73]	Americas	USA	High	People with advanced cancer	Mixed settings (OP, H)	261	Center for epidemiological studies Depression scale	n/a

							(CES-D), Brief pain inventory, FACT-Hepatobiliary.	
Tattersall 2014[74]	W. Pacific	Australia	High	People with advanced cancer	Hospital out-pts	120	McGill QoL questionnaire, Rotterdam Symptom Checklist	n/a
Temel 2010[75]	Americas	USA	High	People with advanced cancer	Hospital out-pts	151	Functional Assessment of Cancer Therapy - Lung; Hospital Anxiety and Depression Scale; Patient Health Questionnaire	n/a
Temel 2017[76]	Americas	US	High	People with advanced cancer	Hospital out-pts	350	Functional Assessment of Cancer Therapy-General (FACT-G) scale; Patient Health Questionnaire-9 (PHQ-9); Hospital Anxiety and Depression Scale(HADS)	n/a
Wong 2016[77]	SE Asia	China	High	People with heart failure	Home	84	MQOL-HK, McGill Quality of Life Questionnaire–Hong Kong adaptation	n/a
Zimmermann 2014[78]	Americas	Canada	High	People with advanced cancer	Mixed settings (OP, H)	461	Functional Assessment of Chronic Illness Therapy Spiritual Well-Being [FACIT-Sp); Quality of Life at the End of Life (Qual E); Edmonton Symptom Assessment System (ESAS); satisfaction with care (FAMCARE); Cancer Rehabilitation Evaluation System Medical Interaction Subscale CARES-MIS	n/a

Key: IP =In-patients; OP = out-patients, ER =Emergency Room, H= home; WBC= World Bank Classification n/a = not assessed or not assessed in meta-analysis

Supplementary material 6. Assessment of Methodological Quality in Included Reviews (AMSTAR)

First Author, Year	A priori design provided	Duplicate study selection/ data extraction	Systematic literature search performed	Status of publication used as an inclusion criterion	List of studies (included and excluded) provided	Characteristics of the included studies provided	Scientific quality of included studies assessed and documented	Scientific quality of included studies used appropriately in formulating conclusions?	Were the methods used to combine the findings of the studies appropriate?	Was the likelihood of publication bias assessed?	Was the conflict of interest included?	Total
**Cui 2019**	No	Yes	Yes	Yes	No	No	Yes	No	Yes	Yes	Yes	**7**
**De Coninck, 2017**	Yes	No	Yes	No	Yes	Yes	Yes	Yes	Yes	No	No	**7**
**Ekdahl 2015**	No	Yes	Yes	No	No	Yes	Yes	Yes	Yes	No	No	**6**
**Fox 2012**	Yes	Yes	Yes	No	Yes	Yes	Yes	Yes	Yes	Yes	Yes	**9**
**Fulton 2019**	Yes	Yes	Yes	No	Yes	Yes	Yes	Yes	Yes	No	Yes	**9**
**Haun 2017**	Yes	Yes	Yes	Yes	Yes	Yes	Yes	Yes	Yes	Yes	No	**9**
**Kavalieratos 2016**	Yes	Yes	Yes	No	No	Yes	Yes	Yes	Yes	Yes	No	**8**
**McAlister 2004**	Yes	Yes	Yes	No	No	No	No	No	Yes	No	No	**4**
**Phillips 2004**	Yes	Yes	Yes	No	No	Yes	Yes	Yes	Yes	Yes	No	**8**
**Kassianos 2018**	Yes	Yes	Yes	Yes	No	Yes	Yes	Yes	Yes	Yes	Yes	**10**
											**Median**	**8**

Supplementary material 7. Risk of Bias Table for included studies

Author/ Year	Randomisation Sequence generation	Allocation Concealment	Blinding of participants and personnel	Blinding of outcome assessments	Incomplete outcome assessment	Selective reporting	Other bias
**Integrated Geriatric Care**							
Applegate 1990	Low	High	High	High	Low	Low	Low
Asplund 2000	Low	Low	High	High	High	Unclear	Low
Austin 2005	Low	Low	Low	Unclear	Low	Low	Unclear
Barnes 2012	Low	High	High	High	Unclear	Low	Low
Blue 2001	Unclear	Unclear	High	Unclear	Unclear	High	Unclear
Burton 2013	Low	Low	High	High	Low	Low	Unclear
Capamello 2002	High	High	High	Unclear	Unclear	High	High
Chang 2005	Low	Unclear	Unclear	Unclear	Low	Low	Unclear
Clark M 2013	High	High	High	High	High	Unclear	Unclear
Clemson 2004	High	Low	High	Low	High	Low	Unclear
Clemson 2012	Low	Low	High	Low	Low	Low	Unclear
Cline 1998	Low	Low	High	Unclear	Low	Low	Unclear
Close 1999	Low	Low	High	High	Low	High	Low
Collard 1985	Low	High	Low	Unclear	High	High	Low
Counsell 2007	Low	Low	Low	Low	Unclear	Unclear	Low
Covinsky 1997	Low	Low	Unclear	Unclear	Low	Low	Low
de Lusigan 2001	Low	Unclear	High	Unclear	Low	Low	Unclear
Doughty 2002	Low	Low	High	Unclear	Low	Low	Unclear
Dunbar 2015	Low	Low	Low	Unclear	Low	Low	Unclear
Ekman 1998	Low	Unclear	High	Unclear	Unclear	High	Unclear
Fretwell 1990	Unclear	Unclear	Unclear	Unclear	Low	Low	Low
Gary 2010	Unclear	Unclear	High	Low	Low	Low	Unclear
Gitlin 2006	Low	Low	High	Low	Low	Low	Low
Goldberg 2003	Low	Low	High	High	Low	Low	Unclear
Harrison 2002	Low	Low	Low	Unclear	Low	Low	Unclear
Jaarsma 1999	Low	Unclear	Unclear	Low	Low	Low	Unclear
Jerant 2001	Low	Unclear	High	High	Low	High	Unclear
Kasper 2002	Low	Unclear	Unclear	Unclear	Low	Low	Unclear
Krumholz 2002	Unclear	Unclear	High	Low	Low	High	Unclear
Lang 2018	Low	Unclear	High	Low	Low	Low	Unclear
Laramee 2003	Unclear	Unclear	High	High	Unclear	High	Unclear
Ledwidge 2003	High	High	High	Unclear	Low	High	Unclear
Luskin 2002	High	Unclear	High	Unclear	Low	Low	Unclear
Markle-Reid 2010	Low	Low	High	Low	Low	Low	Unclear
McVey 1989	Low	Low	High	Low	High	High	Unclear
Naylor 1994	Low	Low	Low	Unclear	Low	High	Unclear
Naylor 1999	Low	Low	Low	Low	Low	High	Unclear
Northouse 2007	Low	Low	High	High	Low	Low	Low
Pugh 2001	High	High	High	High	High	High	High
Rainville 1999	High	High	Unclear	High	Low	High	High
Rich 1995	Low	Low	Low	Unclear	Low	Low	Unclear

Rich 1993	High	High	High	High	Low	High	High
Riegel 2002	High	High	High	Unclear	Unclear	High	High
Rubenstein 1984	Unclear	Unclear	High	Unclear	High	Low	Unclear
Rubin 1993	Low	Low	High	Low	High	Low	Unclear
Saltvedt 2006	Low	Low	High	Low	High	Low	Low
Serxner 1998	Unclear	Unclear	High	High	High	High	High
Sherwood 2017	Low	Low	High	Unclear	Low	Low	Unclear
Stewart S 1998	Unclear	Unclear	High	High	Low	High	High
Stewart M 1999 (Fox)	High	Unclear	Unclear	Unclear	Unclear	Unclear	Low
Stromberg 2003	Low	Low	High	Low	Low	High	Unclear
Thomas 1993	Low	Low	High	High	High	Low	Low
Trochu 2004	Unclear	Unclear	Unclear	Unclear	Unclear	Unclear	Unclear
Tsuyuki 2004	Low	Low	High	Unclear	Low	Unclear	Unclear
Varma 1999	Unclear	Unclear	Unclear	High	High	High	High
Vidan 2009	High	Unclear	Unclear	Unclear	High	Unclear	Low
Wang 2016	Unclear	Unclear	High	Low	High	Unclear	Unclear
Yu 2010	High	High	High	High	High	Unclear	Unclear
Zeleda 2009	High	Unclear	Unclear	Unclear	High	Unclear	Low
**Integrated Palliative Care**							
Bakitas 2009	Low	High	High	Unclear	Low	Low	Low
Bakitas 2015	Low	Unclear	High	Low	Low	Low	High
Brannstrom 2014	Unclear	Low	High	Low	High	High	High
Edmonds 2010	Low	Low	High	Low	Unclear	Low	Unclear
Given 2002	Low	Unclear	High	High	Unclear	High	Low
Higginson 2014	Low	Low	High	High	Low	Low	Low
Jordhoy 2001	Unclear	Unclear	High	High	Low	Low	Low
Lowther 2015	Low	Low	High	High	Low	Low	Low
Maltoni 2016	Low	Low	High	Unclear	Low	Low	Low
Ozcelik 2014	High	High	High	High	Low	High	Unclear
Rogers 2017	Low	Unclear	High	High	Low	Low	Unclear
Rummans 2006	Low	Low	High	Low	Low	Low	Low
Sidebottom 2015	Unclear	Unclear	High	High	Low	Low	Low
Steel 2016	Low	Low	High	High	Low	High	High
Tattersall 2014	Low	Low	High	Unclear	High	Unclear	Unclear
Temel 2010	Low	High	High	Unclear	Low	Low	Low
Temel 2017	Low	Low	High	Low	Low	Unclear	Unclear
Wong 2016	Low	Low	Unclear	High	Low	High	Low
Zimmerman 2014	Low	High	Low	High	Low	Low	Low

References

1. Applegate, W.B., et al., *A randomized, controlled trial of a geriatric assessment unit in a community rehabilitation hospital.* New England Journal of Medicine, 1990. **322**(22): p. 1572-1578.
2. Asplund, K., et al., *Geriatric-based versus general wards for older acute medical patients: a randomized comparison of outcomes and use of resources.* Journal of the American Geriatrics Society, 2000. **48**(11): p. 1381-1388.
3. Austin, J., et al., *Randomised controlled trial of cardiac rehabilitation in elderly patients with heart failure.* European Journal of Heart Failure, 2005. **7**(3): p. 411-417.

4. Barnes, D.E., et al., *Acute care for elders units produced shorter hospital stays at lower cost while maintaining patients' functional status.* Health Affairs, 2012. **31**(6): p. 1227-1236.
5. Blue, L., et al., *Randomised controlled trial of specialist nurse intervention in heart failure.* BMJ, 2001. **323**(7315): p. 715-718.
6. Burton, E., et al., *Effectiveness of a lifestyle exercise program for older people receiving a restorative home care service: a pragmatic randomized controlled trial.* Clinical interventions in aging, 2013. **8**: p. 1591.
7. Capomolla, S., et al., *Cost/utility ratio in chronic heart failure: comparison between heart failure management program delivered by day-hospital and usual care.* Journal of the American College of Cardiology, 2002. **40**(7): p. 1259-1266.
8. Chang, B.-H., et al., *A relaxation response randomized trial on patients with chronic heart failure.* Journal of Cardiopulmonary Rehabilitation and Prevention, 2005. **25**(3): p. 149-157.
9. Clark, M.M., et al., *Randomized controlled trial of maintaining quality of life during radiotherapy for advanced cancer.* Cancer, 2013. **119**(4): p. 880-887.
10. Clemson, L., et al., *The effectiveness of a community-based program for reducing the incidence of falls in the elderly: A randomized trial.* Journal of the American Geriatrics Society, 2004. **52**(9): p. 1487-1494.
11. Clemson, L., et al., *Integration of balance and strength training into daily life activity to reduce rate of falls in older people (the LiFE study): randomised parallel trial.* Bmj, 2012. **345**: p. e4547.
12. Cline, C., et al., *Cost effective management programme for heart failure reduces hospitalisation.* Heart, 1998. **80**(5): p. 442-446.
13. Close, J., et al., *Prevention of falls in the elderly trial (PROFET): a randomised controlled trial.* The Lancet, 1999. **353**(9147): p. 93-97.
14. Collard, A.F., S.S. Bachman, and D.F. Beatrice, *Acute care delivery for the geriatric patient: an innovative approach.* QRB. Quality review bulletin, 1985. **11**(6): p. 180-185.
15. Counsell, S.R., et al., *Geriatric care management for low-income seniors: a randomized controlled trial.* Jama, 2007. **298**(22): p. 2623-2633.
16. Covinsky, K.E., et al., *Do acute care for elders units increase hospital costs? A cost analysis using the hospital perspective.* Journal of the American Geriatrics Society, 1997. **45**(6): p. 729-734.
17. de Lusignan, S., et al., *Compliance and effectiveness of 1 year's home telemonitoring. The report of a pilot study of patients with chronic heart failure.* European journal of heart failure, 2001. **3**(6): p. 723-730.
18. Doughty, R.N., et al., *Randomized, controlled trial of integrated heart failure management. The Auckland Heart Failure Management Study.* European Heart Journal, 2002. **23**(2): p. 139-146.
19. Dunbar, S.B., et al., *Randomized clinical trial of an integrated self-care intervention for persons with heart failure and diabetes: quality of life and physical functioning outcomes.* Journal of cardiac failure, 2015. **21**(9): p. 719-729.
20. Ekman, I., et al., *Feasibility of a nurse-monitored, outpatient-care programme for elderly patients with moderate-to-severe, chronic heart failure.* European Heart Journal, 1998. **19**(8): p. 1254-1260.
21. Fretwell, M.D., et al., *The senior care study: A controlled trial of a consultative/unit-based geriatric assessment program in acute care.* Journal of the American Geriatrics Society, 1990. **38**(10): p. 1073-1081.
22. Gary, R.A., et al., *Combined exercise and cognitive behavioral therapy improves outcomes in patients with heart failure.* Journal of psychosomatic research, 2010. **69**(2): p. 119-131.

23. Gitlin, L.N., et al., *A randomized trial of a multicomponent home intervention to reduce functional difficulties in older adults.* Journal of the American Geriatrics Society, 2006. **54**(5): p. 809-816.
24. Goldberg, L.R., et al., *Randomized trial of a daily electronic home monitoring system in patients with advanced heart failure: the Weight Monitoring in Heart Failure (WHARF) trial.* American heart journal, 2003. **146**(4): p. 705-712.
25. Harrison, M.B., et al., *Quality of life of individuals with heart failure: a randomized trial of the effectiveness of two models of hospital-to-home transition.* Medical care, 2002: p. 271-282.
26. Jaarsma, T., et al., *Effects of education and support on self-care and resource utilization in patients with heart failure.* European heart journal, 1999. **20**(9): p. 673-682.
27. Jerant, A.F., R. Azari, and T.S. Nesbitt, *Reducing the Cost of Frequent Hospital Admissions for Congestive Heart Failure: A Randomized Trial of a Home Telecare Intervention.* Medical Care, 2001. **39**(11): p. 1234-1245.
28. Kasper, E.K., et al., *A randomized trial of the efficacy of multidisciplinary care in heart failure outpatients at high risk of hospital readmission.* Journal of the American College of Cardiology, 2002. **39**(3): p. 471-480.
29. Krumholz, H.M., et al., *Randomized trial of an education and support intervention to preventreadmission of patients with heart failure.* Journal of the American College of Cardiology, 2002. **39**(1): p. 83-89.
30. Lang, C.C., et al., *A randomised controlled trial of a facilitated home-based rehabilitation intervention in patients with heart failure with preserved ejection fraction and their caregivers: the REACH-HFpEF Pilot Study.* BMJ open, 2018. **8**(4): p. e019649.
31. Laramee, A.S., et al., *Case Management in a Heterogeneous Congestive Heart Failure Population: A Randomized Controlled Trial.* JAMA Internal Medicine, 2003. **163**(7): p. 809-817.
32. Ledwidge, M., et al., *Is multidisciplinary care of heart failure cost-beneficial when combined with optimal medical care?* European Journal of Heart Failure, 2003. **5**(3): p. 381-389.
33. Luskin, F., et al., *A controlled pilot study of stress management training of elderly patients with congestive heart failure.* Preventive cardiology, 2002. **5**(4): p. 168-174.
34. Markle-Reid, M., et al., *The effects and costs of a multifactorial and interdisciplinary team approach to falls prevention for older home care clients 'at risk' for falling: a randomized controlled trial.* Canadian Journal on Aging/La Revue canadienne du vieillissement, 2010. **29**(1): p. 139-161.
35. McVey, L.J., et al., *Effect of a geriatric consultation team on functional status of elderly hospitalized patients: a randomized, controlled clinical trial.* Annals of internal medicine, 1989. **110**(1): p. 79-84.
36. Naylor, M., et al., *Comprehensive discharge planning for the hospitalized elderly: a randomized clinical trial.* Annals of internal Medicine, 1994. **120**(12): p. 999-1006.
37. Naylor, M.D., et al., *Comprehensive Discharge Planning and Home Follow-up of Hospitalized EldersA Randomized Clinical Trial.* JAMA, 1999. **281**(7): p. 613-620.
38. Northouse, L.L., et al., *Randomized clinical trial of a family intervention for prostate cancer patients and their spouses.* Cancer: Interdisciplinary International Journal of the American Cancer Society, 2007. **110**(12): p. 2809-2818.
39. Pugh, L.C., et al., *Case management for elderly persons with heart failure: the quality of life and cost outcomes.* MedSurg Nursing, 2001. **10**(2): p. 71.
40. Rainville, E.C., *Impact of pharmacist interventions on hospital readmissions for heart failure.* American Journal of Health-System Pharmacy, 1999. **56**(13): p. 1339-1342.

41. Rich, M.W., et al., *A Multidisciplinary Intervention to Prevent the Readmission of Elderly Patients with Congestive Heart Failure.* New England Journal of Medicine, 1995. **333**(18): p. 1190-1195.
42. Rich, M.W., et al., *Prevention of readmission in elderly patients with congestive heart failure.* Journal of General Internal Medicine, 1993. **8**(11): p. 585-590.
43. Riegel, B., et al., *Effect of a Standardized Nurse Case-Management Telephone Intervention on Resource Use in Patients With Chronic Heart Failure.* JAMA Internal Medicine, 2002. **162**(6): p. 705-712.
44. Rubenstein, L.Z., et al., *Effectiveness of a geriatric evaluation unit: a randomized clinical trial.* New England Journal of Medicine, 1984. **311**(26): p. 1664-1670.
45. Rubin, C.D., et al., *A randomized, controlled trial of outpatient geriatric evaluation and management in a large public hospital.* Journal of the American Geriatrics Society, 1993. **41**(10): p. 1023-1028.
46. Saltvedt, I., et al., *Randomised trial of in-hospital geriatric intervention: impact on function and morale.* Gerontology, 2006. **52**(4): p. 223-230.
47. Serxner, S., M. Miyaji, and J. Jeffords, *Congestive heart failure disease management study: a patient education intervention.* Congestive Heart Failure, 1998. **4**: p. 23-28.
48. Sherwood, A., et al., *Effects of coping skills training on quality of life, disease biomarkers, and clinical outcomes in patients with heart failure: a randomized clinical trial.* Circulation: Heart Failure, 2017. **10**(1): p. e003410.
49. Stewart, S., S. Pearson, and J.D. Horowitz, *Effects of a Home-Based Intervention Among Patients With Congestive Heart Failure Discharged From Acute Hospital Care.* JAMA Internal Medicine, 1998. **158**(10): p. 1067-1072.
50. Stewart, M., et al., *The impact of a geriatrics evaluation and management unit compared to standard care in a community teaching hospital.* Maryland medical journal (Baltimore, Md.: 1985), 1999. **48**(2): p. 62-67.
51. Strömberg, A., et al., *Nurse-led heart failure clinics improve survival and self-care behaviour in patients with heart failure: Results from a prospective, randomised trial.* European Heart Journal, 2003. **24**(11): p. 1014-1023.
52. Thomas, D.R., R. Brahan, and B.P. Haywood, *Inpatient community-based geriatric assessment reduces subsequent mortality.* Journal of the American Geriatrics Society, 1993. **41**(2): p. 101-104.
53. Trochu, J., S. Baleynaud, and G. Mialet, *Efficacy of a multidisciplinary management of chronic heart failure patients: one year results of a multicentre randomized trial in French medical practice.* Eur Heart J, 2004.
54. Tsuyuki, R.T., et al., *A multicenter disease management program for hospitalized patients with heart failure.* Journal of Cardiac Failure, 2004. **10**(6): p. 473-80.
55. Varma, S., et al., *Pharmaceutical care of patients with congestive heart failure: interventions and outcomes.* Pharmacotherapy: The Journal of Human Pharmacology and Drug Therapy, 1999. **19**(7): p. 860-869.
56. Vidán, M.T., et al., *An intervention integrated into daily clinical practice reduces the incidence of delirium during hospitalization in elderly patients.* Journal of the American Geriatrics Society, 2009. **57**(11): p. 2029-2036.
57. Wang, T.-C., et al., *Effects of a supportive educational nursing care programme on fatigue and quality of life in patients with heart failure: a randomised controlled trial.* European Journal of Cardiovascular Nursing, 2016. **15**(2): p. 157-167.
58. Yu, D.S., D.T. Lee, and J. Woo, *Improving health-related quality of life of patients with chronic heart failure: effects of relaxation therapy.* Journal of advanced nursing, 2010. **66**(2): p. 392-403.
59. Zelada, M.A., R. Salinas, and J.J. Baztán, *Reduction of functional deterioration during hospitalization in an acute geriatric unit.* Archives of gerontology and geriatrics, 2009. **48**(1): p. 35-39.

60. Bakitas, M., et al., *Effects of a palliative care intervention on clinical outcomes in patients with advanced cancer: the Project ENABLE II randomized controlled trial.* Jama, 2009. **302**(7): p. 741-749.
61. Bakitas, M.A., et al., *Early versus delayed initiation of concurrent palliative oncology care: patient outcomes in the ENABLE III randomized controlled trial.* Journal of Clinical Oncology, 2015. **33**(13): p. 1438.
62. Brännström, M. and K. Boman, *Effects of person-centred and integrated chronic heart failure and palliative home care. PREFER: a randomized controlled study.* European journal of heart failure, 2014. **16**(10): p. 1142-1151.
63. Edmonds, P., et al., *Palliative care for people severely affected by multiple sclerosis: evaluation of a novel palliative care service.* Multiple Sclerosis Journal, 2010. **16**(5): p. 627-636.
64. Given, B., et al. *Pain and fatigue management: results of a nursing randomized clinical trial*. in *Oncology nursing forum*. 2002.
65. Higginson, I.J., et al., *An integrated palliative and respiratory care service for patients with advanced disease and refractory breathlessness: a randomised controlled trial.* Lancet Respiratory Medicine, 2014. **2**(12): p. 979-987.
66. Jordhøy, M.S., et al., *Quality of life in palliative cancer care: results from a cluster randomized trial.* Journal of Clinical Oncology, 2001. **19**(18): p. 3884-3894.
67. Lowther, K., et al., *Nurse-led palliative care for HIV-positive patients taking antiretroviral therapy in Kenya: a randomised controlled trial.* The lancet HIV, 2015. **2**(8): p. e328-e334.
68. Maltoni, M., et al., *Systematic versus on-demand early palliative care: results from a multicentre, randomised clinical trial.* European Journal of Cancer, 2016. **65**: p. 61-68.
69. Ozcelik, H., et al., *Examining the effect of the case management model on patient results in the palliative care of patients with cancer.* American Journal of Hospice and Palliative Medicine®, 2014. **31**(6): p. 655-664.
70. Rogers, J.G., et al., *Palliative care in heart failure: the PAL-HF randomized, controlled clinical trial.* Journal of the American College of Cardiology, 2017. **70**(3): p. 331-341.
71. Rummans, T.A., et al., *Impacting quality of life for patients with advanced cancer with a structured multidisciplinary intervention: a randomized controlled trial.* Journal of Clinical Oncology, 2006. **24**(4): p. 635-642.
72. Sidebottom, A.C., et al., *Inpatient palliative care for patients with acute heart failure: outcomes from a randomized trial.* Journal of palliative medicine, 2015. **18**(2): p. 134-142.
73. Steel, J.L., et al., *W eb-based collaborative care intervention to manage cancer-related symptoms in the palliative care setting.* Cancer, 2016. **122**(8): p. 1270-1282.
74. Tattersall, M., et al., *Early contact with palliative care services: A randomised trial in patients with newly detected incurable metastatic cancer.* 2014.
75. Temel, J.S., et al., *Early palliative care for patients with metastatic non–small-cell lung cancer.* New England Journal of Medicine, 2010. **363**(8): p. 733-742.
76. Temel, J.S., et al., *Effects of early integrated palliative care in patients with lung and GI cancer: a randomized clinical trial.* Journal of Clinical Oncology, 2017. **35**(8): p. 834.
77. Wong, F.K.Y., et al., *Effects of a transitional palliative care model on patients with end-stage heart failure: a randomised controlled trial.* Heart, 2016. **102**(14): p. 1100-1108.
78. Zimmermann, C., et al., *Early palliative care for patients with advanced cancer: a cluster-randomised controlled trial.* The Lancet, 2014. **383**(9930): p. 1721-1730.
