# Supplementary material for: Common elements of service delivery models that optimise quality of life and health service use among older people with advanced progressive conditions: a tertiary systematic review
Source: BMJ Open. 2021 Nov 30;11(12):e048417. doi: 10.1136/bmjopen-2020-048417 (PMC8638152; doi:10.1136/bmjopen-2020-048417)
Supplement: Author's manuscript [file bmjopen-2020-048417.draft_revisions.pdf]

BMJ Open

BMJ Open is committed to open peer review. As part of this commitment we make the peer review history of every article we publish publicly available.

When an article is published we post the peer reviewers' comments and the authors' responses online. We also post the versions of the paper that were used during peer review. These are the versions that the peer review comments apply to.

The versions of the paper that follow are the versions that were submitted during the peer review process. They are not the versions of record or the final published versions. They should not be cited or distributed as the published version of this manuscript.

BMJ Open is an open access journal and the full, final, typeset and author-corrected version of record of the manuscript is available on our site with no access controls, subscription charges or pay-per-view fees (http://bmjopen.bmj.com).

If you have any questions on BMJ Open's open peer review process please email info.bmjopen@bmj.com

BMJ Open

Common elements of service delivery models that optimise quality of life and health service use in ageing populations with advanced progressive conditions: a tertiary systematic review

Journal:	*BMJ Open*
Manuscript ID	bmjopen-2020-048417
Article Type:	Original research
Date Submitted by the Author:	04-Jan-2021

Complete List of Authors:	Bayly, Joanne; King's College London, Cicely Saunders Institute for Palliative Care, Policy and Rehabilitation; St Barnabas Hospice<br>Bone, Anna; King's College London, Cicely Saunders Institute for Palliative Care, Policy and Rehabilitation<br>Ellis-Smith, Clare; King's College London, Cicely Saunders Institute for Palliative Care, Policy and Rehabilitation<br>Tunnard, India; King's College London, Cicely Saunders Institute for Palliative Care, Policy and Rehabilitation<br>Yaqub, Shuja; King's College London, Cicely Saunders Institute for Palliative Care, Policy and Rehabilitation<br>Yi, Deokhee; King's College London, Cicely Saunders Institute for Palliative Care, Policy and Rehabilitation<br>Bashan Nkhoma, Kennedy; King's College London, Florence Nightingale Faculty of Nursing Midwifery and Palliative Care<br>Cook, Amelia; King's College London, Cicely Saunders Institute for Palliative Care, Policy and Rehabilitation<br>Combes, Sarah; King's College London, Florence Nightingale Faculty of Nursing, Midwifery & Palliative Care ; St Christopher's Hospice<br>Bajwah, Sabrina; King's College London, Cicely Saunders Institute for Palliative Care, Policy and Rehabilitation<br>Harding, Richard; King's College London, of Palliative Care, Policy and Rehabilitation<br>Nicholson, Caroline; University of Surrey Faculty of Health and Medical Sciences; St Christopher's Hospice<br>Normand, Charles; King's College London, Cicely Saunders Institute for Palliative Care, Policy and Rehabilitation; The University of Dublin Trinity College, Centre for Health Policy and Management<br>Ahuja, Shalini; King's College London Institute of Psychiatry Psychology and Neuroscience, Health Service and Population Research Department<br>Turrillas, Pamela; King's College London, Cicely Saunders Institute for Palliative Care, Policy and Rehabilitation<br>Kizawa, Yoshiyuki; Kobe University, Department of Palliative Medicine<br>Morita, Tatsuya; Seirei Mikatahara Hospital, Palliative and Supportive Care Division<br>Nishiyama, Nanako; Osaka Prefecture University, Graduate School of Comprehensive Rehabilitation<br>Tsuneto, Saturo; Kyoto University Hospital, Department of Human Health Sciences, Department of Palliative Medicine

Ong, Paul; World Health Organization Centre for Health Development
Higginson, Irene; King's College London, Cicely Saunders Institute for Palliative Care, Policy and Rehabilitation
Evans, Catherine ; King's College London, Cicely Saunders Institute for Palliative Care, Policy and Rehabilitation; Sussex Community NHS Foundation Trust
Maddocks, Matthew; King's College London, Cicely Saunders Institute for Palliative Care, Policy and Rehabilitation

Keywords: GERIATRIC MEDICINE, PALLIATIVE CARE, Organisation of health services < HEALTH SERVICES ADMINISTRATION & MANAGEMENT, Health policy < HEALTH SERVICES ADMINISTRATION & MANAGEMENT, Quality in health care < HEALTH SERVICES ADMINISTRATION & MANAGEMENT

I, the Submitting Author has the right to grant and does grant on behalf of all authors of the Work (as defined in the below author licence), an exclusive licence and/or a non-exclusive licence for contributions from authors who are: i) UK Crown employees; ii) where BMJ has agreed a CC-BY licence shall apply, and/or iii) in accordance with the terms applicable for US Federal Government officers or employees acting as part of their official duties; on a worldwide, perpetual, irrevocable, royalty-free basis to BMJ Publishing Group Ltd ("BMJ") its licensees and where the relevant Journal is co-owned by BMJ to the co-owners of the Journal, to publish the Work in this journal and any other BMJ products and to exploit all rights, as set out in our licence.

The Submitting Author accepts and understands that any supply made under these terms is made by BMJ to the Submitting Author unless you are acting as an employee on behalf of your employer or a postgraduate student of an affiliated institution which is paying any applicable article publishing charge ("APC") for Open Access articles. Where the Submitting Author wishes to make the Work available on an Open Access basis (and intends to pay the relevant APC), the terms of reuse of such Open Access shall be governed by a Creative Commons licence – details of these licences and which Creative Commons licence will apply to this Work are set out in our licence referred to above.

Other than as permitted in any relevant BMJ Author's Self Archiving Policies, I confirm this Work has not been accepted for publication elsewhere, is not being considered for publication elsewhere and does not duplicate material already published. I confirm all authors consent to publication of this Work and authorise the granting of this licence.

For peer review only

Common elements of service delivery models that optimise quality of life and health service use in ageing populations with advanced progressive conditions: a tertiary systematic review

Joanne Bayly[1,13], Anna E Bone[1], Clare Ellis-Smith[1], India Tunnard[1], Shuja Yaqub[1], Deokhee Yi[1], Kennedy Nkhoma Bashan[1,] Amelia Cook[1,] Sarah Combes[3, 11], Sabrina Bajwah[1], Richard Harding[1], Caroline Nicholson[10,11], Charles Normand[1, 4], Shalini Ahuja[5], Pamela Turrillas[1], Yoshiyuki Kizawa[6], Tatsuya Morita[7], Nanako Nishiyama [8], Satoru Tsuneto[9], Paul Ong[12], Irene J Higginson[1], Catherine J Evans[1,2*] Matthew Maddocks[1*] (*joint senior authors)

Affiliations

1. King's College London, Cicely Saunders Institute of Palliative Care, Policy and Rehabilitation, London, SE5 9PJ, United Kingdom
2. Sussex Community NHS Foundation Trust, Brighton General Hospital, Elm Grove, BN2 3EW, United Kingdom
3. King's College London, Florence Nightingale Faculty of Nursing, Midwifery & Palliative Care, James Clerk Maxwell Building, 57 Waterloo Road, London, SE1 8WA, United Kingdom
4. Centre for Health Policy and Management, Trinity College, University of Dublin, Ireland
5. King's College London, Health Service and Population Research Department, Institute of Psychiatry, Psychology and Neuroscience, De Crespigny Park, London, SE5 8AF, United Kingdom
6. Kobe University, Department of Palliative Medicine, 7-5-2, Kusunokicho, Chuo-ku, Kobe, Hyogo, Japan, 650-0017
7. Seirei Mikatahara Hospital, Palliative and Supportive Care Division, 3453 Mikatahara, Kita, Hamamatsu, 433-8558, Japan
8. Osaka Prefecture University, Graduate School of Comprehensive Rehabilitation, 3-7-30, Habikino, Habikino-city, Osaka, Japan
9. Department of Human Health Sciences; Kyoto University Hospital, Department of Palliative Medicine, 53 Kawaharacho, Shogoin, Sakyo-ku, Kyoto 606-8507, Japan
10. University of Surrey, Faculty of Health and Medical Sciences, Kate Granger Building, Priestley Road, Surrey Research Park, Guildford, Surrey GU2 7XH
11. St Christopher's Hospice, 51-59 Lawrie Park Rd, London SE26 6DZ
12. World Health Organisation Centre for Health Development
13. St Barnabas Hospices, Worthing, West Sussex, UK

Correspondence: Dr Matthew Maddocks & Dr Catherine Evans

Email: matthew.maddocks@kcl.ac.uk Telephone: +44 (0)207 848 5242

For peer review only

Abstract

Introduction: Health and social care services worldwide need to support ageing populations to live well with progressive conditions while adapting to functional decline and finitude. This review aimed to identify and map key elements within effective integrated geriatric and palliative care services; and consider scalability and generalisability to high, low and middle-income countries (LMICs).

Methods: Tertiary systematic review of geriatric or palliative care studies demonstrating evidence of effectiveness on quality-of-life and/or health-service use outcomes in older adults with advanced progressive conditions. Using an established framework for health system analysis, service elements were identified, extracted and descriptively analysed and then using a staged, iterative process to develop a 'common components' logic model. Stakeholder consultation on scalability with experts in geriatric or palliative care from high, middle and low income countries

Results: 78 studies (59 geriatric, 19 palliative) included spanned all WHO regions. Common service elements (≥80% of studies) included collaborative working, on-going assessment, active patient participation, patient/family education and patient self-management. Effective services incorporated patient engagement, patient goal-driven care, and the centrality of patient needs. Stakeholders (n=20) highlighted that wider implementation of such services requires access to skilled, multi-professional teams with sufficient resource to meet patients' needs. Political and societal will to invest and prioritise palliative and geriatric care for older people alongside geographical and socioeconomic barriers influence scalability.

Conclusion: Our logic model establishes common elements of effective services that transcend best practices in geriatric and palliative care to optimize quality of life and/or health service use in older adults with advanced progressive conditions. These apply across the care continuum, from prevention of functional decline to palliative and end of life care. Priority areas for future research include studies conducted in low-income countries, bereavement support for carers, integrated working bridging health and social care, and involvement of volunteers and community-based organisations.

Review Registration number:

PROSPERO CRD42020150252

Key words

Geriatrics, Palliative Care, Delivery of Health Care, Quality of Life, Systematic Reviews

For peer review only

Strengths and limitations of this study:

We combine evidence from effective models of geriatric and palliative care for older people with progressive advanced conditions on trajectory from prevention of functional decline to end of life.

The review was conducted by an inter-disciplinary group representing broad methodological expertise from many regions of the world.

Our common components logic model is a recombination of effective service elements, but we are unable to assert how effectiveness may be influenced by different combinations of components and their interactions.

Stakeholder engagement identified challenges for scalability where country health budgets are inadequate to meet the growing population need, and where multidisciplinary care is often unavailable.

Key Questions:

What is already known?

Globally, increasing numbers of people are living into older age with multiple conditions that reduce health-related quality of life and increase demand on health and social care services. Models of care found to be effective on these outcomes in clinical trials are conventionally offered by either geriatric or palliative care services, with variance in goals for care.

What are the new findings?

Common elements found across both integrated geriatric and integrated palliative care services include: collaborative working, on-going assessment, active patient participation, patient/family education and patient self-management, patient engagement, patient goal-driven care, and the centrality of patient needs.

What do the new findings imply?

Effective services supporting older people living with advanced progressive conditions, from prevention of functional decline to palliative and end-of-life care, can include service elements that transcend current models of integrated geriatric and integrated palliative care. Wider global implementation requires political will to invest in services for older people and

For peer review only

to address societal attitudes as well as geographical and socioeconomic barriers to geriatric and palliative care.

Introduction

Globally, more people are living into old age [1] with the largest proportional increase occurring in the oldest old [2, 3]. By 2050, 80% of older people will be living in low and middle income countries (LMIC)[4] Successes in child and maternal health and infectious diseases pose new challenges for global health [5, 6] as with ageing comes increased risk of multi-morbidity and/or frailty [7], leading to a trajectory of prolonged and uncertain functional decline. Health and social care needs and their impact on physical functioning are more heterogeneous[1] in older populations, shaped by multiple interacting factors related to the individual and their environment. These changes will bring new societal challenges related to health and social care policy, spending, workforce and security, regardless of developmental context.

The WHO Member States' commitment to achieve Universal Health Coverage (UHC) by 2030 provides an opportunity to plan health and social care delivery for the future. The UHC goals include palliative care for the first time [8] as fundamental to achieving UHC. While proactive prevention remains a priority across the health continuum, a shift in health systems is needed to balance disease modifying interventions with services where improving quality of life is the main goal for care is. Access to appropriate care and support, up to and including the end of life is recognised as a basic human right [9], yet access varies according to socioeconomic and geographic variables [10, 11]. Health systems must align services for older populations to support the dual priorities of living well while adapting to a gradual decline in function. Budget constraints require maximum value from the resources used to improve outcomes [12]. The importance of integrated working within and between services is consistently advocated in global guidance on health service provision for advanced disease[13] and older people [14].

Our previous meta-review outlined service delivery models of integrated geriatric care and integrated palliative care for older people at the end of life [14]. Both showed potential to improve quality of life and patterns of health service use, but with differing emphasis on either function or symptoms and concerns. Our findings underscored the imperative of access to services based on the likelihood of benefit, and integration of services using comprehensive assessment, case management, and/or collaborative working [14]. However, use of systematic reviews as the unit of analysis prevented a detailed description of model

For peer review only

elements and linkages with outcomes, and supressed the heterogeneity across the primary studies. This limited a clear delineation of what worked, how and in what circumstances.

This review therefore aimed to detail service delivery models that optimise quality of life and health services use for older people with advanced progressive conditions to contribute knowledge relevant to healthcare services and systems. Our objectives were to: i) identify and map the key elements of effective service delivery models within primary studies; ii) outline the similarities and differences across models lead by geriatric care or palliative care; and iii) consider the scalability and generalisability of effective models attending to implementation and economic requirements.

Method

Study Design

We conducted a tertiary review of primary studies within our previous meta-review [14], conducted in accordance with the Preferred Reporting Items for Systematic Reviews and Meta-Analysis [15]. We then used logic modelling [16] and a stakeholder consultation to support the analysis and interpretation [17] of the review findings. The work was registered on PROSPERO [CRD42020150252] prior to data extraction.

Patient and Public Involvement

Patients and members of the public were not involved in the design, conduct, reporting or dissemination of this research.

Search strategy

After updating our original search in October 2019 (Supplementary material 1 and 2). We identified primary studies from systematic reviews with a meta-analysis demonstrating overall effectiveness on quality of life (including symptom burden and function) and/or health service use outcomes. The purpose was to include primary studies with empirical evidence of effect on the selected outcomes. Inclusion criteria for primary studies comprised: i) used an experimental study design; ii) contributed to the meta-analysis; and iii) reported a point estimate of effect in the same direction as the meta-analysis. One reviewer (JB) evaluated all systematic reviews and primary studies for eligibility and a second (MM, AB or CES) double-screened studies, with inconsistencies resolved by consensus. Duplicate primary studies were identified and removed.

Data extraction

Data on study population, outcomes and context were extracted. Service delivery models were classified as either integrated geriatric or palliative care in accordance with our meta-

For peer review only

review. The review aimed to inform thinking across healthcare services and systems. To support this, data identification and extraction was informed by a framework for systems analysis, the CATWOE Checklist (customers, actors, transformation processes, world view, owner, environmental constraints) [18, 19]. The list of elements for each CATWOE system component was informed by the TIDieR checklist for complex health service interventions [20] and studies on geriatric [21], integrated [22], transitional [23] and palliative care [24]. The final elements and definitions were agreed by the review team (Supplementary material 3). Each element was categorized as present, absent, or unclear by the research team (JB, MM, AB, CE, DY, CES, SB, NK, SY) and reviewed as a team. Data extraction included supplementary materials and published protocols to support data interpretation.

Quality appraisal

The methodological quality of systematic reviews and primary studies was appraised using the AMSTAR tool [25] and Cochrane Risk of Bias Tool respectively [26]. We used the quality appraisal in the systematic reviews when the Cochrane Risk of Bias Tool was used, otherwise assessment was by two researchers (JB, IT). We did not exclude studies from analysis based on quality.

Development of logic model

We used a staged and iterative approach following Rohwer et al's guidance on logic models for complex health interventions [16] incorporating analysis of extracted data followed by a stakeholder consultation.

The frequency and proportion of the key service elements [18, 19] was summarised overall and for integrated geriatric and palliative care models separately. The proportion was calculated using studies where the element was categorized as present or absent. We mapped service elements present in ≥50% of integrated geriatric and/or palliative care models to existing logic templates [16]. From the CATWOE framework used for data extraction: 'customer' elements were mapped under a population heading; with actors and environmental constraints: human resources mapped as 'service delivery'. Transformation processes were mapped as 'service components' and conceptual model elements as 'approach to service delivery'. Owner and environmental constraint elements were mapped under a 'context' and 'implementation' headings. To compare the presence of elements between integrated geriatric and palliative care models we conducted chi squared or Fisher's exact tests.

We appraised the potential for the components of effective interventions to be generalised and scalable, defined as the ability "to be expanded under real world conditions to reach a greater proportion of the eligible population while retaining effectiveness"[27]. We shared the

For peer review only

interim logic model and consulted a purposive sample of geriatric and palliative healthcare researchers, clinical-academics and clinicians from high, middle and low income countries with expertise in either geriatric or palliative care. These stakeholders were asked to consider the barriers and facilitators to provide the elements of care as detailed in their respective country and healthcare setting. We used the Context and Implementation of Complex Interventions Framework (CICI) to structure a response form, to simplify the structural complexity in understanding the context, implementation and the setting in an integrated manner[28]. Context and implementation considerations relating to scalability and generalisability were extracted from collated narrative responses and summarised under the CICI framework domains. We combined the data analysis and stakeholder consultation findings and through team discussion and consensus built a common components logic model to represent the data[16].

Results

Study retrieval

Ten systematic reviews met eligibility; seven from the meta-review [29-35] and three [36-38] from the updated search. The reviews reported 180 potentially eligible studies, of which 47 were duplicates which were removed. Of the 133 remaining studies, 78 met eligibility (Figure 1).

Characteristics of included studies

Of the 78 included studies, 59 were categorised as integrated geriatric care and 19 as Integrated Palliative Care (Table 1 and Supplementary material 4). All WHO regions were represented, though studies were predominantly from the North American region of the Americas (n=46), or Europe (n=22), with fewer from Western Pacific (n=6), South East Asia (n=3) and only a single study from Africa. The large majority were from high income countries (n=75). The number of study participants ranged from 20 to 1632, with data available from 17,739 participants overall. Nearly half of all studies recruited patients with heart failure (n=36) and one-third recruited patients with no main or multiple diagnoses (n=26). Palliative care studies recruited by diagnosis, most often cancer (n=12). Studies interventions were delivered across multiple care settings (n=31), in participants' homes (n=15) or in hospital (outpatients n=14; inpatients n=12) (Table 1).

For peer review only

***Table 1**. Summary characteristics of included studies N=78*

Variable		Frequency		
		All n=78	Geriatric n=59	Palliative n=19
**WHO region**	Americas	46	36	10
	Europe	22	16	6
	South East Asia	3	2	1
	West Pacific	6	5	1
	Africa	1	1	0
**Country income status**	High	75	17	58
	Upper - middle	2	1	1
	Lower – middle	1	0	1
	Low	0	0	0
**Population by main diagnosis**	Heart failure	36	32	4
	No main diagnosis	23	23	0
	Cancer	14	2	12
	Single	4	1	3
	Mixed	10	1	9
	Heart failure + diabetes	1	1	0
	Heart failure + depression	1	1	0
	Multiple Sclerosis	1	0	1
	Multiple diagnosis (COPD, cancer, HF, ILD, MND)	1	0	1
	HIV infection	1	0	1
**Population by referral criteria**	People with heart failure	38	34	4
	People with acute episode of illness	17	17	0
	People with advanced cancer	13	2	11
	Older people (varied age ranges)	6	6	0
	People with HIV	1	0	1
	People with multiple sclerosis	1	0	1
	Advanced mixed diagnoses	1	0	1
	People with cancer commencing chemotherapy	1	1	1
**Health organisation funding**	State funded health organisation	35	26	9
	For profit health organisation	37	28	9
	Non-profit health organisation	6	5	1
**Care Setting**	Mixed settings	29	20	9
	Hospital in-patients and home	6	6	0
	Hospital in-patients and out-patients	5	5	0
	Hospital out-patients and home	10	4	6
	Hospital in-patients, out-patients and home	7	4	3
	Hospital emergency room and home	1	1	0
	Home	16	13	3
	Hospital out-patients	15	9	6
	Hospital in-patients	13	12	1
	Community settings	3	3	0

COPD =Chronic Obstructive Pulmonary Disease, HF = Heart failure, MND = Motor neurone disease, ILD = Interstitial lung disease.

For peer review only

Quality appraisal

The ten systematic reviews were assessed as of moderate quality (Supplementary material 5). Six reviews reported Cochrane Risk of Bias tool to assess methodological quality of included studies [29, 31, 32, 35, 37, 38]. Overall findings suggest a low to moderate risk of bias (Supplementary material 6). High risks were associated with performance and detection bias, most frequently related to challenges of blinding participants and personnel. Selective reporting bias reflected the number of unregistered studies with no published protocol. Risk of bias tended to be lower for palliative care compared to geriatric care studies (Supplementary material 6).

Service delivery elements

Most services used several methods to support integrated working between professionals and specialities, most frequently collaborative working and case management (Table 2). Common service delivery model elements, present in more than 80% of studies, were professional education of staff, (staff who have received nationally recognised and regulated training and education), on-going assessment, active patient participation, and evidence of patient engagement in their care. The least common elements overall were bereavement support, 24-hour home visits or access to physicians, links to residential hospice facilities, and joint provision of care across health and social care services. No studies reported delivering interventions in residential care/nursing homes or use of volunteers. Comparing between integrated geriatric and palliative care, palliative care services had a higher frequency of end of life expertise and training, professional psychosocial, spiritual support and physician home visits. In contrast geriatric care services had more frequent evidence of early rehabilitation assessment and self-management, though the differences were not statistically significant (Table 2).

For peer review only

Table 2. Service delivery model elements N=78

	All n (%)	Geriatric n (%)	Palliative n (%)	Sig.
Method of supporting integrated working				
Collaborative working	64 (82)	46 (78)	18 (95)	0.17*
Case management	61 (78)	46 (78)	15 (79)	1.00*
Comprehensive assessment	51 (65)	36 (68)	15 (79)	0.36
Actors-Workforce				
Professional Education	76 (100)	58 (100)	18 (100)	1.00
MDT Care	54 (72)	42 (73)	12 (71)	1.00*
Rehabilitation expertise training	34 (50)	27 (50)	7 (50)	1.00
End of life expertise training	18 (25)	1 (2)	17 (90)	<0.001*
Transformation- Service Model elements / components				
Patient family education	60 (100)	49 (100)	11 (100)	0.93
Medication review	51 (80)	40 (77)	11 (92)	0.43*
Self-management	48 (80)	41 (84)	7 (64)	0.21*
Systematic risk screening	47 (69)	37 (70)	10 (67)	1.00*
Contact with GP or attending doctor	46 (68)	33 (65)	13 (77)	0.37
Practical Support	41 (68)	34 (69)	7 (64)	0.73*
Medical intervention	52 (67)	39 (66)	13 (68)	0.85
Individualised MDT plan	40 (61)	29 (59)	11 (65)	0.69
Complex/medication management	37 (58)	30 (59)	7 (54)	0.75
Discharge planning	36 (52)	29 (55)	7 (44)	0.44
Professional psychosocial support	38 (51)	26 (44)	12 (80)	0.01
Team case rounds	25 (40)	18 (37)	7 (50)	0.37
Early rehab assessment	25 (38)	21 (40)	4 (29)	0.54
Advanced care planning	23 (30)	9 (16)	14 (78)	<0.001
Emergency response plan	15 (21)	12 (22)	3 (20)	1.00*
Spiritual support	13 (18)	2 (3)	11 (79)	<0.001*
Bereavement Support	4 (5)	0 (0)	4 (25)	0.002*
Transformation- Mode of Delivery				
On-going assessment	66 (87)	50 (86)	16 (89)	1.00*
Face to face & telephone	41 (53)	31 (53)	10 (53)	0.10
Face to face interaction	31 (40)	23 (39)	8 (42)	0.81
Access to inpatient beds	21 (30)	18 (32)	3 (21)	0.53*
Physician home visits	11 (15)	4 (7)	7 (37)	0.04*
24-hour Physician access	6 (10)	5 (11)	1 (7)	1.00*
Telephone only	5 (6)	4 (7)	1 (5)	1.00*
24-hour home visits	1 (1)	1 (2)	0 (0)	1.00*
Online only	1 (1)	1 (2)	0 (0)	0.10*
Transformation-Operational tools & guidance to support practice				
Standard comprehensive assessment	38 (59)	26 (55)	12 (71)	0.27
Worldview- Methods of Integrated Working				
Link to Hospital	57 (78)	41 (72)	16 (100)	0.02*
Expert consult with other providers	40 (58)	24 (45)	16 (100)	<0.001
Link between community services	31 (50)	22 (45)	9 (69)	0.12
Joint provision-health & social care	7 (10)	4 (7)	3 (20)	0.16*
Link to residential hospice	5 (7)	1 (2)	4 (27)	0.005*
Worldview-Conceptual Model				
Patient engagement	71 (99)	53 (98)	18 (100)	1.00*

For peer review only

Active patient participation	67 (99)	50 (98)	17 (100)	1.00*
Centrality of patient needs	64 (91)	46 (89)	18 (100)	0.33*
Patient goal driven care	56 (81)	40 (77)	16 (94)	0.16*
Ongoing / continuous care	46 (67)	33 (62)	13 (81)	0.16
Joint decision making	38 (69)	25 (61)	13 (93)	0.04*
Service driven care planning	38 (54)	34 (65)	4 (21)	0.001*
Needs and benefit-driven care planning	33 (46)	18 (35)	15 (79)	0.001
Caregiver engagement	32 (55)	22 (50)	10 (71)	0.16

Service delivery agents

All interventions were delivered by qualified health care professionals and in >70% of studies working in multi-disciplinary teams. Over 90% of studies involved trained medical and nursing clinicians and 59% involved members of the wider health care team, including physiotherapists, occupational therapists and social workers. Geriatric care studies involved physicians from geriatrics, cardiology and general practice, whereas palliative care studies involved physicians from cardiology, neurology, respiratory medicine, oncology, psychiatry, primary care and palliative medicine. While physiotherapists were reported across all studies, fewer occupational therapists and dietitians were reported in those from palliative care. No studies reported the involvement of volunteers (Table 3).

For peer review only

Table 3. *Service delivery model agents*

Delivery Agent	All n (%)	Geriatric n (%)	Palliative n (%)	Sig.
Physicians				
**Geriatrician**	**14 (18)**	**14 (24)**	**0 (0)**	**0.02**
Cardiologist	15 (19)	12 (20)	3 (16)	1.0
**Palliative care physician**	**12 (15)**	**0 (0)**	**12 (63)**	**<0.001***
Neurologist	1 (1)	0 (0)	1 (5)	0.24*
Respiratory physician	1 (1)	0 (0)	1 (5)	0.24*
**Oncologist**	**4 (5)**	**0 (0)**	**4 (21)**	**0.001***
Psychiatrist	2 (3)	0 (0)	2 (11)	0.06*
Physician	18 (23)	17 (29)	1 (5)	0.06*
Primary care doctor (GP)	5 (6)	4 (7)	1 (5)	0.55*
Physician assistant	2 (3)	2 (3)	0 (0)	0.43*
Nurses				
Nurse	24 (31)	22 (37)	2 (11)	0.28
Advanced nurse practitioner	13 (17)	8 (14)	5 (26)	0.17*
Specialist cardiac nurse	12 (15)	10 (17)	2 (11)	0.40*
Primary care nurse	9 (8)	8 (14)	1 (5)	0.30*
Specialist geriatric nurse	6(8)	6 (10)	0 (0)	0.18*
Case manager	5 (6)	3 (5)	2 (11)	0.35*
Specialist palliative nurse	4 (5)	1 (2)	3 (16)	0.43*
Specialist rehabilitation nurse	1 (1)	1 (2)	0 (0)	0.76*
Specialist HIV nurse	1 (1)	0 (0)	1 (5)	0.24*
Oncology nurse	1 (1)	0 (0)	1 (5)	0.24*
Allied Health Professionals				
Physiotherapist	23 (29)	17 (29)	6 (32)	0.85
Occupational Therapist	14 (18)	12 (20)	2 (11)	0.28*
Dietitian	16 (21)	14 (24)	2 (11)	0.18*
Psychologist	9 (15)	6 (10)	3 (16)	0.38*
Pharmacologist/pharmacist	7 (9)	7 (12)	0 (0)	0.13*
Chaplain	4 (5)	1 (2)	3 (16)	0.43*
Audiologist	1 (1)	1 (2)	0 (0)	0.76*
Speech and language therapist	1 (1)	1 (2)	0 (0)	0.76*
Social Care				
Social worker	21 (27)	17 (29)	4 (21)	0.51
Home care service manager	3 (4)	3 (5)	0 (0)	0.43*
Social assistant	4 (1)	3(5)	1(5)	0.68*
Other professionals				
Unspecified wider 'MDT'	11 (14)	9 (15)	2 (11)	0.47*
Exercise instructor	2 (3)	2 (3)	0 (0)	0.57*

Service outcomes including costs

Forty-five studies (58%) were included based on an effect on quality of life alone. Fifty-seven studies (73%) used a disease or population specific tool to quantify quality of life and five studies (6%) employed the Euro-Qual-5D (EQ-5D). Thirty-three studies (42%) reported utilisation of acute care services (e.g. hospital admission, readmission after discharge) or community care services and 20 studies (26%) calculated costs of health services utilisation. Only a minority (n=12/15%) demonstrated an effect on both quality of life and health service

For peer review only - http://bmjopen.bmj.com/site/about/guidelines.xhtml

use, all of which were geriatric care studies. No study used costs and EQ-5D to generate information required for health economic decision making (Table 4).

Table 4. *Number of studies reporting quality of life and health services use outcomes*

		Health service use			
		None	More than 1	1+ and costs	Sub total
Quality of life	None	0	6	15	21
	More than 1	40	7	5	52
	1+ and EQ-5D	4	0	0	5
	Sub total	45	13	20	78

Common components logic model

The interim logic model highlighted key elements present in the majority (<80%) of included studies. Some elements were more present in integrated palliative care compared to geriatric care studies; professional psychosocial support, advance care planning, care-giver engagement, joint decision making and expert consultation with other providers. In contrast, integrated geriatric care models more often included a social worker or dietician as a delivery agent, and care planning was more often organised around the service with the same intervention delivered to all patients but with customisation and tailoring (Figure 2).

Stakeholder perspectives on scalability

Stakeholders (n=20) with knowledge of hospital, home community and/or home settings across High Income (UK, Japan, Taiwan, Portugal, Chile) and LMICs (Uganda, Malawi, South Africa, Ghana, Zimbabwe, China, India and Bangladesh) contributed views. The context and implementation considerations drawn from their responses on scalability were incorporated into the logic model (Figure 2). The stakeholders described how rapid population aging with the associated rise in multimorbidity, frailty and dementia means patients are typically becoming more complex. This can be challenging to adapt to in LMICs where health services have historically focused on prevention and management of infectious diseases, but populations are rapidly aging and experiencing increased burden of non-communicable disease. Specialist services being often based in major city hospitals was described as a barrier to providing care to rural populations. Recruiting, training and retaining skilled staff to work in rural areas and having a multi-disciplinary team of allied health professionals and specialist doctors and nurses was considered infeasible for many rural areas.

Stakeholders from LMICs considered that overall health budgets were inadequate to meet the population need, and multi-professional care was considered unaffordable. The voluntary sector was often seen as important to augmenting publicly funded services. In some contexts, continuity of care is impeded when individually funded services compete for

For peer review only

resources rather than collaborate. There are challenges to multidisciplinary working in systems in which health workers receive payment directly from patients, financially disincentivising referrals for expert consultation. Social deprivation was cited as an important barrier to accessing care, especially in health systems with out of pocket expenses or private insurance.

Stakeholders described how cultural norms influence care provision. Death denying attitudes in some high and low income cultures influence uptake of palliative care services. Some countries, do not recognise or respect the specialities of palliative care and geriatric care. Whether the family or the health system are considered responsible for care provision varies internationally and is influenced by cultural beliefs, such as filial piety, gender-related norms as well as changing intergenerational family structures and availability. Faith and religion were cited as supportive factors in the provision of both hospices and nursing homes though providing spiritual support, individualised care plans, patient goal-driven care and the centrality of patient needs, though it was recognised that this could be challenging for minority groups. Respondents in some settings reported that joint decision-making and active patient engagement is often not culturally congruent especially for older people, when highly respected health care professionals and/or other family members are expected to direct care. For example, responsibility for engaging with the health care team and decision making is held by adult children in Taiwan and husbands in Malawi.

Increasing education levels and access to the internet were identified as factors that are changing patient and family participation in joint decision-making. Finally, stakeholders recognised an increasing political will to invest in services for older people supported by a growing public and research agenda and established regulatory frameworks. However, this did not always equate to increases in funding. A lack of policies and clinical governance for specialist palliative and geriatric care was reported as a problem, for example tight legal restrictions on opiate prescribing limited medication management.

Discussion

This tertiary review used rigorous methods to identify and map key elements within service delivery models that improve quality of life and/or health service use outcomes for older people with advanced progressive conditions. Common elements included collaborative working between professionals and specialities, on-going assessment, active patient participation, patient/family education and patient self-management. Effective service delivery approaches consistently incorporated patient engagement, patient goal-driven care, and the centrality of patient needs. The final logic model is underpinned by models of public health [1], integrated care for ageing populations[39], and our earlier review [14].

Current thinking proposes that the goals of health and social care should target the optimisation of a person’s intrinsic capacity (the combination of the person’s physical,

For peer review only

mental, psychological and social capacity), and functional ability ("health-related attributes that enable people to be and to do what they have reason to value"[39]) to compress functional decline across the life course from primary prevention through to end of life [39, 40]. Our model encompasses elements that aim to 'protect' (discharge planning and falls prevention programmes), 'reactivate' (disease management, self-management and exercise programmes), 'compensate' (symptom management, support with capabilities for activities of daily living) and 'support' (enhancing social assets and provision of home care). Such interventions may together support older people to maintain intrinsic capacity and functional ability[41] along the care continuum.

With consideration of social determinants, this broader focus extends health and social care beyond episodic provision at points of decline and meets key recommendation for the dual delivery of both geriatric and palliative care[1].

Our findings build on those of previous reviews. Bainbridge et al [24] found that 'linkages with hospital,' 'multi-professional teams' and 'end of life care expertise and training' were critical to the delivery of models of in-home end-of-life care. A review of integrated care approaches for older people by Briggs et al found that although multidisciplinary teams, comprehensive assessment and case management were most frequently reported, no elements were present in more than three quarters of the studies reviewed [22]. Our findings suggest that a capable workforce working collaboratively across disciplinary boundaries, providing comprehensive ongoing assessment with tailored care centred on the needs of individuals[42] is effective on measures of health-related quality of life and/or health service use. Realising this model of care requires active patient engagement, participation and self-management. Multidimensional assessment, including medical, physical, cognitive, social and spiritual components at multiple points over time, allows for a shared understanding of the person and joint decision making to address their priorities in their context. A case management approach where each person is assigned to a designated team or key worker is one means to support this. A large caseload with short periods of contact can limit the level of engagement and impede individualised tailored care. A balance is needed between large caseloads, which enable reach but may constrain impact on outcomes of care.

We provide insights into the range of health and social care providers associated with effective interventions in this population. The most frequently reported care providers within multi-disciplinary teams included physicians, nurses, physiotherapists, general practitioners and social workers. Delivering high quality care demands a broad range of education courses and training for health care professionals in core skills of comprehensive assessment, communication and symptom management specific to individual need. This would include how to support people to acquire self-management skills to live well with a progressive condition and how to equip those close to them with skills to provide care as

For peer review only - http://bmjopen.bmj.com/site/about/guidelines.xhtml

they approach end of life and dying. Investment in training and education is required to ensure the skills base keeps up with this growing population, and work towards greater coverage in countries where formal training opportunities are limited. Uncoupling these skills from specific professional roles and working towards a generalist skills set may be most beneficial. Training and education should however be accompanied by access to specialists to provide supervision and enable continued professional development. It is of note that no studies involved volunteers which likely reflects the dominance of the included studies from high-income countries where reliance on volunteers in health care settings is less common compared with low/middle income countries. Volunteers may provide alternative modes of support for older people that supplement or enhance usual health and social care provision[43, 44]

Service elements that are relevant to intrinsic capacity and functional ability, [1] but not represented in our logic model include joint provision across health and social care; early rehabilitation assessment, and access to in-patient beds. Neglecting social care can have a considerable effect on quality of life for older people, their family and friends, and lead to increased patient and carer morbidity and mortality [45]. Goodwin et al's (2014) synthesis of international integrated care projects posits no single organizational model or approach that best supports integrated care [46]. Of studies detailing any integration, some used follow-up as older people transition from acute to community care and others reported integrated teams in the community [47]. Projected changes in population demographics and workforce issues present challenges for the delivery of high quality care with value for money at the interface of formal and informal health and social care delivery[48].These were highlighted repeatedly in our stakeholder consultation.

Early rehabilitation assessment was detailed in only 40% and 19% of geriatric and palliative care studies respectively. Given maintaining independence, normality and participation in everyday life are high priorities for older people at the end of life [49], this was a surprising finding. This may relate to palliative care's historical focus on physical symptoms arising from advanced disease rather than functional needs, and the presumption that decline is an inevitability of disease progression [50]. The increasing prominence of rehabilitation in palliative care and increasing evidence for the type and timing of rehabilitative interventions for older people is changing this misconception. However, this may not be reflected in the type of evidence within this review, as much current evidence is within feasibility and acceptability studies. Of the 21 papers that indicate dedicated inpatients beds, 57% were from the USA. This may be related to the USA's dual geriatric and palliative medicine training, or a greater policy focus on acute treatment. Dedicated inpatient palliative care beds are less prevalent and may be decreasing in acute hospitals depending on the policy of the country involved. While the availability of inpatient beds is a factor associated with death in hospital [51], home and hospice deaths have often been portrayed as the "best place to

For peer review only

die"[52]. Although individual patient decisions are more complex, home as the preferred place of death at end of life remains a powerful driver influencing policy and service delivery[53].

Methodological reflection

This tertiary review synthesises evidence on effective models of care for older people with progressive advanced chronic conditions from many studies. The review was conducted by a large inter-disciplinary team with a range of methodological expertise and representation from many regions of the world. Included studies targeted different populations, disease related needs, and at specific points on the health care pathway from prevention of functional decline to end of life. The CATWOE framework [19] informed our data extraction, providing a multi-perspective and multi-level framework to consider system-based service delivery. We used the CICI framework and stakeholder engagement to develop a system-based logic model based on our findings.[17]. The final visual logic model highlights key elements to consider during service development across the continuum of care and are applicable to different international settings. We note that Integrated Geriatric Care more frequently delivered interventions that were effective on health service use outcomes. This may reflect study methodology, as health economics in palliative care are more typically explored using routine data and observational studies [54].

While our logic model serves as a useful resource for health systems looking to strengthen their response to population aging and improve care for those near the end of life, it has some limitations. The macro level data presented is limited to country, country income status and systems for funding health care. Other than this, studies seldom provided information to support evaluation of how interventions could be scaled and implemented. Our stakeholder engagement identified that there may be limited applicability for some model components across country settings, especially as no studies were identified from low income countries. Overall health budgets in LMICs are inadequate to meet the growing population need, and multidisciplinary care is often unavailable. Change beyond the health system, into education and health promotion, would be needed to implement such models to meet the challenge of rising incidence in diseases of ageing.[55]

Few studies were identified in non-malignant conditions beyond heart failure. As found in other systematic reviews of complex interventions in this population [18], we were unable to determine the specific mechanisms of action that make each component effective. Some limitations are associated with the construction of our data extraction framework. We did not extract data that explicitly recorded how interventions provided care across care boundaries during care transitions. However, elements, including on-going assessment, expert consultation with other providers and links between community services were present in more than fifty percent of studies, indicating that this may have been occurring. The

For peer review only

intervention portrayed in the logic model is a recombination of intervention components which as a whole remains untested [56]. We are unable to assert how effectiveness may be influenced by different combinations of components and their interactions.

The common components logic model provides data to inform health and social care policy and for the conceptual and organisational development of services. Policy and research recommendations are presented in Box 1.

Box 1. Recommendations

Policy

- configure services for the whole trajectory of chronic progressive conditions to the end of life and move away from a focus on acute episodes of care;
- plan and deliver education to drive provision of a capable workforce. A broad range of professional education courses and training in core skills of geriatric and palliative care, including comprehensive assessment, communication and symptom management specific to individual need is required
- incentivise interdisciplinary and collaborative working between professional disciplines and across health and social care settings, to optimise high-quality individualised service provision and care coordination. This integrated care, when aligned to need rather than diagnostic condition, will increase the reach and impact of services and promote equitable access
- enable robust evaluation by embedding routine outcome measurement in health and social care settings. These should include measures of intrinsic capacity, functional ability, symptom experience and quality of life. Measures should capture the changes in health and social well-being that are associated with the provision of high quality individualised care across the care continuum from protect to support and end of life care

Research

- clinical and cost effectiveness of interventions underpinned by our proposed model should be tested in older people with multi-morbidity based on need, rather than diagnostic condition, over longer trajectories, and across care boundaries
- clinical and cost effectiveness of interdisciplinary rehabilitation and social care interventions targeting older persons and their informal and formal carers to improve, maintain or compensate for declining function
- improved quality of reporting of intervention mechanisms of action at the component level, including linkages with target outcome(s), to support future evaluations and wider implementation if benefit is apparent

For peer review only - http://bmjopen.bmj.com/site/about/guidelines.xhtml

- exploration of the role of volunteers and community based organisations in service delivery models. These should include domains less well addressed, e.g. primary prevention where supporting social well-being and participation may prevent social isolation
- studies to investigate how variance in models of health and social care (including funding) across country setting, influence person-centred and health economic outcomes across the care continuum. Studies in LMICs should be prioritised

Conclusion

Our logic model synthesises common elements of interventions found to be effective for health related quality of life and/or health service use for older people with advanced progressive conditions. Common elements included collaborative working between professionals and specialities, on-going assessment, active patient participation, patient/family education and patient self-management, whilst effective service delivery approaches consistently incorporated patient engagement, patient goal-driven care, and the centrality of patient needs. These elements transcend best practices in geriatric care and palliative care to optimize outcomes across the continuum; from prevention of functional decline to end of life care. The model can inform provision of health and social care aligned to the needs of this rapidly growing population, to reduce suffering for older people across the globe for them to live as well as possible and die with dignity.

Contributions:

JB, AEB, CES, RH, CN, SA, YK, TM, NN, ST, PO, IJH, CJE, MM conceived and designed the study. JB, AEB, CES, IT, DY, KN, AC, SC, SB, CJE, MM extracted data. JB, AEB, CES, AC, RH, KN, CJE, MM analysed data. JB, AEB, AC, SC, CN, PO, CJE, MM drafted the manuscript, All authors critically revised the draft and approved the final manuscript.

Funding

This research was funded by the World Health Organization (WKC-EOLC-K19002) and supported by The Dunhill Medical Trust [grant number RPGF1906\177] and the National Institute of Health Research Applied Research Collaboration South London (NIHR ARC South London) at King's College Hospital NHS Foundation Trust (NIHR200152). AEB is supported by the Dunhill Medical Trust and Cicely Saunders International. MM is funded by an NIHR Career Development Fellowship (CDF-2017-10-009) and CE by a Health Education England/NIHR Senior Clinical Lectureship (ICA-SCL-2015-01-001). IJH is an NIHR Senior Investigator Emeritus. This publication presents independent research funded by the National Institute for Health Research (NIHR). The views expressed in this publication

For peer review only

are those of the author(s) and not necessarily those of the NHS, NIHR or the Department of Health and Social Care.

Competing Interests Statement

Paul Ong reported that he was an employee of the funding sponsor, the World Health Organization, and was involved in the extraction, analysis, and interpretation of data. All other authors have no competing interests.

Data sharing statement

Extracted data is available on request from the corresponding author.

Figure Captions

Figure 1. *PRISMA flowchart for selection of primary studies*

Figure 2. *Common components logic model: Key elements of effective service delivery models for older people with advanced progressive conditions*

For peer review only

References

1. WHO, *World Report on Aging and Health*. 2015, World Health Organisation.
2. Evans, C.J., et al., *Place and cause of death in centenarians: a population-based observational study in England, 2001 to 2010.* PLoS Med, 2014. **11**(6): p. e1001653.
3. Nations, U., *World population prospects: The 2015 revision, key findings and advance tables.* Rep No. ESA/P/WP. 241, 2015.
4. WHO. *Ageing and health*. [cited 2018 21/11/2018]; Available from: http://www.who.int/news-room/fact-sheets/detail/ageing-and-health.
5. Kruk, M.E., et al., *High-quality health systems in the Sustainable Development Goals era: time for a revolution.* Lancet Glob Health, 2018. **6**(11): p. e1196-e1252.
6. Peto, R., A.D. Lopez, and O.F. Norheim, *Halving premature death.* Science, 2014. **345**(6202): p. 1272.
7. Clegg, A., et al., *Frailty in elderly people.* The lancet, 2013. **381**(9868): p. 752-762.
8. World, H., Organisation. *Universal Health Coverage (UHC)*. 2020 [cited 2020 02 07 20]; Available from: https://www.who.int/news-room/fact-sheets/detail/universal-health-coverage-(uhc).
9. Ahmedzai, S.H., et al., *A new international framework for palliative care.* European Journal of Cancer, 2004. **40**(15): p. 2192-2200.
10. Walshe, C., et al., *Patterns of access to community palliative care services: a literature review.* Journal of pain and symptom management, 2009. **37**(5): p. 884-912.
11. Cohen, L.L., *Racial/ethnic disparities in hospice care: a systematic review.* Journal of palliative medicine, 2008. **11**(5): p. 763-768.
12. Porter, M.E., *What is value in health care.* N Engl J Med, 2010. **363**(26): p. 2477-2481.
13. WPCA and WHO, *Global Atlas of Palliative Care at the End of Life*. 2014, World Palliative Care Alliance: London.
14. Evans, C.J., et al., *Service Delivery Models to Maximize Quality of Life for Older People at the End of Life: A Rapid Review.* The Milbank Quarterly, 2019. **97**(1): p. 113-175.
15. Moher, D., et al., *Preferred reporting items for systematic review and meta-analysis protocols (PRISMA-P) 2015 statement.* Systematic reviews, 2015. **4**(1): p. 1.
16. Rohwer, A., et al., *Guidance on the use of logic models in health technology assessments of complex interventions.* International Journal of Technology Assessment in Health Care, 2016.
17. Popay, J., et al., *Guidance on the conduct of narrative synthesis in systematic reviews: A product from the ESRC Methods Programme*. 2006.
18. Brereton, L., et al., *What do we know about different models of providing palliative care? Findings from a systematic review of reviews.* Palliative medicine, 2017. **31**(9): p. 781-797.
19. Checkland, P. and C. Tsouvalis, *Reflecting on SSM: the link between root definitions and conceptual models.* Systems Research and Behavioral Science: The Official Journal of the International Federation for Systems Research, 1997. **14**(3): p. 153-168.
20. Hoffmann, T.C., et al., *Better reporting of interventions: template for intervention description and replication (TIDieR) checklist and guide.* Bmj, 2014. **348**: p. g1687.
21. Fox, M.T., et al., *Acute care for elders components of acute geriatric unit care: systematic descriptive review.* J Am Geriatr Soc, 2013. **61**(6): p. 939-46.
22. Briggs, A.M., et al., *Elements of integrated care approaches for older people: a review of reviews.* BMJ Open, 2018. **8**(4): p. e021194.
23. Naylor, M.D., et al., *Components of Comprehensive and Effective Transitional Care.* J Am Geriatr Soc, 2017. **65**(6): p. 1119-1125.
24. Bainbridge, D., H. Seow, and J. Sussman, *Common Components of Efficacious In-Home End-of-Life Care Programs: A Review of Systematic Reviews.* J Am Geriatr Soc, 2016. **64**(3): p. 632-9.
25. Shea, B.J., et al., *Development of AMSTAR: a measurement tool to assess the methodological quality of systematic reviews.* BMC medical research methodology, 2007. **7**(1): p. 10.
26. Higgins, J.P., et al., *The Cochrane Collaboration's tool for assessing risk of bias in randomised trials.* Bmj, 2011. **343**: p. d5928.

For peer review only - http://bmjopen.bmj.com/site/about/guidelines.xhtml

27. Milat, A.J., et al., *The concept of scalability: increasing the scale and potential adoption of health promotion interventions into policy and practice.* Health promotion international, 2013. **28**(3): p. 285-298.
28. Pfadenhauer, L.M., et al., *Making sense of complexity in context and implementation: the Context and Implementation of Complex Interventions (CICI) framework.* Implementation science, 2017. **12**(1): p. 21.
29. De Coninck, L., et al., *Home- and Community-Based Occupational Therapy Improves Functioning in Frail Older People: A Systematic Review.* J Am Geriatr Soc, 2017. **65**(8): p. 1863-1869.
30. Ekdahl, A., et al., *Frailty and comprehensive geriatric assessment organized as CGA-ward or CGA-consult for older adult patients in the acute care setting: A systematic review and meta-analysis*. Vol. 6. 2015.
31. Haun, M.W., et al., *Early palliative care for adults with advanced cancer.* Cochrane Database Syst Rev, 2017. **6**: p. CD011129.
32. Kavalieratos, D., et al., *Association between palliative care and patient and caregiver outcomes: a systematic review and meta-analysis.* Jama, 2016. **316**(20): p. 2104-2114.
33. McAlister, F.A., et al., *Multidisciplinary strategies for the management of heart failure patients at high risk for admission: a systematic review of randomized trials.* J Am Coll Cardiol, 2004. **44**(4): p. 810-9.
34. Phillips, C.O., et al., *Comprehensive discharge planning with postdischarge support for older patients with congestive heart failure: a meta-analysis.* JAMA, 2004. **291**(11): p. 1358-67.
35. Fox, M.T., et al., *Effectiveness of acute geriatric unit care using acute care for elders components: A systematic review and meta‐analysis.* Journal of the American Geriatrics Society, 2012. **60**(12): p. 2237-2245.
36. Kassianos, A.P., et al., *The impact of specialized palliative care on cancer patients' health-related quality of life: a systematic review and meta-analysis.* Supportive Care in Cancer, 2018. **26**(1): p. 61-79.
37. Cui, X., et al., *Collaborative care intervention for patients with chronic heart failure: A systematic review and meta-analysis.* Medicine, 2019. **98**(13).
38. Fulton, J.J., et al., *Integrated outpatient palliative care for patients with advanced cancer: a systematic review and meta-analysis.* Palliative medicine, 2019. **33**(2): p. 123-134.
39. Organization, W.H., *Integrated care for older people: guidelines on community-level interventions to manage declines in intrinsic capacity.* 2017: Geneva.
40. Gore, P.G., et al., *New horizons in the compression of functional decline.* Age Ageing, 2018. **47**(6): p. 764-768.
41. Sezgin D, O.C.R., Liew A, O' Donovan M, Salem MA, Kennelly S, Carriazo AM, Samaniego LL, Arnal C, Rodriguez-Acuna R, Inzitari M, Hammar T, Hendry A on behalf of work package 7 partners, *Intermediate care interventions for older adults*. 2019, Health Programme of the European Union, NHS Lanarkshire.
42. Kodner, D.L. and C. Spreeuwenberg, *Integrated care: meaning, logic, applications, and implications–a discussion paper.* International journal of integrated care, 2002. **2**.
43. Comas-Herrera, A., et al., *COVID-19: Implications for the Support of People with Social Care Needs in England.* Journal of Aging & Social Policy, 2020. **32**(4-5): p. 365-372.
44. Walshe, C., et al., *How effective are volunteers at supporting people in their last year of life? A pragmatic randomised wait-list trial in palliative care (ELSA).* BMC medicine, 2016. **14**(1): p. 203.
45. Humphries, R., et al., *Social care for older people: home truths*. 2016: King's Fund.
46. Goodwin, N., et al., *Providing integrated care for older people with complex needs: lessons from seven international case studies*. 2014: King's Fund London.
47. Robinson, L. and G.O.f.S. Foresight, *Present and future configuration of health and social care services to enhance robustness in older age*. 2015, London.
48. Colombo, F., et al., *Help Wanted? Providing and Paying for Long-Term Care* OECD Health Policy Studies. 2011, Paris: OECD Publishing.

For peer review only

49. Tiberini, R., K. Turner, and H. Talbot-Rice, *Rehabilitation in Palliative Care*, in *Textbook of Palliative Care*. 2018, Springer, Cham. p. 1-29.
50. Nicholson, C., et al., *What are the main palliative care symptoms and concerns of older people with multimorbidity? A comparative cross-sectional study using routinely collected Phase of Illness, Australian Modified Karnofsky Performance Scale and Integrated Palliative Care Outcome Scale data.* Ann Palliat Med, 2018. **7**(Suppl 3): p. S164-75.
51. Gomes, B. and I.J. Higginson, *Factors influencing death at home in terminally ill patients with cancer: systematic review.* Bmj, 2006. **332**(7540): p. 515-521.
52. Pollock, K., *Is home always the best and preferred place of death?* Bmj, 2015. **351**: p. h4855.
53. Davies, J.M., et al., *Socioeconomic position and use of healthcare in the last year of life: A systematic review and meta-analysis.* PLoS medicine, 2019. **16**(4): p. e1002782.
54. May, P., et al., *Economics of palliative care for hospitalized adults with serious illness: a meta-analysis.* JAMA internal medicine, 2018. **178**(6): p. 820-829.
55. Sleeman, K.E., et al., *The escalating global burden of serious health-related suffering: projections to 2060 by world regions, age groups, and health conditions.* Lancet Glob Health, 2019. **7**(7): p. e883-e892.
56. Glasziou, P.P., et al., *Intervention synthesis: a missing link between a systematic review and practical treatment (s).* PLoS medicine, 2014. **11**(8): p. e1001690.
57. Applegate, W.B., et al., *A randomized, controlled trial of a geriatric assessment unit in a community rehabilitation hospital.* New England Journal of Medicine, 1990. **322**(22): p. 1572-1578.
58. Asplund, K., et al., *Geriatric-based versus general wards for older acute medical patients: a randomized comparison of outcomes and use of resources.* Journal of the American Geriatrics Society, 2000. **48**(11): p. 1381-1388.
59. Austin, J., et al., *Randomised controlled trial of cardiac rehabilitation in elderly patients with heart failure.* European Journal of Heart Failure, 2005. **7**(3): p. 411-417.
60. Barnes, D.E., et al., *Acute care for elders units produced shorter hospital stays at lower cost while maintaining patients' functional status.* Health Affairs, 2012. **31**(6): p. 1227-1236.
61. Blue, L., et al., *Randomised controlled trial of specialist nurse intervention in heart failure.* BMJ, 2001. **323**(7315): p. 715-718.
62. Burton, E., et al., *Effectiveness of a lifestyle exercise program for older people receiving a restorative home care service: a pragmatic randomized controlled trial.* Clinical interventions in aging, 2013. **8**: p. 1591.
63. Capomolla, S., et al., *Cost/utility ratio in chronic heart failure: comparison between heart failure management program delivered by day-hospital and usual care.* Journal of the American College of Cardiology, 2002. **40**(7): p. 1259-1266.
64. Chang, B.-H., et al., *A relaxation response randomized trial on patients with chronic heart failure.* Journal of Cardiopulmonary Rehabilitation and Prevention, 2005. **25**(3): p. 149-157.
65. Clark, M.M., et al., *Randomized controlled trial of maintaining quality of life during radiotherapy for advanced cancer.* Cancer, 2013. **119**(4): p. 880-887.
66. Clemson, L., et al., *The effectiveness of a community-based program for reducing the incidence of falls in the elderly: A randomized trial.* Journal of the American Geriatrics Society, 2004. **52**(9): p. 1487-1494.
67. Clemson, L., et al., *Integration of balance and strength training into daily life activity to reduce rate of falls in older people (the LiFE study): randomised parallel trial.* Bmj, 2012. **345**: p. e4547.
68. Cline, C., et al., *Cost effective management programme for heart failure reduces hospitalisation.* Heart, 1998. **80**(5): p. 442-446.
69. Close, J., et al., *Prevention of falls in the elderly trial (PROFET): a randomised controlled trial.* The Lancet, 1999. **353**(9147): p. 93-97.
70. Collard, A.F., S.S. Bachman, and D.F. Beatrice, *Acute care delivery for the geriatric patient: an innovative approach.* QRB. Quality review bulletin, 1985. **11**(6): p. 180-185.
71. Counsell, S.R., et al., *Geriatric care management for low-income seniors: a randomized controlled trial.* Jama, 2007. **298**(22): p. 2623-2633.

For peer review only

72. Covinsky, K.E., et al., *Do acute care for elders units increase hospital costs? A cost analysis using the hospital perspective.* Journal of the American Geriatrics Society, 1997. **45**(6): p. 729-734.
73. de Lusignan, S., et al., *Compliance and effectiveness of 1 year's home telemonitoring. The report of a pilot study of patients with chronic heart failure.* European journal of heart failure, 2001. **3**(6): p. 723-730.
74. Doughty, R.N., et al., *Randomized, controlled trial of integrated heart failure management. The Auckland Heart Failure Management Study.* European Heart Journal, 2002. **23**(2): p. 139-146.
75. Dunbar, S.B., et al., *Randomized clinical trial of an integrated self-care intervention for persons with heart failure and diabetes: quality of life and physical functioning outcomes.* Journal of cardiac failure, 2015. **21**(9): p. 719-729.
76. Ekman, I., et al., *Feasibility of a nurse-monitored, outpatient-care programme for elderly patients with moderate-to-severe, chronic heart failure.* European Heart Journal, 1998. **19**(8): p. 1254-1260.
77. Fretwell, M.D., et al., *The senior care study: A controlled trial of a consultative/unit-based geriatric assessment program in acute care.* Journal of the American Geriatrics Society, 1990. **38**(10): p. 1073-1081.
78. Gary, R.A., et al., *Combined exercise and cognitive behavioral therapy improves outcomes in patients with heart failure.* Journal of psychosomatic research, 2010. **69**(2): p. 119-131.
79. Gitlin, L.N., et al., *A randomized trial of a multicomponent home intervention to reduce functional difficulties in older adults.* Journal of the American Geriatrics Society, 2006. **54**(5): p. 809-816.
80. Goldberg, L.R., et al., *Randomized trial of a daily electronic home monitoring system in patients with advanced heart failure: the Weight Monitoring in Heart Failure (WHARF) trial.* American heart journal, 2003. **146**(4): p. 705-712.
81. Harrison, M.B., et al., *Quality of life of individuals with heart failure: a randomized trial of the effectiveness of two models of hospital-to-home transition.* Medical care, 2002: p. 271-282.
82. Jaarsma, T., et al., *Effects of education and support on self-care and resource utilization in patients with heart failure.* European heart journal, 1999. **20**(9): p. 673-682.
83. Jerant, A.F., R. Azari, and T.S. Nesbitt, *Reducing the Cost of Frequent Hospital Admissions for Congestive Heart Failure: A Randomized Trial of a Home Telecare Intervention.* Medical Care, 2001. **39**(11): p. 1234-1245.
84. Kasper, E.K., et al., *A randomized trial of the efficacy of multidisciplinary care in heart failure outpatients at high risk of hospital readmission.* Journal of the American College of Cardiology, 2002. **39**(3): p. 471-480.
85. Krumholz, H.M., et al., *Randomized trial of an education and support intervention to preventreadmission of patients with heart failure.* Journal of the American College of Cardiology, 2002. **39**(1): p. 83-89.
86. Lang, C.C., et al., *A randomised controlled trial of a facilitated home-based rehabilitation intervention in patients with heart failure with preserved ejection fraction and their caregivers: the REACH-HFpEF Pilot Study.* BMJ open, 2018. **8**(4): p. e019649.
87. Laramee, A.S., et al., *Case Management in a Heterogeneous Congestive Heart Failure Population: A Randomized Controlled Trial.* JAMA Internal Medicine, 2003. **163**(7): p. 809-817.
88. Ledwidge, M., et al., *Is multidisciplinary care of heart failure cost-beneficial when combined with optimal medical care?* European Journal of Heart Failure, 2003. **5**(3): p. 381-389.
89. Luskin, F., et al., *A controlled pilot study of stress management training of elderly patients with congestive heart failure.* Preventive cardiology, 2002. **5**(4): p. 168-174.
90. Markle-Reid, M., et al., *The effects and costs of a multifactorial and interdisciplinary team approach to falls prevention for older home care clients 'at risk'for falling: a randomized controlled trial.* Canadian Journal on Aging/La Revue canadienne du vieillissement, 2010. **29**(1): p. 139-161.

For peer review only

91. McVey, L.J., et al., *Effect of a geriatric consultation team on functional status of elderly hospitalized patients: a randomized, controlled clinical trial.* Annals of internal medicine, 1989. **110**(1): p. 79-84.
92. Naylor, M., et al., *Comprehensive discharge planning for the hospitalized elderly: a randomized clinical trial.* Annals of internal Medicine, 1994. **120**(12): p. 999-1006.
93. Naylor, M.D., et al., *Comprehensive Discharge Planning and Home Follow-up of Hospitalized EldersA Randomized Clinical Trial.* JAMA, 1999. **281**(7): p. 613-620.
94. Northouse, L.L., et al., *Randomized clinical trial of a family intervention for prostate cancer patients and their spouses.* Cancer: Interdisciplinary International Journal of the American Cancer Society, 2007. **110**(12): p. 2809-2818.
95. Pugh, L.C., et al., *Case management for elderly persons with heart failure: the quality of life and cost outcomes.* MedSurg Nursing, 2001. **10**(2): p. 71.
96. Rainville, E.C., *Impact of pharmacist interventions on hospital readmissions for heart failure.* American Journal of Health-System Pharmacy, 1999. **56**(13): p. 1339-1342.
97. Rich, M.W., et al., *A Multidisciplinary Intervention to Prevent the Readmission of Elderly Patients with Congestive Heart Failure.* New England Journal of Medicine, 1995. **333**(18): p. 1190-1195.
98. Rich, M.W., et al., *Prevention of readmission in elderly patients with congestive heart failure.* Journal of General Internal Medicine, 1993. **8**(11): p. 585-590.
99. Riegel, B., et al., *Effect of a Standardized Nurse Case-Management Telephone Intervention on Resource Use in Patients With Chronic Heart Failure.* JAMA Internal Medicine, 2002. **162**(6): p. 705-712.
100. Rubenstein, L.Z., et al., *Effectiveness of a geriatric evaluation unit: a randomized clinical trial.* New England Journal of Medicine, 1984. **311**(26): p. 1664-1670.
101. Rubin, C.D., et al., *A randomized, controlled trial of outpatient geriatric evaluation and management in a large public hospital.* Journal of the American Geriatrics Society, 1993. **41**(10): p. 1023-1028.
102. Saltvedt, I., et al., *Randomised trial of in-hospital geriatric intervention: impact on function and morale.* Gerontology, 2006. **52**(4): p. 223-230.
103. Serxner, S., M. Miyaji, and J. Jeffords, *Congestive heart failure disease management study: a patient education intervention.* Congestive Heart Failure, 1998. **4**: p. 23-28.
104. Sherwood, A., et al., *Effects of coping skills training on quality of life, disease biomarkers, and clinical outcomes in patients with heart failure: a randomized clinical trial.* Circulation: Heart Failure, 2017. **10**(1): p. e003410.
105. Stewart, S., S. Pearson, and J.D. Horowitz, *Effects of a Home-Based Intervention Among Patients With Congestive Heart Failure Discharged From Acute Hospital Care.* JAMA Internal Medicine, 1998. **158**(10): p. 1067-1072.
106. Stewart, M., et al., *The impact of a geriatrics evaluation and management unit compared to standard care in a community teaching hospital.* Maryland medical journal (Baltimore, Md.: 1985), 1999. **48**(2): p. 62-67.
107. Strömberg, A., et al., *Nurse-led heart failure clinics improve survival and self-care behaviour in patients with heart failure: Results from a prospective, randomised trial.* European Heart Journal, 2003. **24**(11): p. 1014-1023.
108. Thomas, D.R., R. Brahan, and B.P. Haywood, *Inpatient community-based geriatric assessment reduces subsequent mortality.* Journal of the American Geriatrics Society, 1993. **41**(2): p. 101-104.
109. Trochu, J., S. Baleynaud, and G. Mialet, *Efficacy of a multidisciplinary management of chronic heart failure patients: one year results of a multicentre randomized trial in French medical practice.* Eur Heart J, 2004.
110. Tsuyuki, R.T., et al., *A multicenter disease management program for hospitalized patients with heart failure.* Journal of Cardiac Failure, 2004. **10**(6): p. 473-80.
111. Varma, S., et al., *Pharmaceutical care of patients with congestive heart failure: interventions and outcomes.* Pharmacotherapy: The Journal of Human Pharmacology and Drug Therapy, 1999. **19**(7): p. 860-869.

For peer review only - http://bmjopen.bmj.com/site/about/guidelines.xhtml

112. Vidán, M.T., et al., *An intervention integrated into daily clinical practice reduces the incidence of delirium during hospitalization in elderly patients.* Journal of the American Geriatrics Society, 2009. **57**(11): p. 2029-2036.
113. Wang, T.-C., et al., *Effects of a supportive educational nursing care programme on fatigue and quality of life in patients with heart failure: a randomised controlled trial.* European Journal of Cardiovascular Nursing, 2016. **15**(2): p. 157-167.
114. Yu, D.S., D.T. Lee, and J. Woo, *Improving health-related quality of life of patients with chronic heart failure: effects of relaxation therapy.* Journal of advanced nursing, 2010. **66**(2): p. 392-403.
115. Zelada, M.A., R. Salinas, and J.J. Baztán, *Reduction of functional deterioration during hospitalization in an acute geriatric unit.* Archives of gerontology and geriatrics, 2009. **48**(1): p. 35-39.
116. Bakitas, M., et al., *Effects of a palliative care intervention on clinical outcomes in patients with advanced cancer: the Project ENABLE II randomized controlled trial.* Jama, 2009. **302**(7): p. 741-749.
117. Bakitas, M.A., et al., *Early versus delayed initiation of concurrent palliative oncology care: patient outcomes in the ENABLE III randomized controlled trial.* Journal of Clinical Oncology, 2015. **33**(13): p. 1438.
118. Brännström, M. and K. Boman, *Effects of person-centred and integrated chronic heart failure and palliative home care. PREFER: a randomized controlled study.* European journal of heart failure, 2014. **16**(10): p. 1142-1151.
119. Edmonds, P., et al., *Palliative care for people severely affected by multiple sclerosis: evaluation of a novel palliative care service.* Multiple Sclerosis Journal, 2010. **16**(5): p. 627-636.
120. Given, B., et al. *Pain and fatigue management: results of a nursing randomized clinical trial.* in *Oncology nursing forum*. 2002.
121. Higginson, I.J., et al., *An integrated palliative and respiratory care service for patients with advanced disease and refractory breathlessness: a randomised controlled trial.* Lancet Respiratory Medicine, 2014. **2**(12): p. 979-987.
122. Jordhøy, M.S., et al., *Quality of life in palliative cancer care: results from a cluster randomized trial.* Journal of Clinical Oncology, 2001. **19**(18): p. 3884-3894.
123. Lowther, K., et al., *Nurse-led palliative care for HIV-positive patients taking antiretroviral therapy in Kenya: a randomised controlled trial.* The lancet HIV, 2015. **2**(8): p. e328-e334.
124. Maltoni, M., et al., *Systematic versus on-demand early palliative care: results from a multicentre, randomised clinical trial.* European Journal of Cancer, 2016. **65**: p. 61-68.
125. Ozcelik, H., et al., *Examining the effect of the case management model on patient results in the palliative care of patients with cancer.* American Journal of Hospice and Palliative Medicine®, 2014. **31**(6): p. 655-664.
126. Rogers, J.G., et al., *Palliative care in heart failure: the PAL-HF randomized, controlled clinical trial.* Journal of the American College of Cardiology, 2017. **70**(3): p. 331-341.
127. Rummans, T.A., et al., *Impacting quality of life for patients with advanced cancer with a structured multidisciplinary intervention: a randomized controlled trial.* Journal of Clinical Oncology, 2006. **24**(4): p. 635-642.
128. Sidebottom, A.C., et al., *Inpatient palliative care for patients with acute heart failure: outcomes from a randomized trial.* Journal of palliative medicine, 2015. **18**(2): p. 134-142.
129. Steel, J.L., et al., *W eb-based collaborative care intervention to manage cancer-related symptoms in the palliative care setting.* Cancer, 2016. **122**(8): p. 1270-1282.
130. Tattersall, M., et al., *Early contact with palliative care services: A randomised trial in patients with newly detected incurable metastatic cancer.* 2014.
131. Temel, J.S., et al., *Early palliative care for patients with metastatic non–small-cell lung cancer.* New England Journal of Medicine, 2010. **363**(8): p. 733-742.
132. Temel, J.S., et al., *Effects of early integrated palliative care in patients with lung and GI cancer: a randomized clinical trial.* Journal of Clinical Oncology, 2017. **35**(8): p. 834.

For peer review only

133. Wong, F.K.Y., et al., *Effects of a transitional palliative care model on patients with end-stage heart failure: a randomised controlled trial.* Heart, 2016. **102**(14): p. 1100-1108.

134. Zimmermann, C., et al., *Early palliative care for patients with advanced cancer: a cluster-randomised controlled trial.* The Lancet, 2014. **383**(9930): p. 1721-1730.

For peer review only

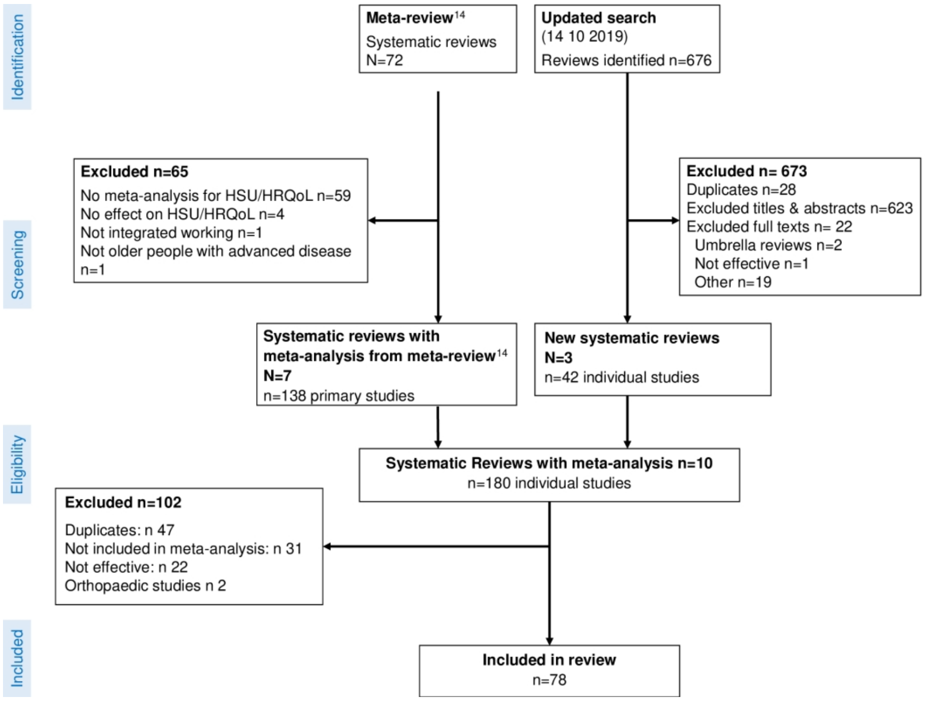

Figure 1. PRISMA flowchart for selection of primary studie

275x190mm (200 x 200 DPI)

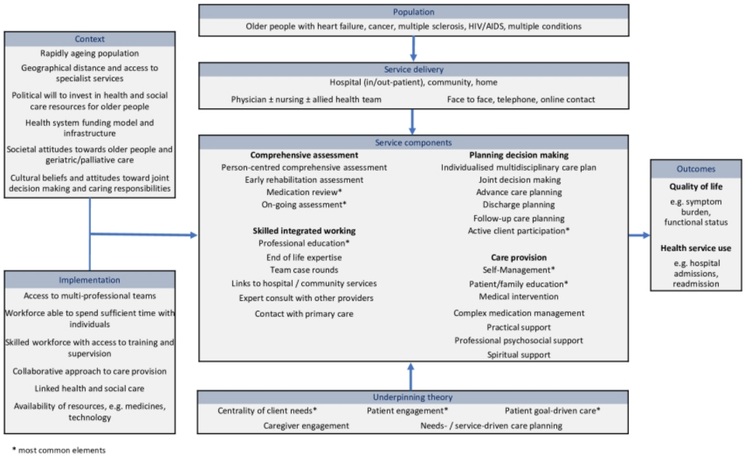

Figure 2. Common components logic model: Key elements of effective service delivery models for older people with advanced progressive conditions

338x190mm (200 x 200 DPI)

Supplementary material 1. Eligibility criteria for WHO 'Rapid review of service delivery models for older people at the end of life to maximise quality of life.'

	**Inclusion**		**Exclusion**
A	Participants at the end of life or living with advanced disease	Where information is available patients described as being in the last 1-2 years of life, or with advanced disease defined as advanced or metastatic cancer; chronic respiratory disease GOLD stage III-IV / grade C-D; heart failure New York Heart Association stage III or IV; progressive neurological disease; and frailty (excluding pre-frail)	Participants not described as being at the end of life or do not have advanced disease
B	Participants are older people	Where information is available at least 50% of the population must be greater than 60 years old or mean age greater than 60 years old	Where the information is available less than 50% of participants are older than 60 years old or mean age greater than 60 years old
C	Intervention must be a service delivery model aiming to improve quality of life	Service model must be an overarching model of health care provision with multiple components and interacting elements	Intervention is a single component intervention or focussing on post death intervention.
D	Outcome must be focussed on quality of life, function and dignity or cost-effectiveness	Outcomes of quality of life, function and dignity to include wellbeing, resilience, personal satisfaction, empowerment, goal attainment, autonomy, independence, mastery, adaptation, symptoms including pain, breathlessness, anxiety, depression, constipation, falls, any measure of psychosocial or spiritual distress, patient and caregiver satisfaction<br>Outcome of cost effectiveness	Outcome not focussed on quality of life, function or dignity
E	Design must be a review	Review must have searched at least 2 sources, one of which must be an electronic database	Non-review level paper eg primary intervention
F	Review may include controlled or non-controlled trials	Review can include trials that are randomised (cluster, parallel, single-stage or cross-over design), non-randomised trials, controlled before-after studies, interrupted time series studies and repeated measures studies. Control group can include usual care, attention control, active control or no control	Review focussing on opinion piece, case studies, case series or descriptive studies

For peer review only

Supplementary material 2. Search Strategy for Medline

	Population EoL /advanced disease	Intervention e.g. hospital	Outcome
MESH terms	Exp Terminally ill /<br>Exp Terminal care/<br>Palliative Care/<br>Frailty/	Exp Patient admission/<br>Exp Patient readmission/<br>Geriatric nursing/<br>Primary nursing/<br>Hospice and palliative care nursing/<br>Exp Nursing services/<br>Symptom Assessment/<br>Geriatric Assessment/<br>Needs assessment/<br>Hospital volunteers/<br>Nursing process/<br>Exp Patient care planning/<br>Exp Progressive patient care/<br>Exp Caregivers/<br>Exp Home care services/<br>Exp Hospice care/<br>Exp Patient Care Team<br>Exp Continuity of Patient Care/	Exp Quality of life/<br>Exp Pain/<br>Exp Pain management/<br>Exp Dyspnea/<br>Exp Anxiety/<br>Exp Anxiety disorders/<br>Depression/<br>Exp Depressive disorder/<br>Personal satisfaction/<br>Exp Activities of daily living/<br>Constipation/<br>Accidental Falls/<br>Exp Mental health/<br>Exp Social isolation/<br>Exp Social support/<br>Exp Patient satisfaction/<br>Exp Budgets/<br>Exp Costs and cost analysis/<br>Economics/<br>Exp Economics, hospital/<br>Exp Economics, medical/<br>Economics, nursing/<br>Exp Fees and charges/<br>Exp Resource allocation/<br>Value of life/
Key terms	EoL.tw<br>End?of?life.tw<br>Dying.tw<br>Palliative.tw<br>Last adj4 life.tw<br>Hospice.tw<br>Life limit*tw<br>Advanced disease*tw<br>Palliative treatment.tw<br>Palliative medicine.tw<br>Terminal care.tw<br>Terminally ill.tw<br>End-of-life care.tw<br>Hospice care.tw<br>Palliation.tw.<br>Palliative care$.tw.<br>Multi*morbidity.tw<br>Co*morbidity.tw<br>((Frail old*) AND (people OR adult* OR person*)).ti,ab<br>Frail*.tw<br>Frail elder*.ti,ab<br>Frailty syndrome*.ti,ab<br>Advanced illness.tw	Integrated care.tw<br>Model adj4 care.tw<br>Multi?disciplin*tw<br>Multi?disciplinary team.tw<br>Volunteer* tw<br>Volunt*tw<br>Hospital adj3 home.tw<br>Comprehensive assess*tw<br>Holistic assess*<br>(special$ adj2 palliat$).tw.<br>Nurse-led.tw<br>Co?ordination adj3 care.tw<br>Care plans.tw<br>Care?giver*.tw<br>Person?centr*.tw<br>Self?manage*.tw<br>Community health worker*.tw<br>Service delivery.tw<br>Community?based.tw<br>Home visit*.tw<br>Case management.tw<br>Care management.tw	Good death.tw<br>Symptom*.tw<br>Concern*.tw<br>Attainment<br>Dignity.tw<br>Empowerment.tw<br>Transition*.tw<br>Pain.tw<br>Dyspn?ea.tw<br>Breathless*.tw<br>Anxiety.tw<br>Anxious.tw<br>Depress*.tw<br>Quality of life.tw<br>Qol.tw<br>(quality adj2 life).tw.<br>Distress.tw<br>Wellbeing.tw<br>ADL*.tw<br>Activities of daily living.tw<br>Constipat*.tw<br>Fall*.tw<br>Mobil*.tw<br>Symptom management.tw.<br>Psychosocial.tw.<br>(psycho adj social).tw.<br>Psychological distress.tw.<br>Enablement.tw<br>Mastery.tw<br>Resilience.tw<br>Stress.tw<br>Financ*tw<br>(Cost* or economic*).ti<br>(Cost* adj2 (effective* or utilit* or benefit* or minimi*)).ab.

For peer review only

			Economic model*.tw<br>(Budget* or fee* or financ* or pricing or price* or resource* allocat* or (value adj2 (monetary or money))).ti,ab
BOLEAN TERMS	OR	OR	OR
	AND		
LIMIT	((Overview*.ti OR Review.ti OR Synthesis.ti OR Summary.ti OR Cochrane.ti OR Analysis.ti) AND (reviews.ti OR meta-analyses.ti OR articles.ti OR umbrella.ti)) OR ‘‘umbrella review’’.ti,ab OR (meta-review.ti.ab ORMetareview.ti,ab) OR ((overview*.ti OR Reviews.ti) AND (systematic.ti OR Cochrane.ti)) OR (reviews.ti,ab and (meta.ti,ab OR Published.ti,ab OR Quality.ti,ab OR Included.ti,ab OR summar*.ti,ab)) OR (‘‘cochrane reviews’’.ti,ab) OR (evidence.ti AND (reviews.ti OR meta-analyses.ti))		

t

For peer review only

Supplementary material 3. Data extraction framework: CATWOE elements

Service Delivery Model area (CATWOE)	Model elements / processes	Operational definition
**C(customers):** Target population and case mix	Population needs assessment	Population targeted by the intervention
	Setting	Where intervention is delivered: *Hospital in-patients/ hospital out-patients/ home/ primary care/community / mixed settings*
**A(actors):** Workforce including professions, level of skill and training	Multi-disciplinary team care	Multi-disciplinery team comprises ≥3 disciplines
	Rehabilitation expertise or training	Recognised rehabilitation expertise or training (i.e. Allied Health Professionals)
	End of life expertise or training	Recognised Palliative Care expertise or training (i.e. Palliative Care physician/or specialist Palliative Clinical Nurse Specialist or explicit statement of palliative and end of life care training)
	Professional education	Persons delivering intervention are educated and trained to nationally recognised standards and regulations.
**T(transformation process):** Service model elements / components	Comprehensive Assessment	I.e. comprehensive assessment- across multiple domains including physical/psychological/social/spiritual
	Case Management	Each person's overall care assigned to a team or individual
	Collaborative Working	Working across disciplines to plan services and deliver care to meet needs
	Route(s) of access, source and criteria for referral	How are participants recruited or eligible to participate?
	Professional psychosocial support	Explicit psychological support offered as component of intervention (i.e. psychologist/counsellor/Social Worker)
	Contact established with primary care or attending physician	Does interventionist contact physician as part of intervention?
	Patient and family education	Education for patient &/or family caregiver
	Individual multi-disciplinary care plan	Explicit description of multi-disciplinary team care plan
	Medical intervention	Medical intervention part of intervention, not alongside
	Team case rounds	Intervention includes team meetings, not usual care meetings
	Practical support	Any practical help i.e. in home, with medication boxes, equipment

For peer review only

	Early rehabilitation assessment	Intervention includes rehabilitation early in course of persons integrated geriatric care or integrated palliative care
	Systematic risk screening	Risk screening part of intervention delivery
	Discharge planning	Discharge planning a component of intervention
	Bereavement support	As stated
	Spiritual support	As stated
	Advance care planning	Formal advanced care planning
	Emergency response plan	Emergency only or plan for acute changes, i.e. worsening symptoms
	Self-management	As stated
	Medication review	Review part of intervention
	Complexity/medication management	Ongoing management of medication during intervention
**T:** Mode of delivery	Physician home visits	As part of intervention
	Physician available around the clock	As stated
	Interaction between professional and patient	Face to face/telephone/online or combination
	Access to dedicated inpatient beds	As stated
	Around the clock home visits available	As stated
	Ongoing assessment	Intervention includes multiple points of or ongoing assessment
**T:** Operational tools & guidance to support practice, e.g. assessment or decision support tools	Chart in the home	Diary, manual, medical/nursing record
	Medical review: standardized admission assessment	Explicitly reports standardised assessment is used
	Patient-centred care: standardized comprehensive assessment	Evidence of use comprehensive assessment tools or guidance relating to patient needs
**W (worldview):** Methods of integrated working	Joint provision across health and social care	Care involves explicit links between health and social care (in residential/nursing home care or home) providers
	Linkage with hospital	Intervention involves links with hospital services or is provided by hospital
	Linkage between community services	Intervention involves links with community services
	Expert consultation with other providers	Intervention involves consultation with other multi-disciplinary teams.
	Linkage with residential hospice	As stated
	One contact number	As stated- but reports contact number given

For peer review only

	Ongoing / continuous care	Ongoing care following the intervention made explicit
**W**: Conceptual model	Patient directed goal driven care	Patient involved in setting goals
	Centrality of patient* needs	Intervention focuses on individual patients needs
	Care mandate -service driven or needs- and benefits-driven	Service driven intervention = same intervention delivered to everyone with customisation and tailoring<br>Needs driven = patients' needs determine delivery of individualised intervention components
	Joint decision-making	Patient involved in decision making during delivery of intervention
	Active patient participation	Involves client or patient actively participating in behaviours
	Patient engagement	Intervention targets patient
	Caregiver engagement	Intervention targets caregiver
**W:** Provider Sector(s)	Visiting volunteer sectors	Volunteers explicitly involved in delivery of intervention
**O (Owners)**	Location	Country name
	World Bank status	High, Upper middle, Low Middle, Low
	Health service funding	State, private for profit, private non-profit, voluntary sector, other
**E (environmental constraints):** Country setting, sites, human resources,	Enabling environment	Policy, infrastructure, workforce training, rural or urban settings
	Resource requirements -human resources	Human resources- name all professionals involved in intervention delivery

*for consistency, we decided to use term 'patient' while acknowledging that in some settings the term client may be interchangeable or preferred.

For peer review only

Supplementary material 4. Included study characteristics

Author / year	WHO Region	Country	WBC Income status	Population	Setting	Sample Size
**Integrated Geriatric Care**						
Applegate 1990[57]	Americas	USA	High	Acutely ill older people	Hospital in-pts	156
Asplund 2000[58]	Europe	Sweden	High	Acutely ill older people	Hospital in-pts	190
Austin 2005[59]	Europe	UK	High	People with heart failure	Hospital out-pts	200
Barnes 2012[60]	Americas	USA	High	Acutely ill older people	Hospital in-pts	1632
Blue 2001[61]	Europe	UK	High	People with heart failure	Home	165
Burton 2013[62]	W. Pacific	Australia	High	Older people	Home	80
Capomollo 2002[63]	Europe	Italy	High	People with heart failure	Hospital out-pts	234
Chang 2005 [64]	Americas	USA	High	People with heart failure	Hospital out-pts	95
Clark 2013[65]	Americas	USA	High	People with advanced cancer	Home	129
Clemson 2004[66]	W. Pacific	Australia	High	Older people	Community	310
Clemson 2012[67]	W. Pacific	Australia	High	Older people	Home	317
Cline 1998[68]	Europe	Sweden	High	People with heart failure	Mixed settings (IP, OP)	190
Close 1999[69]	Europe	UK	High	Acutely ill older people	Mixed settings (ER, H)	397
Collard 1985[70]	Americas	USA	High	Acutely ill older people	Hospital in-pts	720
Counsell 2007[71]	Americas	USA	High	Older people	Home	951
Covinsky 1997[72]	Americas	USA	High	Acutely ill older people	Hospital in-pts	650
de Lusignan 2001[73]	Europe	UK	High	People with heart failure	Mixed settings (OP, H)	20
Doughty 2002[74]	W. Pacific	New Zealand	High	People with heart failure	Mixed settings (IP, H, OP)	197
Dunbar 2015[75]	Americas	USA	High	People with heart failure	Mixed settings (IP, H, OP)	134
Ekman 1998[76]	Europe	Sweden	High	People with heart failure	Hospital out-pts	158
Fretwell 1990[77]	Americas	USA	High	Acutely ill older people	Mixed settings (IP, OP)	436
Gary 2010[78]	Americas	USA	High	People with heart failure	Home	74
Gitlin 2006[79]	Americas	USA	High	Older people	Home	319
Goldberg 2003[80]	Americas	USA	High	People with heart failure	Home	282
Harrison 2002[81]	Americas	Canada	High	People with heart failure	Mixed settings (IP, H)	192
Jaarsma 1999[82]	Europe	Netherlands	High	People with heart failure	Mixed settings (IP, H)	179
Jerant 2001[83]	Americas	USA	High	People with heart failure	Home	37
Kasper 2002[84]	Americas	USA	High	People with heart failure	Mixed settings (IP, OP)	200
Krumholz 2002[85]	Americas	USA	High	People with heart failure	Mixed settings	88
Lang 2018[86]	Europe	UK	High	People with heart failure	Home	50
Laramee 2003[87]	Americas	USA	High	People with heart failure	Mixed settings (IP, H)	287
Ledwidge 2003[88]	Europe	Ireland	High	People with heart failure	Mixed settings (IP, H)	98
Luskin 2002[89]	Americas	USA	High	People with heart failure	Hospital out-pts	33

For peer review only - http://bmjopen.bmj.com/site/about/guidelines.xhtml

Markle-Reid 2010[90]	Americas	Canada	High	Older people	Home	109
McVey 1989[91]	Americas	USA	High	Acutely ill older people	Hospital in-pts	178
Naylor 1994[92]	Americas	USA	High	Acutely ill older people	Mixed settings (IP, H)	276
Naylor 1999[93]	Americas	USA	High	Acutely ill older people	Mixed settings (IP, OP, H)	363
Northouse 2007[94]	Americas	USA	High	People with cancer	Home	263
Pugh 2001[95]	Americas	USA	High	People with heart failure	Mixed settings	58
Rainville 1999[96]	Americas	USA	High	People with heart failure	Mixed settings (OP, H)	34
Rich 1995[97]	Americas	USA	High	People with heart failure	Mixed settings (IP, OP)	282
Rich 1993[98]	Americas	USA	High	People with heart failure	Mixed setting (IP, OP)	98
Riegel 2002[99]	Americas	USA	High	People with heart failure	Home	358
Rubenstein 1984[100]	Americas	USA	High	Acutely ill older people	Hospital in-pts	123
Rubin 1993[101]	Americas	USA	High	Acutely ill older people	Community	200
Saltvedt 2006[102]	Europe	Norway	High	Acutely ill older people	Hospital in-pts	254
Serxner 1998[103]	Americas	USA	High	People with heart failure	Hospital out-pts	109
Sherwood 2017[104]	Americas	USA	High	People with heart failure	Community	180
Stewart S 1998[105]	W. Pacific	Australia	High	People with heart failure	Mixed settings (IP, H)	97
Stewart M 1999 [106]	Americas	USA	High	Acutely ill older people	Hospital in-pts	61
Stromberg 2003[107]	Europe	Sweden	High	People with heart failure	Hospital out-pts	106
Thomas 1993[108]	Americas	USA	High	Acutely ill older people	Hospital in-pts	120
Trochu 2004[109]	Europe	France	High	People with heart failure	Mixed settings (OP, H)	202
Tsuyuki 2004[110]	Americas	Canada	High	People with heart failure	Mixed settings (IP, OP, H)	276
Varma 1999[111]	Europe	UK	High	People with heart failure	Mixed settings (OP, H)	83
Vidan 2009[112]	Europe	Spain	High	Acutely ill older people	Hospital in-pts	542
Wang 2016[113]	SE Asia	Taiwan	High	People with heart failure	Hospital out-pts	92
Yu 2010[114]	SE Asia	Hong Kong, China	High	People with heart failure	Hospital out-pts	158
Zelada 2009[115]	Americas	Peru	High middle	Acutely ill older people	Hospital in-pts	143
**Integrated Palliative Care**						
Bakitas 2009[116]	Americas	USA	High	People with advanced cancer	Home	322
Bakitas 2015[117]	Americas	USA	High	People with advanced cancer	Mixed settings (OP, H)	207
Brannstrom 2014[118]	Europe	Sweden	High	People with heart failure	Mixed settings (OP, H)	72
Edmonds 2010[119]	Europe	UK	High	People with multiple sclerosis	Mixed settings (OP, H)	52
Given 2002[120]	Americas	USA	High	People with cancer	Home	113
Higginson 2014[121]	Europe	UK	High	People with advanced diseases	Mixed settings (OP, H)	105
Jordhoy 2001[122]	Europe	Norway	High	People with advanced cancer	Mixed settings (IP, OP, H)	434
Lowther 2015[123]	Africa	Kenya	Low middle	People with HIV	Hospital out-pts	120
Maltoni 2016[124]	Europe	Italy	High	People with advanced cancer	Hospital out-pts	207
Ozcelik 2014[125]	Europe	Turkey	High middle	People with advanced cancer	Mixed settings (IP, OP, H)	44
Rogers 2017[126]	Americas	USA	High	People with heart failure	Mixed settings (IP, OP, H)	150
Rummans 2006[127]	Americas	USA	High	People with advanced cancer	Hospital out-pts	115

For peer review only

Sidebottom 2015[128]	Americas	USA	High	People with heart failure	Hospital in-pts	232
Steel 2016[129]	Americas	USA	High	People with advanced cancer	Mixed settings (OP, H)	261
Tattersall 2014[130]	W. Pacific	Australia	High	People with advanced cancer	Hospital out-pts	120
Temel 2010[131]	Americas	USA	High	People with advanced cancer	Hospital out-pts	151
Temel 2017[132]	Americas	US	High	People with advanced cancer	Hospital out-pts	350
Wong 2016[133]	SE Asia	China	High	People with heart failure	Home	84
Zimmermann 2014[134]	Americas	Canada	High	People with advanced cancer	Mixed settings (OP, H)	461

Key: IP =In-patients; OP = out-patients, ER =Emergency Room, H= home; WBC= World Bank Classification

For peer review only

Supplementary material 5 Assessment of Methodological Quality in Included Reviews (AMSTAR)

First Author, Year	A priori design provided	Duplicate study selection/ data extraction	Systematic literature search performed	Status of publication used as an inclusion criterion	List of studies (included and excluded) provided	Characteristics of the included studies provided	Scientific quality of included studies assessed and documented	Scientific quality of included studies used appropriately in formulating conclusions?	Were the methods used to combine the findings of the studies appropriate?	Was the likelihood of publication bias assessed?	Was the conflict of interest included?	Total
**Cui 2019**	No	Yes	Yes	Yes	No	No	Yes	No	Yes	Yes	Yes	**7**
**De Coninck, 2017**	Yes	No	Yes	No	Yes	Yes	Yes	Yes	Yes	No	No	**7**
**Ekdahl 2015**	No	Yes	Yes	No	No	Yes	Yes	Yes	Yes	No	No	**6**
**Fox 2012**	Yes	Yes	Yes	No	Yes	Yes	Yes	Yes	Yes	Yes	Yes	**9**
**Fulton 2019**	Yes	Yes	Yes	No	Yes	Yes	Yes	Yes	Yes	No	Yes	**9**
**Haun 2017**	Yes	Yes	Yes	Yes	Yes	Yes	Yes	Yes	Yes	Yes	No	**9**
**Kavalieratos 2016**	Yes	Yes	Yes	No	No	Yes	Yes	Yes	Yes	Yes	No	**8**
**McAlister 2004**	Yes	Yes	Yes	No	No	No	No	No	Yes	No	No	**4**
**Phillips 2004**	Yes	Yes	Yes	No	No	Yes	Yes	Yes	Yes	Yes	No	**8**
**Zimmermann 2008**	Yes	Yes	Yes	No	Yes	Yes	Yes	Yes	Yes	No	Yes	**9**
**Kassianos 2018**	Yes	Yes	Yes	Yes	No	Yes	Yes	Yes	Yes	Yes	Yes	**10**
											**Median**	**8**

Supplementary material 6. Risk of Bias Table for included studies

Author/ Year	Randomisation Sequence generation	Allocation Concealment	Blinding of participants and personnel	Blinding of outcome assessments	Incomplete outcome assessment	Slective reporting	Other bias
**Integrated Geriatric Care**							
Applegate 1990	Low	High	High	High	Low	Low	Low
Asplund 2000	Low	Low	High	High	High	Unclear	Low
Austin 2005	Low	Low	Low	Unclear	Low	Low	Unclear
Barnes 2012	Low	High	High	High	Unclear	Low	Low
Blue 2001	Unclear	Unclear	High	Unclear	Unclear	High	Unclear
Burton 2013	Low	Low	High	High	Low	Low	Unclear
Capamello 2002	High	High	High	Unclear	Unclear	High	High
Chang 2005	Low	Unclear	Unclear	Unclear	Low	Low	Unclear
Clark M 2013	High	High	High	High	High	Unclear	Unclear
Clemson 2004	High	Low	High	Low	High	Low	Unclear
Clemson 2012	Low	Low	High	Low	Low	Low	Unclear
Cline 1998	Low	Low	High	Unclear	Low	Low	Unclear
Close 1999	Low	Low	High	High	Low	High	Low
Collard 1985	Low	High	Low	Unclear	High	High	Low
Counsell 2007	Low	Low	Low	Low	Unclear	Unclear	Low
Covinsky 1997	Low	Low	Unclear	Unclear	Low	Low	Low
de Lusigan 2001	Low	Unclear	High	Unclear	Low	Low	Unclear
Doughty 2002	Low	Low	High	Unclear	Low	Low	Unclear
Dunbar 2015	Low	Low	Low	Unclear	Low	Low	Unclear
Ekman 1998	Low	Unclear	High	Unclear	Unclear	High	Unclear
Fretwell 1990	Unclear	Unclear	Unclear	Unclear	Low	Low	Low
Gary 2010	Unclear	Unclear	High	Low	Low	Low	Unclear
Gitlin 2006	Low	Low	High	Low	Low	Low	Low
Goldberg 2003	Low	Low	High	High	Low	Low	Unclear
Harrison 2002	Low	Low	Low	Unclear	Low	Low	Unclear
Jaarsma 1999	Low	Unclear	Unclear	Low	Low	Low	Unclear
Jerant 2001	Low	Unclear	High	High	Low	High	Unclear
Kasper 2002	Low	Unclear	Unclear	Unclear	Low	Low	Unclear
Krumholz 2002	Unclear	Unclear	High	Low	Low	High	Unclear
Lang 2018	Low	Unclear	High	Low	Low	Low	Unclear
Laramee 2003	Unclear	Unclear	High	High	Unclear	High	Unclear
Ledwidge 2003	High	High	High	Unclear	Low	High	Unclear
Luskin 2002	High	Unclear	High	Unclear	Low	Low	Unclear
Markle-Reid 2010	Low	Low	High	Low	Low	Low	Unclear
McVey 1989	Low	Low	High	Low	High	High	Unclear
Naylor 1994	Low	Low	Low	Unclear	Low	High	Unclear
Naylor 1999	Low	Low	Low	Low	Low	High	Unclear
Northouse 2007	Low	Low	High	High	Low	Low	Low
Pugh 2001	High	High	High	High	High	High	High
Rainville 1999	High	High	Unclear	High	Low	High	High
Rich 1995	Low	Low	Low	Unclear	Low	Low	Unclear

Rich 1993	High	High	High	High	Low	High	High
Riegel 2002	High	High	High	Unclear	Unclear	High	High
Rubenstein 1984	Unclear	Unclear	High	Unclear	High	Low	Unclear
Rubin 1993	Low	Low	High	Low	High	Low	Unclear
Saltvedt 2006	Low	Low	High	Low	High	Low	Low
Serxner 1998	Unclear	Unclear	High	High	High	High	High
Sherwood 2017	Low	Low	High	Unclear	Low	Low	Unclear
Stewart S 1998	Unclear	Unclear	High	High	Low	High	High
Stewart M 1999 (Fox)	High	Unclear	Unclear	Unclear	Unclear	Unclear	Low
Stromberg 2003	Low	Low	High	Low	Low	High	Unclear
Thomas 1993	Low	Low	High	High	High	Low	Low
Trochu 2004	Unclear	Unclear	Unclear	Unclear	Unclear	Unclear	Unclear
Tsuyuki 2004	Low	Low	High	Unclear	Low	Unclear	Unclear
Varma 1999	Unclear	Unclear	Unclear	High	High	High	High
Vidan 2009	High	Unclear	Unclear	Unclear	High	Unclear	Low
Wang 2016	Unclear	Unclear	High	Low	High	Unclear	Unclear
Yu 2010	High	High	High	High	High	Unclear	Unclear
Zeleda 2009	High	Unclear	Unclear	Unclear	High	Unclear	Low
**Integrated Palliative Care**							
Bakitas 2009	Low	High	High	Unclear	Low	Low	Low
Bakitas 2015	Low	Unclear	High	Low	Low	Low	High
Brannstrom 2014	Unclear	Low	High	Low	High	High	High
Edmonds 2010	Low	Low	High	Low	Unclear	Low	Unclear
Given 2002	Low	Unclear	High	High	Unclear	High	Low
Higginson 2014	Low	Low	High	High	Low	Low	Low
Jordhoy 2001	Unclear	Unclear	High	High	Low	Low	Low
Lowther 2015	Low	Low	High	High	Low	Low	Low
Maltoni 2016	Low	Low	High	Unclear	Low	Low	Low
Ozcelik 2014	High	High	High	High	Low	High	Unclear
Rogers 2017	Low	Unclear	High	High	Low	Low	Unclear
Rummans 2006	Low	Low	High	Low	Low	Low	Low
Sidebottom 2015	Unclear	Unclear	High	High	Low	Low	Low
Steel 2016	Low	Low	High	High	Low	High	High
Tattersall 2014	Low	Low	High	Unclear	High	Unclear	Unclear
Temel 2010	Low	High	High	Unclear	Low	Low	Low
Temel 2017	Low	Low	High	Low	Low	Unclear	Unclear
Wong 2016	Low	Low	Unclear	High	Low	High	Low
Zimmerman 2014	Low	High	Low	High	Low	Low	Low

For peer review only - http://bmjopen.bmj.com/site/about/guidelines.xhtml

PRISMA 2009 Checklist

Section/topic	#	Checklist item	Reported on page #
**TITLE**			
Title	1	Identify the report as a systematic review, meta-analysis, or both.	1
**ABSTRACT**			
Structured summary	2	Provide a structured summary including, as applicable: background; objectives; data sources; study eligibility criteria, participants, and interventions; study appraisal and synthesis methods; results; limitations; conclusions and implications of key findings; systematic review registration number.	2
**INTRODUCTION**			
Rationale	3	Describe the rationale for the review in the context of what is already known.	3 & 4
Objectives	4	Provide an explicit statement of questions being addressed with reference to participants, interventions, comparisons, outcomes, and study design (PICOS).	4 & 5
**METHODS**			
Protocol and registration	5	Indicate if a review protocol exists, if and where it can be accessed (e.g., Web address), and, if available, provide registration information including registration number.	5
Eligibility criteria	6	Specify study characteristics (e.g., PICOS, length of follow-up) and report characteristics (e.g., years considered, language, publication status) used as criteria for eligibility, giving rationale.	5
Information sources	7	Describe all information sources (e.g., databases with dates of coverage, contact with study authors to identify additional studies) in the search and date last searched.	5 Supplementary material 1 & 2
Search	8	Present full electronic search strategy for at least one database, including any limits used, such that it could be repeated.	Supplementary material 2
Study selection	9	State the process for selecting studies (i.e., screening, eligibility, included in systematic review, and, if applicable, included in the meta-analysis).	5
Data collection process	10	Describe method of data extraction from reports (e.g., piloted forms, independently, in duplicate) and any processes for obtaining and confirming data from investigators.	5 & 6
Data items	11	List and define all variables for which data were sought (e.g., PICOS, funding sources) and any assumptions and simplifications made.	5 & 6 Supplementary material 3
Risk of bias in individual studies	12	Describe methods used for assessing risk of bias of individual studies (including specification of whether this was done at the study or outcome level), and how this information is to be used in any data synthesis.	6 Supplementary

For peer review only - http://bmjopen.bmj.com/site/about/guidelines.xhtml

PRISMA 2009 Checklist

			material 5 & 6
Summary measures	13	State the principal summary measures (e.g., risk ratio, difference in means).	n/a
Synthesis of results	14	Describe the methods of handling data and combining results of studies, if done, including measures of consistency (e.g., I^2) for each meta-analysis.	n/a

Section/topic	#	Checklist item	Reported on page #
Risk of bias across studies	15	Specify any assessment of risk of bias that may affect the cumulative evidence (e.g., publication bias, selective reporting within studies).	7
Additional analyses	16	Describe methods of additional analyses (e.g., sensitivity or subgroup analyses, meta-regression), if done, indicating which were pre-specified.	n/a
**RESULTS**			
Study selection	17	Give numbers of studies screened, assessed for eligibility, and included in the review, with reasons for exclusions at each stage, ideally with a flow diagram.	7<br>Figure 1
Study characteristics	18	For each study, present characteristics for which data were extracted (e.g., study size, PICOS, follow-up period) and provide the citations.	7<br>Table 1<br>Supplementary Material 4
Risk of bias within studies	19	Present data on risk of bias of each study and, if available, any outcome level assessment (see item 12).	8<br>Supplementary material 6
Results of individual studies	20	For all outcomes considered (benefits or harms), present, for each study: (a) simple summary data for each intervention group (b) effect estimates and confidence intervals, ideally with a forest plot.	n/a
Synthesis of results	21	Present results of each meta-analysis done, including confidence intervals and measures of consistency.	n/a
Risk of bias across studies	22	Present results of any assessment of risk of bias across studies (see Item 15).	8
Additional analysis	23	Give results of additional analyses, if done (e.g., sensitivity or subgroup analyses, meta-regression [see Item 16]).	n/a
**DISCUSSION**			
Summary of evidence	24	Summarize the main findings including the strength of evidence for each main outcome; consider their relevance to key groups (e.g., healthcare providers, users, and policy makers).	10

For peer review only - http://bmjopen.bmj.com/site/about/guidelines.xhtml

PRISMA 2009 Checklist

Limitations	25	Discuss limitations at study and outcome level (e.g., risk of bias), and at review-level (e.g., incomplete retrieval of identified research, reporting bias).	12
Conclusions	26	Provide a general interpretation of the results in the context of other evidence, and implications for future research.	13<br>Box 1
**FUNDING**			
Funding	27		14

From: Moher D, Liberati A, Tetzlaff J, Altman DG, The PRISMA Group (2009). Preferred Reporting Items for Systematic Reviews and Meta-Analyses: The PRISMA Statement. PLoS Med 6(6): e1000097. doi:10.1371/journal.pmed1000097

For more information, visit: **www.prisma-statement.org**.

For peer review only

For peer review only - http://bmjopen.bmj.com/site/about/guidelines.xhtml

BMJ Open

Common elements of service delivery models that optimise quality of life and health service use among older people with advanced progressive conditions: a tertiary systematic review

Journal:	*BMJ Open*
Manuscript ID	bmjopen-2020-048417.R1
Article Type:	Original research
Date Submitted by the Author:	14-Jun-2021

Complete List of Authors:	Bayly, Joanne; King's College London, Cicely Saunders Institute for Palliative Care, Policy and Rehabilitation; St Barnabas Hospice<br>Bone, Anna; King's College London, Cicely Saunders Institute for Palliative Care, Policy and Rehabilitation<br>Ellis-Smith, Clare; King's College London, Cicely Saunders Institute for Palliative Care, Policy and Rehabilitation<br>Tunnard, India; King's College London, Cicely Saunders Institute for Palliative Care, Policy and Rehabilitation<br>Yaqub, Shuja; King's College London, Cicely Saunders Institute for Palliative Care, Policy and Rehabilitation<br>Yi, Deokhee; King's College London, Cicely Saunders Institute for Palliative Care, Policy and Rehabilitation<br>Bashan Nkhoma, Kennedy; King's College London, Florence Nightingale Faculty of Nursing Midwifery and Palliative Care<br>Cook, Amelia; King's College London, Cicely Saunders Institute for Palliative Care, Policy and Rehabilitation<br>Combes, Sarah; King's College London, Florence Nightingale Faculty of Nursing, Midwifery & Palliative Care ; St Christopher's Hospice<br>Bajwah, Sabrina; King's College London, Cicely Saunders Institute for Palliative Care, Policy and Rehabilitation<br>Harding, Richard; King's College London, of Palliative Care, Policy and Rehabilitation<br>Nicholson, Caroline; University of Surrey Faculty of Health and Medical Sciences; St Christopher's Hospice<br>Normand, Charles; King's College London, Cicely Saunders Institute for Palliative Care, Policy and Rehabilitation; The University of Dublin Trinity College, Centre for Health Policy and Management<br>Ahuja, Shalini; King's College London Institute of Psychiatry Psychology and Neuroscience, Health Service and Population Research Department<br>Turrillas, Pamela; King's College London, Cicely Saunders Institute for Palliative Care, Policy and Rehabilitation<br>Kizawa, Yoshiyuki; Kobe University, Department of Palliative Medicine<br>Morita, Tatsuya; Seirei Mikatahara Hospital, Palliative and Supportive Care Division<br>Nishiyama, Nanako; Osaka Prefecture University, Graduate School of Comprehensive Rehabilitation<br>Tsuneto, Satoru; Kyoto University Hospital, Department of Human

Health Sciences, Department of Palliative Medicine
Ong, Paul; World Health Organization Centre for Health Development
Higginson, Irene; King's College London, Cicely Saunders Institute for Palliative Care, Policy and Rehabilitation
Evans, Catherine ; King's College London, Cicely Saunders Institute for Palliative Care, Policy and Rehabilitation; Sussex Community NHS Foundation Trust
Maddocks, Matthew; King's College London, Cicely Saunders Institute for Palliative Care, Policy and Rehabilitation

**Primary Subject Heading**:	Health services research
Secondary Subject Heading:	Palliative care
Keywords:	GERIATRIC MEDICINE, PALLIATIVE CARE, Organisation of health services < HEALTH SERVICES ADMINISTRATION & MANAGEMENT, Health policy < HEALTH SERVICES ADMINISTRATION & MANAGEMENT, Quality in health care < HEALTH SERVICES ADMINISTRATION & MANAGEMENT

SCHOLARONE™
Manuscripts

I, the Submitting Author has the right to grant and does grant on behalf of all authors of the Work (as defined in the below author licence), an exclusive licence and/or a non-exclusive licence for contributions from authors who are: i) UK Crown employees; ii) where BMJ has agreed a CC-BY licence shall apply, and/or iii) in accordance with the terms applicable for US Federal Government officers or employees acting as part of their official duties; on a worldwide, perpetual, irrevocable, royalty-free basis to BMJ Publishing Group Ltd ("BMJ") its licensees and where the relevant Journal is co-owned by BMJ to the co-owners of the Journal, to publish the Work in this journal and any other BMJ products and to exploit all rights, as set out in our licence.

The Submitting Author accepts and understands that any supply made under these terms is made by BMJ to the Submitting Author unless you are acting as an employee on behalf of your employer or a postgraduate student of an affiliated institution which is paying any applicable article publishing charge ("APC") for Open Access articles. Where the Submitting Author wishes to make the Work available on an Open Access basis (and intends to pay the relevant APC), the terms of reuse of such Open Access shall be governed by a Creative Commons licence – details of these licences and which Creative Commons licence will apply to this Work are set out in our licence referred to above.

Other than as permitted in any relevant BMJ Author's Self Archiving Policies, I confirm this Work has not been accepted for publication elsewhere, is not being considered for publication elsewhere and does not duplicate material already published. I confirm all authors consent to publication of this Work and authorise the granting of this licence.

For peer review only

Common elements of service delivery models that optimise quality of life and health service use among older people with advanced progressive conditions: a tertiary systematic review

Joanne Bayly[1,13], Anna E Bone[1], Clare Ellis-Smith[1], India Tunnard[1], Shuja Yaqub[1], Deokhee Yi[1], Kennedy B Nkhoma[1,] Amelia Cook[1,] Sarah Combes[3, 11], Sabrina Bajwah[1], Richard Harding[1], Caroline Nicholson[10,11], Charles Normand[1, 4], Shalini Ahuja[5], Pamela Turrillas[1], Yoshiyuki Kizawa[6], Tatsuya Morita[7], Nanako Nishiyama [8], Satoru Tsuneto[9], Paul Ong[12], Irene J Higginson[1], Catherine J Evans[1,2*] Matthew Maddocks[1*] (*joint senior authors)

Affiliations

1. King's College London, Cicely Saunders Institute of Palliative Care, Policy and Rehabilitation, London, SE5 9PJ, United Kingdom
2. Sussex Community NHS Foundation Trust, Brighton General Hospital, Elm Grove, BN2 3EW, United Kingdom
3. King's College London, Florence Nightingale Faculty of Nursing, Midwifery & Palliative Care, James Clerk Maxwell Building, 57 Waterloo Road, London, SE1 8WA, United Kingdom
4. Centre for Health Policy and Management, Trinity College, University of Dublin, Ireland
5. King's College London, Health Service and Population Research Department, Institute of Psychiatry, Psychology and Neuroscience, De Crespigny Park, London, SE5 8AF, United Kingdom
6. Kobe University, Department of Palliative Medicine, 7-5-2, Kusunokicho, Chuo-ku, Kobe, Hyogo, Japan, 650-0017
7. Seirei Mikatahara Hospital, Palliative and Supportive Care Division, 3453 Mikatahara, Kita, Hamamatsu, 433-8558, Japan
8. Osaka Prefecture University, Graduate School of Comprehensive Rehabilitation, 3-7-30, Habikino, Habikino-city, Osaka, Japan
9. Department of Human Health Sciences; Kyoto University Hospital, Department of Palliative Medicine, 53 Kawaharacho, Shogoin, Sakyo-ku, Kyoto 606-8507, Japan
10. University of Surrey, Faculty of Health and Medical Sciences, Kate Granger Building, Priestley Road, Surrey Research Park, Guildford, Surrey GU2 7XH
11. St Christopher's Hospice, 51-59 Lawrie Park Rd, London SE26 6DZ
12. World Health Organisation Centre for Health Development
13. St Barnabas Hospices, Worthing, West Sussex, UK

Correspondence: Dr Matthew Maddocks & Dr Catherine Evans

Email: matthew.maddocks@kcl.ac.uk Telephone: +44 (0)207 848 5242

For peer review only

Abstract

Introduction: Health and social care services worldwide need to support ageing populations to live well with advanced progressive conditions while adapting to functional decline and finitude. We aimed to identify and map common elements of effective geriatric and palliative care services and consider their scalability and generalisability to high, middle and low-income countries.

Methods: Tertiary systematic review (Cochrane Database of Systematic Reviews, CINAHL, Embase, January 2000-October 2019) of studies in geriatric or palliative care that demonstrated improved quality-of-life and/or health-service use outcomes among older people with advanced progressive conditions. Using frameworks for health system analysis, service elements were identified. We used a staged, iterative process to develop a 'common components' logic model and consulted experts in geriatric or palliative care from high, middle and low-income countries on its scalability.

Results: 78 studies (59 geriatric, 19 palliative) spanning all WHO regions were included. Data was available from 17,739 participants. Nearly half the studies recruited patients with heart failure (n=36) and one-third recruited patients with mixed diagnoses (n=26). Common service elements (≥80% of studies) included collaborative working, on-going assessment, active patient participation, patient/family education and patient self-management. Effective services incorporated patient engagement, patient goal-driven care, and the centrality of patient needs. Stakeholders (n=20) emphasised that wider implementation of such services would require access to skilled, multidisciplinary teams with sufficient resource to meet patients' needs. Identified barriers to scalability included the political and societal will to invest in and prioritise palliative and geriatric care for older people, alongside geographical and socioeconomic factors.

Conclusion: Our logic model combines elements of effective services to achieve optimal quality of life and health service use among older people with advanced progressive conditions. The model transcends current best practice in geriatric and palliative care and applies across the care continuum, from prevention of functional decline to end-of-life care.

Key words

Geriatrics, Palliative Care, Delivery of Health Care, Quality of Life, Systematic Reviews

For peer review only

Strengths and limitations of this study:

We draw on and synthesise a diverse evidence base of geriatric and palliative care for older people with progressive advanced conditions across the globe.

The review was conducted by a multidisciplinary and international group representing broad methodological expertise and perspectives.

Our common components logic model is a recombination of effective service elements. However, we were unable to assert how outcomes may be influenced by different combinations of components and their interactions.

Our stakeholder consultation identified significant barriers to scalability where country health budgets cannot meet the growing population need, and where multidisciplinary care is not available.

For peer review only

Introduction

Globally, more people are living into old age [1] with the largest proportional increase occurring in those 80 years and above [2, 3]. By 2050, 80% of older people will live in low and middle income countries (LMIC)[4]. The concomitant risks of multi-morbidity and/or frailty [5] mean more people experience a trajectory of prolonged and uncertain functional decline. Health and social care needs and their impact on physical functioning are more heterogeneous[1] in older populations, shaped by multiple interacting factors related to the individual and their environment. These population changes bring new societal challenges related to health and social care policy, spending, workforce and security, regardless of the developmental context.

The WHO Member States' commitment to achieve Universal Health Coverage (UHC) by 2030 provides an opportunity to plan health and social care delivery for the future. Palliative care has recently been included as an essential service that is fundamental to achieving UHC [6]. While prevention remains a priority across the health continuum, a shift in health systems is needed to balance disease-modifying interventions with services where improving quality of life is the main goal of care. In older people with advanced (incurable) and progressive diseases, health systems must align to support the dual priorities of living well while adapting to a gradual decline in function. Access to appropriate care and support is recognised as a basic human right [7], yet access varies according to socioeconomic and geographic variables [8, 9]. Budget constraints require maximum value from the resources

used to improve outcomes [10]. The importance of integrated working across services is consistently advocated in global guidance on health service provision for advanced disease[11] and older people [12].

Our previous meta-review outlined two service delivery models for older people towards the end of life; ‘integrated geriatric care’ and ‘integrated palliative care’ [12]. Both showed potential to improve quality of life and patterns of health service use, but with differing emphasis on either function or symptoms and concerns. Our findings underscored the imperative of access to services based on the likelihood of benefit and integration of care using comprehensive assessment, case management, and/or collaborative working [12]. However, use of systematic reviews as the unit of analysis prevented a detailed description of service model elements, and supressed the heterogeneity across the primary studies.

This review aimed to detail service delivery models that optimise quality of life and health services use for older people aged 60 years and over with advanced progressive health conditions. We defined 'advanced' to include disease stage, people described as in their last one or two years of life or people accessing a service typically used in advanced disease stage, such as nursing home or palliative care. Our objectives were to: i) identify and map common elements of effective service delivery models within primary studies; ii) outline the similarities and differences across models of geriatric care or palliative care and iii) consider the scalability of effective models, attending to implementation and economic requirements.

Method

Study Design

This review builds on our previous meta-review, where the methods are described in detail [12]. Here, we conducted a tertiary review of individual empirical studies ('primary studies') from the meta-review [12]. This was conducted in accordance with the Preferred Reporting Items for Systematic Reviews and Meta-Analysis [13]. We then used logic modelling [14] and a stakeholder consultation to support the analysis and interpretation [15] of findings. This study was registered on PROSPERO [CRD42020150252] prior to data extraction.

Patient and Public Involvement

Patients and members of the public were not involved in the design, conduct, reporting or dissemination of this research.

Search strategy

For the purposes of this tertiary review, in October 2019 we updated our original meta-review search to identify systematic reviews that included a meta-analysis that demonstrated overall effectiveness on at least one outcome for quality of life (including symptom burden and function) and/or health service use outcome. The systematic review eligibility criteria

For peer review only - http://bmjopen.bmj.com/site/about/guidelines.xhtml

and search terms are reported in Supplementary materials 1 and 2. From the eligible systematic reviews, we identified primary studies with evidence of effect on our selected outcomes of quality of life and/or health service use. Inclusion criteria for primary studies comprised: i) experimental study design; ii) contributed data to meta-analysis and iii) reported a point estimate of effect in the same direction as the meta-analysis. One reviewer (JB) evaluated all systematic reviews and primary studies for eligibility and a second (MM, AEB or CES) double-screened studies, with inconsistencies resolved by consensus. Duplicate primary studies were identified and removed.

Data extraction

Data on study population, outcomes and context were extracted. Service delivery models were classified as either integrated geriatric or palliative care. Data identification and extraction was informed by a framework for healthcare systems analysis, the Checklist CATWOE (customers, actors, transformation processes, world view, owner, environmental constraints) [16, 17]. For each CATWOE domain, (e.g. Customers, Actors), a list of service elements was identified. Service elements were categorised as present, absent, or unclear by two individuals (from JB, AEB, CES, SY, DY, NK, SB, CE, MM) and reviewed as a team. Identification of the elements for each CATWOE domain was , informed by the Template for Intervention Description and Replication (TIDieR) checklist for complex health service interventions [18] and prior studies on geriatric [19], integrated [20], transitional [21] and palliative care [22] Supplementary material 3 details the elements for each CATWOE domain.

Quality appraisal

The methodological quality of systematic reviews and primary studies was appraised using the AMSTAR tool [23] and Cochrane Risk of Bias Tool respectively [24]. We used the quality appraisal in the systematic reviews when the Cochrane Risk of Bias Tool was used, otherwise assessment was by two researchers (JB, IT). We did not exclude studies from analysis based on quality.

Development of logic model

We used a staged and iterative approach following Rohwer et al's guidance on logic models for complex health interventions [14] incorporating analysis of extracted data followed by a stakeholder consultation.

The frequency and proportion of service elements [16, 17] was summarised overall and separately for integrated geriatric and palliative care models. The proportion was calculated using studies where the element was categorised as present or absent. We mapped service elements present in ≥50% of integrated geriatric and/or palliative care studies by CATWOE domain to existing logic model templates [14] (see Supplementary Material 4). To compare

For peer review only

the presence of service elements between integrated geriatric and palliative care models we conducted chi squared tests (or Fisher's exact tests where counts were low).

We appraised the potential for the common components of effective interventions to be generalised and scalable, defined as the ability "to be expanded under real world conditions to reach a greater proportion of the eligible population while retaining effectiveness"[25].

We shared an interim logic model and consulted a purposive sample of healthcare researchers, clinical-academics and clinicians from high, middle and low-income countries with expertise in either geriatric or palliative care, hospital or community based. We used the Context and Implementation of Complex Interventions Framework (CICI) to develop a response form with free text open questions on the barriers and facilitators to providing the elements of care, for their respective country and healthcare settings [26]. The CICI framework domains informed the identification and collation of the narrative responses on the context and implementation considerations. We developed the logic model by synthesising the findings from the tertiary review and the stakeholder consultation, using an iterative process of team discussion and consensus [14].

Results

Study retrieval

Ten systematic reviews met eligibility, seven from the meta-review [27-33] and three from the updated search [34-36]. The reviews reported 180 potentially eligible studies, of which 47 were duplicates. Of the 133 remaining studies, 78 met eligibility (Figure 1).

Figure 1. *PRISMA flowchart for selection of primary studies*

To go here

Characteristics of included studies

Of the 78 included studies, 59 were categorised as integrated geriatric care and 19 as integrated palliative care (Table 1 and Supplementary material 5). All WHO regions were represented, though studies were predominantly from the North American region of the Americas (n=46) or Europe (n=22), with fewer from Western Pacific (n=6), South East Asia (n=3) and only a single study from Africa. Most studies were from high-income countries (n=75). The number of study participants ranged from 20 to 1632, with data available from 17,739 participants overall. Nearly half of all studies recruited patients with heart failure (n=36) and one-third recruited patients with mixed diagnoses (n=26). Palliative care studies most often recruited by cancer diagnosis (n=12). Study interventions were delivered across

For peer review only

multiple care settings (n=31), in participants' homes (n=15) or in hospital (outpatients n=14; inpatients n=12) (Table 1).

For peer review only

Table 1. Summary characteristics of included studies N=78

Variable		Frequency n (%)		
		All n=78	Geriatric n=59	Palliative n=19
**WHO region**	Americas	46 (59)	36 (61)	10 (53)
	Europe	22 (28)	16 (27)	6 (32)
	South East Asia	3 (4)	2 (3)	1 (5)
	West Pacific	6 (8)	5 (8)	1 (5)
	Africa	1 (1)	1 (2)	0
**Country income status**	High	75(96)	17 (29)	58
	Upper - middle	2 (3)	1 (2)	1
	Lower – middle	1 (1)	0	1
	Low	0	0	0
**Population by main diagnosis**	Heart failure	36 (46)	32 (54)	4 (21)
	No main diagnosis	23 (29)	23 (39)	0
	Cancer	14 (18)	2 (3)	12 (63)
	Single	4 (5)	1 (2)	3 (16)
	Mixed	10 (13)	1 (2)	9 (47)
	Heart failure + diabetes	1 (1)	1 (2)	0
	Heart failure + depression	1 (1)	1 (2)	0
	Multiple Sclerosis	1 (1)	0	1 (5)
	Mixed diagnosis (COPD, cancer, HF, ILD, MND)	1 (1)	0	1 (5)
	HIV infection	1 (1)	0	1 (5)
**Population by referral criteria**	People with heart failure	38 (49)	34 (58)	4 (21)
	People with acute episode of illness	17 (22)	17 (29)	0
	People with advanced cancer	13 (17)	2 (3)	11(58)
	Older people (varied age ranges)	6 (8)	6 (10)	0
	People with HIV	1 (1)	0	1 (5)
	People with multiple sclerosis	1 (1)	0	1 (5)
	Advanced mixed diagnoses	1 (1)	0	1 (5)
	People with cancer commencing chemotherapy	1 (1)	1 (2)	1 (5)
**Health organisation funding**	State funded health organisation	35 (45)	26 (44)	9 (47)
	For profit health organisation	37 (47)	28 (47)	9 (47)
	Non-profit health organisation	6 (8)	5 (8)	1 (5)
**Care Setting**	Mixed settings	29 (37)	20 (34)	9 (47)
	Hospital in-patients and home	6 (8)	6 (10)	0
	Hospital in-patients and out-patients	5 (6)	5 (8)	0
	Hospital out-patients and home	10 (13)	4 (7)	6 (32)
	Hospital in-patients, out-patients and home	7 (9)	4 (7)	3 (16)
	Hospital emergency room and home	1 (1)	1 (2)	0
	Home	16 (21)	13 (22)	3 (16)
	Hospital out-patients	15 (19)	9 (15)	6 (32)
	Hospital in-patients	13 (17)	12 (20)	1 (5)
	Community settings	3 (4)	3 (5)	0

COPD =Chronic Obstructive Pulmonary Disease, HF = Heart failure, MND = Motor neurone disease, ILD = Interstitial lung disease.

For peer review only

Quality appraisal

The ten systematic reviews were assessed as of moderate quality (Supplementary material 6). Primary studies were assessed as low to moderate risk of bias overall (Supplementary material 6). Where high risk of bias was found, this most frequently related to challenges of blinding participants and personnel leading to possible performance and detection bias. Risk of bias tended to be lower for palliative care compared to geriatric care studies (Supplementary material 7).

Service delivery elements

Services most frequently used collaborative working and case management to support integrated working between professionals (Table 2). Patient/family education was present in all studies. Other common elements, present in ≥80% of studies were on-going assessment, active patient participation, and evidence of patient engagement in their care. The least common elements overall were: bereavement support; 24-hour home visits or access to physicians; links to residential hospice facilities and joint provision of care across health and social care services. No studies reported delivering interventions in residential care/nursing homes or use of volunteers.

Comparing between integrated geriatric and palliative care, palliative care interventions had a higher frequency of end-of-life expertise and training, professional psychosocial support, spiritual support and physician home visits. In contrast geriatric care interventions more often featured early rehabilitation assessment and self-management, though the differences were not statistically significant (Table 2).

Service delivery agents

All interventions were delivered by qualified health care professionals (staff who have received nationally recognised and regulated training and education), most often working in multidisciplinary teams. Over 90% of studies involved trained medical and nursing clinicians and 59% involved members of the wider health care team, including physiotherapists, occupational therapists and social workers. Geriatric care services were delivered by physicians from geriatrics, cardiology and general practice, whereas palliative care services involved physicians from cardiology, neurology, respiratory medicine, oncology, psychiatry, primary care and palliative medicine. While involvement of physiotherapists was reported across all studies, fewer occupational therapists and dietitians were reported in those from palliative care. No studies reported the involvement of volunteers (Table 3).

For peer review only

Table 2. *Service delivery model elements N=78*

	All n (%)	Geriatric n (%)	Palliative n (%)	Sig^
Method of supporting integrated working				
Collaborative working	64 (82)	46 (78)	18 (95)	0.17*
Case management	61 (78)	46 (78)	15 (79)	1.00*
Comprehensive assessment	51 (65)	36 (68)	15 (79)	0.36
Actors-Workforce				
Professional Education	76 (100)	58 (100)	18 (100)	1.00
MDT Care	54 (72)	42 (73)	12 (71)	1.00*
Rehabilitation expertise training	34 (50)	27 (50)	7 (50)	1.00
End of life expertise training	18 (25)	1 (2)	17 (90)	<0.001*
Transformation- Service Model elements / components				
Patient family education	60 (100)	49 (100)	11 (100)	0.93
Medication review	51 (80)	40 (77)	11 (92)	0.43*
Self-management	48 (80)	41 (84)	7 (64)	0.21*
Systematic risk screening	47 (69)	37 (70)	10 (67)	1.00*
Contact with GP or attending doctor	46 (68)	33 (65)	13 (77)	0.37
Practical Support	41 (68)	34 (69)	7 (64)	0.73*
Medical intervention	52 (67)	39 (66)	13 (68)	0.85
Individualised MDT plan	40 (61)	29 (59)	11 (65)	0.69
Complex/medication management	37 (58)	30 (59)	7 (54)	0.75
Discharge planning	36 (52)	29 (55)	7 (44)	0.44
Professional psychosocial support	38 (51)	26 (44)	12 (80)	0.01
Team case rounds	25 (40)	18 (37)	7 (50)	0.37
Early rehab assessment	25 (38)	21 (40)	4 (29)	0.54
Advanced care planning	23 (30)	9 (16)	14 (78)	<0.001
Emergency response plan	15 (21)	12 (22)	3 (20)	1.00*
Spiritual support	13 (18)	2 (3)	11 (79)	<0.001*
Bereavement Support	4 (5)	0 (0)	4 (25)	0.002*
Transformation- Mode of Delivery				
On-going assessment	66 (87)	50 (86)	16 (89)	1.00*
Face to face & telephone	41 (53)	31 (53)	10 (53)	0.10
Face to face interaction	31 (40)	23 (39)	8 (42)	0.81
Access to inpatient beds	21 (30)	18 (32)	3 (21)	0.53*
Physician home visits	11 (15)	4 (7)	7 (37)	0.04*
24-hour Physician access	6 (10)	5 (11)	1 (7)	1.00*
Telephone only	5 (6)	4 (7)	1 (5)	1.00*
24-hour home visits	1 (1)	1 (2)	0 (0)	1.00*
Online only	1 (1)	1 (2)	0 (0)	0.10*
Transformation-Operational tools & guidance to support practice				
Standard comprehensive assessment	38 (59)	26 (55)	12 (71)	0.27
Worldview- Methods of Integrated Working				
Link to Hospital	57 (78)	41 (72)	16 (100)	0.02*
Expert consult with other providers	40 (58)	24 (45)	16 (100)	<0.001
Link between community services	31 (50)	22 (45)	9 (69)	0.12
Joint provision-health & social care	7 (10)	4 (7)	3 (20)	0.16*
Link to residential hospice	5 (7)	1 (2)	4 (27)	0.005*
Worldview-Conceptual Model				

For peer review only - http://bmjopen.bmj.com/site/about/guidelines.xhtml

Patient engagement	71 (99)	53 (98)	18 (100)	1.00*
Active patient participation	67 (99)	50 (98)	17 (100)	1.00*
Centrality of patient needs	64 (91)	46 (89)	18 (100)	0.33*
Patient goal driven care	56 (81)	40 (77)	16 (94)	0.16*
Ongoing / continuous care	46 (67)	33 (62)	13 (81)	0.16
Joint decision making	38 (69)	25 (61)	13 (93)	0.04*
Service driven care planning	38 (54)	34 (65)	4 (21)	0.001*
Needs and benefit-driven care planning	33 (46)	18 (35)	15 (79)	0.001
Caregiver engagement	32 (55)	22 (50)	10 (71)	0.16

* =Fisher's exact test. **^=**Sig for difference in presence of service delivery element between geriatric and palliative care studies

Table 3. *Service delivery model agents*

**Delivery Agent**	**All n (%)**	**Geriatric n (%)**	**Palliative n (%)**	**Sig^**
Physicians				
Geriatrician	14 (18)	14 (24)	0 (0)	0.02
Cardiologist	15 (19)	12 (20)	3 (16)	1.0
Palliative care physician	12 (15)	0 (0)	12 (63)	<0.001*
Neurologist	1 (1)	0 (0)	1 (5)	0.24*
Respiratory physician	1 (1)	0 (0)	1 (5)	0.24*
Oncologist	4 (5)	0 (0)	4 (21)	0.001*
Psychiatrist	2 (3)	0 (0)	2 (11)	0.06*
Physician	18 (23)	17 (29)	1 (5)	0.06*
Primary care doctor (GP)	5 (6)	4 (7)	1 (5)	0.55*
Physician assistant	2 (3)	2 (3)	0 (0)	0.43*
Nurses				
Nurse	24 (31)	22 (37)	2 (11)	0.28
Advanced nurse practitioner	13 (17)	8 (14)	5 (26)	0.17*
Specialist cardiac nurse	12 (15)	10 (17)	2 (11)	0.40*
Primary care nurse	9 (8)	8 (14)	1 (5)	0.30*
Specialist geriatric nurse	6(8)	6 (10)	0 (0)	0.18*
Case manager	5 (6)	3 (5)	2 (11)	0.35*
Specialist palliative nurse	4 (5)	1 (2)	3 (16)	0.43*
Specialist rehabilitation nurse	1 (1)	1 (2)	0 (0)	0.76*
Specialist HIV nurse	1 (1)	0 (0)	1 (5)	0.24*
Oncology nurse	1 (1)	0 (0)	1 (5)	0.24*
Allied Health Professionals				
Physiotherapist	23 (29)	17 (29)	6 (32)	0.85
Occupational Therapist	14 (18)	12 (20)	2 (11)	0.28*
Dietitian	16 (21)	14 (24)	2 (11)	0.18*
Psychologist	9 (15)	6 (10)	3 (16)	0.38*
Pharmacologist/pharmacist	7 (9)	7 (12)	0 (0)	0.13*
Chaplain	4 (5)	1 (2)	3 (16)	0.43*
Audiologist	1 (1)	1 (2)	0 (0)	0.76*
Speech and language therapist	1 (1)	1 (2)	0 (0)	0.76*
Social Care				
Social worker	21 (27)	17 (29)	4 (21)	0.51
Home care service manager	3 (4)	3 (5)	0 (0)	0.43*
Social assistant	4 (1)	3(5)	1(5)	0.68*
Other professionals				
Unspecified wider 'MDT'	11 (14)	9 (15)	2 (11)	0.47*
Exercise instructor	2 (3)	2 (3)	0 (0)	0.57*

* =Fisher's exact test. ^Sig = significance for difference in presence of service delivery element between geriatric and palliative = care studies.

For peer review only

Service outcomes including costs

Forty-five studies (58%) were included based on an effect on quality of life alone. Fifty-seven studies (73%) used a disease or population specific measure to quantify quality of life (Supplementary Material 5) and five studies (6%) employed the Euro-Qual-5D (EQ-5D). Thirty-three studies (42%) reported utilisation of acute care services (e.g. hospital admission, readmission after discharge) or community care services and 20 studies (26%) calculated costs of health services utilisation. Only a minority (n=12/15%) demonstrated an effect on both quality of life and health service use, all of which were geriatric care studies. No study used costs and EQ-5D to generate information required for health economic decision making (Table 4).

Table 4. *Number of studies reporting quality of life and health services use outcomes*

		Health service use			
		None	More than 1	1+ and costs	Sub total
Quality of life	None	0	6	15	21
	More than 1	40	7	5	52
	1+ and EQ-5D	4	0	0	5
	Sub total	45	13	20	78

Common components logic model

The interim logic model highlighted key elements present in the majority (<80%) of included studies.

Elements more common in integrated palliative care compared to geriatric care studies were; professional psychosocial support, advance care planning, care-giver engagement, joint decision making and expert consultation with other providers. Elements more common in geriatric care studies included a social worker or dietician as a delivery agent and care planning organised around the service, delivering the same intervention to all patients but with individual tailoring (Figure 2).

Elements more common in geriatric care studies included a social worker or dietician as a delivery agent and care planning organised around the service with the same intervention being delivered to all patients with individual tailoring (Figure 2).

For peer review only

Stakeholder perspectives on scalability

The context and implementation considerations identified from the stakeholder responses were incorporated into the logic model (Figure 2). Stakeholders (n=20) contributed views from high-income countries (n=12) (UK, Japan, Taiwan, Portugal, Chile) and LMICs (n=8) (Uganda, Malawi, South Africa, Ghana, Zimbabwe, China, India and Bangladesh) contributed views. They described increasing patient complexity with rapid population aging and the associated rise in multimorbidity, frailty and dementia. This raised particular challenges in LMICs where health services have historically focused on prevention and management of infectious diseases and where there has been a recent increased burden of non-communicable disease. Specialist services being based in major city hospitals were described as a barrier to providing care to rural populations. Recruiting, training and retaining skilled staff to work in rural areas and having a multidisciplinary team including allied health professionals and specialist doctors and nurses was considered infeasible for many rural areas.

Stakeholders from LMICs considered that overall health budgets were inadequate to meet the population need, and multidisciplinary care was considered unaffordable. The voluntary sector was often seen as important to augment publicly funded services. In some contexts, continuity of care is impeded when individually funded services compete for resources rather than collaborate. There are challenges to multidisciplinary working in systems where health workers receive payment directly from patients, as this was considered a financial disincentive to making referrals for expert consultation. Social deprivation was cited as an important barrier to accessing care, especially in health systems with out-of-pocket expenses or private insurance.

Stakeholders described how cultural norms influence care provision. Death denying attitudes in some cultures influence uptake of palliative care services. Some countries have limited recognition or respect for the specialities of palliative care and geriatric care. The role of the family and the health system to provide care was identified to vary across countries influenced by cultural beliefs such as filial piety, gender-related norms and changing intergenerational family structures. Acknowledging faith and religion were identified as factors supporting the delivery of individualised care aligned with spiritual needs in hospice and nursing homes.

Increasing education levels and internet access were identified as factors that are changing patient and family participation in joint decision-making. Finally, stakeholders recognised an increasing political will to invest in services for older people supported by a growing public and research agenda and established regulatory frameworks. However, this did not always equate to increases in funding. A lack of policies and clinical governance for specialist

For peer review only - http://bmjopen.bmj.com/site/about/guidelines.xhtml

palliative and geriatric care was reported as a problem, like legal restrictions on opiate prescribing limiting effective medication management of pain.

For peer review only

Figure 2. Common components logic model detailing effective service delivery models for older people with advanced progressive conditions
to go here

For peer review only

Discussion

We used rigorous methods to detail service delivery models that optimise quality of life and health service use outcomes among older people with advanced progressive conditions. Effective services commonly used collaborative working between professionals and specialities, comprehensive and on-going assessment, patient/family education, and active patient participation. Aligned to this, effective services consistently incorporated patient engagement, patient goal-driven care, and the centrality of patient needs in care delivery. Our logic model encompasses a breadth of elements that aim to 'protect' (discharge planning and falls prevention programmes), 'reactivate' (disease management, self-management and exercise programmes), 'compensate' (symptom management, support with capabilities for activities of daily living) and 'support' (enhancing social assets and provision of home care). Such practices may together support older people to maintain intrinsic capacity and functional ability [37] and to compress functional decline across the life course [38, 39]. This broad focus, together with consideration of social factors, extends health and social care beyond provision at the point of decline to meet the dual priorities of living well while adapting to a gradual functional decline [1].

This review has several strengths. It was conducted by a large multidisciplinary team with a range of methodological expertise and representation from many regions of the world. We synthesised a diverse literature with studies across different patient populations and needs across the trajectory of advanced disease. We used recognised frameworks to categorise studies, extract data and consult with stakeholders in order to develop a visual logic model applicable to different international settings. There are some limitations to consider. Data on study context is limited to country, country income status and the system for funding health care. Further information to support an evaluation of how interventions could be scaled and implemented would be valuable. Stakeholders identified limited applicability for some service elements, including multidisciplinary care, within lo-income countries where health budgets cannot meet the growing population need. Change beyond the health system, into education and health promotion, may be required to implement services that meet the challenge of rising incidence in diseases of ageing [40]. As in other reviews of complex interventions in this population [16], we were unable to discern the specific mechanisms of action that make each component effective. In part this was linked to our data extraction framework. For example, we did not extract data on how interventions provided care across care boundaries during care transitions, yet elements including on-going assessment and links between community services indicate this may have been occurring.

Our findings build on previous reviews. Bainbridge et al [22] found that 'linkages with hospital,' 'multiprofessional teams' and 'end of life care expertise and training' were critical to the delivery of end-of-life home care. In a review of integrated care for older people, Briggs

For peer review only

et al found that multidisciplinary teams, comprehensive assessment and case management were most frequently reported [20]. We show the importance of a capable workforce that works collaboratively across disciplinary boundaries, to provide comprehensive and ongoing multidimensional assessment. This model of care requires active patient engagement, participation and self-management with tailored care centred on the needs of individuals.[41] It allows for a shared understanding between the person(when able and/or the family) and the team providing their care, facilitating joint decision making that addresses their priorities in their context. [42].

We also provide new insights into the range of health and social care providers associated with effective services in this population. Services were frequently delivered by multidisciplinary teams of health and social care professionals with formal training in core skills of comprehensive assessment, communication and symptom management. These teams can support people to self-manage a progressive condition and help those close to them to provide care. Investment in training and education is required to achieve greater coverage and ensure the skills base keeps up with the needs of this growing population. Uncoupling skills from professional roles and working towards a generalist skills set may be most beneficial. However, this should ideally be accompanied by access to specialists for ongoing support and supervision. Volunteers may provide additional support that supplements or enhances usual health and social care provision [43, 44]. The absence of volunteers in studies probably reflects the fact that most were conducted in high-income countries.

Service elements that we consider relevant to the target population but not brought forward to our logic model include joint provision across health and social care and early rehabilitation assessment. Neglecting social care can have a considerable negative effect on quality of life for older people, their family and friends and lead to increased patient and carer morbidity and mortality [45].Integrated care should follow older people as they transition from acute to community care. [46] However workforce issues continue to influence the integration of health and social care delivery[47] as highlighted in our stakeholder consultation. Early rehabilitation assessment was detailed in only 40% and 19% of geriatric and palliative care studies respectively. Given that maintaining independence, normality and social participation are high priorities for older people towards the end of life [48], this was a surprising finding. It may relate to a focus on physical symptoms arising from advanced disease rather than functional needs, and the presumption that decline is an inevitability of disease progression [49]. The increasing prominence of rehabilitation in palliative care to challenge this misconception is therefore timely.[50, 51]

For peer review only

The logic model is a recombination of different services and we were unable to assert how effectiveness may be influenced by different combinations of components and their interactions. Consequently, the model remains untested as a whole [52]. However the model can inform health and social care policy and support the conceptual and organisational development of services. We recommend that the clinical and cost effectiveness of interventions, underpinned by our proposed model, should be tested in older people with multi-morbidity based on need, rather than diagnostic condition, over longer trajectories and across care boundaries. Implications for policy are presented in Box 1.

Box 1. Implications for Policy
• Configure services for the whole trajectory of chronic progressive conditions up until the end of life and move away from a focus on acute episodes of care<br>• Plan and deliver education to drive provision of a capable workforce. A broad range of professional education courses and training in core skills of geriatric and palliative care, including comprehensive assessment, communication and symptom management specific to individual need is required<br>• Incentivise interdisciplinary and collaborative working between professional disciplines and across health and social care settings, to optimise high-quality individualised service provision and care coordination. This integrated care, when aligned to need rather than diagnostic condition, will increase the reach and impact of services and promote equitable access<br>• Enable robust evaluation by embedding routine outcome measurement in health and social care settings. These should include measures of intrinsic capacity, functional ability, symptom experience and quality of life. Measures should capture the changes in health and social well-being that are associated with the provision of high quality individualised care across the care continuum from protect to support and end of life care

Conclusion

Our logic model brings together common elements of interventions found to optimise quality of life and health service use among older people with advanced progressive conditions. These included collaborative working between professionals and specialities, on-going assessment, active patient participation, patient/family education and patient self-management, whilst effective service delivery approaches consistently incorporated patient engagement, patient goal-driven care, and the centrality of patient needs.

For peer review only - http://bmjopen.bmj.com/site/about/guidelines.xhtml

These elements transcend best practices in geriatric care and palliative care to optimise patient outcomes across the continuum, from prevention of functional decline to end of lifecare. The logic model serves as a useful resource for health systems looking to strengthen their response to population aging. It can guide provision of health and social care that is aligned to the needs of this rapidly growing population. Such care should allow older people across the globe to live fully, with mimimal suffering, and to die with dignity.

Contributions:

JB, AEB, CES, RH, CNormand, SA, PT, YK, TM, NN, ST, PO, IJH, CJE, MM conceived and designed the study. JB, AEB, CES, IT, SY, DY, KBN, AC, SC, SB, CJE, MM extracted data. JB, AEB, CES, SY, AC, SC, CNicholson, CNormand, RH, KBN, CJE, MM analysed data. JB, AEB, AC, SC, CNicholson, PO, CJE, MM drafted the manuscript, All authors critically revised the draft and approved the final manuscript.

Acknowledgements:

We thank Olivia Dix for proofreading the manuscript and Hamid Benalia for help with the production of the figures.

Ethics Statement:

This study does not involve human participants

Competing Interests

Paul Ong reported that he was an employee of the funding sponsor, the World Health Organization, and was involved in the extraction, analysis, and interpretation of data. All other authors have no competing interests.

Funding

This research was funded by the World Health Organization [grant reference WKC-EOLC-K19002] and supported by The Dunhill Medical Trust [grant number RPGF1906\177] and the National Institute of Health Research Applied Research Collaboration South London (NIHR ARC South London NIHR200152) at King's College Hospital NHS Foundation Trust. AEB is supported by the Dunhill Medical Trust [number RPGF1906\177] and Cicely Saunders International. SC is funded by a Health Education England/NIHR Clinical Doctoral Research Fellowship (ICA-CDRF-2017-03-012) and CN by a Health Education England/NIHR Senior Clinical Lectureship (ICA-SCL-2018-04-ST2-001). IJH is an NIHR Senior Investigator Emeritus. CE is funded by a Health Education England/NIHR Senior Clinical Lectureship (ICA-SCL-2015-01-001) and MM is funded by an NIHR Career Development Fellowship (CDF-2017-10-009). This publication presents independent research funded by the National Institute for Health Research (NIHR). The views expressed in this publication are those of

For peer review only

the author(s) and not necessarily those of the NHS, NIHR or the Department of Health and Social Care.

Data sharing statement

Extracted data is available on request from the corresponding author.

For peer review only

References

1. WHO, *World Report on Aging and Health*. 2015, World Health Organisation.
2. Evans, C.J., et al., *Place and cause of death in centenarians: a population-based observational study in England, 2001 to 2010.* PLoS Med, 2014. **11**(6): p. e1001653.
3. Nations, U., *World population prospects: The 2015 revision, key findings and advance tables.* Rep No. ESA/P/WP. 241, 2015.
4. WHO. *Ageing and health*. [cited 2018 21/11/2018]; Available from: http://www.who.int/news-room/fact-sheets/detail/ageing-and-health.
5. Clegg, A., et al., *Frailty in elderly people.* The lancet, 2013. **381**(9868): p. 752-762.
6. World, H., Organisation. *Universal Health Coverage (UHC)*. 2020 [cited 2020 02 07 20]; Available from: https://www.who.int/news-room/fact-sheets/detail/universal-health-coverage-(uhc).
7. Ahmedzai, S.H., et al., *A new international framework for palliative care.* European Journal of Cancer, 2004. **40**(15): p. 2192-2200.
8. Walshe, C., et al., *Patterns of access to community palliative care services: a literature review.* Journal of pain and symptom management, 2009. **37**(5): p. 884-912.
9. Cohen, L.L., *Racial/ethnic disparities in hospice care: a systematic review.* Journal of palliative medicine, 2008. **11**(5): p. 763-768.
10. Porter, M.E., *What is value in health care.* N Engl J Med, 2010. **363**(26): p. 2477-2481.
11. WPCA and WHO, *Global Atlas of Palliative Care at the End of Life*. 2014, World Palliative Care Alliance: London.
12. Evans, C.J., et al., *Service Delivery Models to Maximize Quality of Life for Older People at the End of Life: A Rapid Review.* The Milbank Quarterly, 2019. **97**(1): p. 113-175.
13. Moher, D., et al., *Preferred reporting items for systematic review and meta-analysis protocols (PRISMA-P) 2015 statement.* Systematic reviews, 2015. **4**(1): p. 1.
14. Rohwer, A., et al., *Guidance on the use of logic models in health technology assessments of complex interventions.* International Journal of Technology Assessment in Health Care, 2016.
15. Popay, J., et al., *Guidance on the conduct of narrative synthesis in systematic reviews: A product from the ESRC Methods Programme*. 2006.
16. Brereton, L., et al., *What do we know about different models of providing palliative care? Findings from a systematic review of reviews.* Palliative medicine, 2017. **31**(9): p. 781-797.
17. Checkland, P. and C. Tsouvalis, *Reflecting on SSM: the link between root definitions and conceptual models.* Systems Research and Behavioral Science: The Official Journal of the International Federation for Systems Research, 1997. **14**(3): p. 153-168.
18. Hoffmann, T.C., et al., *Better reporting of interventions: template for intervention description and replication (TIDieR) checklist and guide.* Bmj, 2014. **348**: p. g1687.
19. Fox, M.T., et al., *Acute care for elders components of acute geriatric unit care: systematic descriptive review.* J Am Geriatr Soc, 2013. **61**(6): p. 939-46.
20. Briggs, A.M., et al., *Elements of integrated care approaches for older people: a review of reviews.* BMJ Open, 2018. **8**(4): p. e021194.
21. Naylor, M.D., et al., *Components of Comprehensive and Effective Transitional Care.* J Am Geriatr Soc, 2017. **65**(6): p. 1119-1125.
22. Bainbridge, D., H. Seow, and J. Sussman, *Common Components of Efficacious In-Home End-of-Life Care Programs: A Review of Systematic Reviews.* J Am Geriatr Soc, 2016. **64**(3): p. 632-9.
23. Shea, B.J., et al., *Development of AMSTAR: a measurement tool to assess the methodological quality of systematic reviews.* BMC medical research methodology, 2007. **7**(1): p. 10.
24. Higgins, J.P., et al., *The Cochrane Collaboration's tool for assessing risk of bias in randomised trials.* Bmj, 2011. **343**: p. d5928.
25. Milat, A.J., et al., *The concept of scalability: increasing the scale and potential adoption of health promotion interventions into policy and practice.* Health promotion international, 2013. **28**(3): p. 285-298.

For peer review only

26. Pfadenhauer, L.M., et al., *Making sense of complexity in context and implementation: the Context and Implementation of Complex Interventions (CICI) framework.* Implementation science, 2017. **12**(1): p. 21.
27. De Coninck, L., et al., *Home- and Community-Based Occupational Therapy Improves Functioning in Frail Older People: A Systematic Review.* J Am Geriatr Soc, 2017. **65**(8): p. 1863-1869.
28. Ekdahl, A., et al., *Frailty and comprehensive geriatric assessment organized as CGA-ward or CGA-consult for older adult patients in the acute care setting: A systematic review and meta-analysis*. Vol. 6. 2015.
29. Haun, M.W., et al., *Early palliative care for adults with advanced cancer.* Cochrane Database Syst Rev, 2017. **6**: p. CD011129.
30. Kavalieratos, D., et al., *Association between palliative care and patient and caregiver outcomes: a systematic review and meta-analysis.* Jama, 2016. **316**(20): p. 2104-2114.
31. McAlister, F.A., et al., *Multidisciplinary strategies for the management of heart failure patients at high risk for admission: a systematic review of randomized trials.* J Am Coll Cardiol, 2004. **44**(4): p. 810-9.
32. Phillips, C.O., et al., *Comprehensive discharge planning with postdischarge support for older patients with congestive heart failure: a meta-analysis.* JAMA, 2004. **291**(11): p. 1358-67.
33. Fox, M.T., et al., *Effectiveness of acute geriatric unit care using acute care for elders components: A systematic review and meta-analysis.* Journal of the American Geriatrics Society, 2012. **60**(12): p. 2237-2245.
34. Kassianos, A.P., et al., *The impact of specialized palliative care on cancer patients' health-related quality of life: a systematic review and meta-analysis.* Supportive Care in Cancer, 2018. **26**(1): p. 61-79.
35. Cui, X., et al., *Collaborative care intervention for patients with chronic heart failure: A systematic review and meta-analysis.* Medicine, 2019. **98**(13).
36. Fulton, J.J., et al., *Integrated outpatient palliative care for patients with advanced cancer: a systematic review and meta-analysis.* Palliative medicine, 2019. **33**(2): p. 123-134.
37. Sezgin D, O.C.R., Liew A, O' Donovan M, Salem MA, Kennelly S, Carriazo AM, Samaniego LL, Arnal C, Rodriguez-Acuna R, Inzitari M, Hammar T, Hendry A on behalf of work package 7 partners, *Intermediate care interventions for older adults*. 2019, Health Programme of the European Union, NHS Lanarkshire.
38. Gore, P.G., et al., *New horizons in the compression of functional decline.* Age Ageing, 2018. **47**(6): p. 764-768.
39. Organization, W.H., *Integrated care for older people: guidelines on community-level interventions to manage declines in intrinsic capacity.* 2017: Geneva.
40. Sleeman, K.E., et al., *The escalating global burden of serious health-related suffering: projections to 2060 by world regions, age groups, and health conditions.* Lancet Glob Health, 2019. **7**(7): p. e883-e892.
41. Evans, C.J., et al., *Community-based short-term integrated palliative and supportive care reduces symptom distress for older people with chronic noncancer conditions compared with usual care: a randomised controlled single-blind mixed method trial.* International Journal of Nursing Studies, 2021: p. 103978.
42. Kodner, D.L. and C. Spreeuwenberg, *Integrated care: meaning, logic, applications, and implications–a discussion paper.* International journal of integrated care, 2002. **2**.
43. Comas-Herrera, A., et al., *COVID-19: Implications for the Support of People with Social Care Needs in England.* Journal of Aging & Social Policy, 2020. **32**(4-5): p. 365-372.
44. Walshe, C., et al., *How effective are volunteers at supporting people in their last year of life? A pragmatic randomised wait-list trial in palliative care (ELSA).* BMC medicine, 2016. **14**(1): p. 203.
45. Humphries, R., et al., *Social care for older people: home truths*. 2016: King's Fund.
46. Robinson, L. and G.O.f.S. Foresight, *Present and future configuration of health and social care services to enhance robustness in older age*. 2015, London.

For peer review only - http://bmjopen.bmj.com/site/about/guidelines.xhtml

47. Colombo, F., et al., *Help Wanted? Providing and Paying for Long-Term Care* OECD Health Policy Studies. 2011, Paris: OECD Publishing.
48. Tiberini, R., K. Turner, and H. Talbot-Rice, *Rehabilitation in Palliative Care*, in *Textbook of Palliative Care*. 2018, Springer, Cham. p. 1-29.
49. Nicholson, C., et al., *What are the main palliative care symptoms and concerns of older people with multimorbidity? A comparative cross-sectional study using routinely collected Phase of Illness, Australian Modified Karnofsky Performance Scale and Integrated Palliative Care Outcome Scale data.* Ann Palliat Med, 2018. **7**(Suppl 3): p. S164-75.
50. Kitzman, D.W., et al., *Physical Rehabilitation for Older Patients Hospitalized for Heart Failure.* New England Journal of Medicine, 2021.
51. Nottelmann, L., et al., *Early, integrated palliative rehabilitation improves quality of life of patients with newly diagnosed advanced cancer: The Pal-Rehab randomized controlled trial.* Palliative Medicine, 2021: p. 02692163211015574.
52. Glasziou, P.P., et al., *Intervention synthesis: a missing link between a systematic review and practical treatment (s).* PLoS medicine, 2014. **11**(8): p. e1001690.

For peer review only

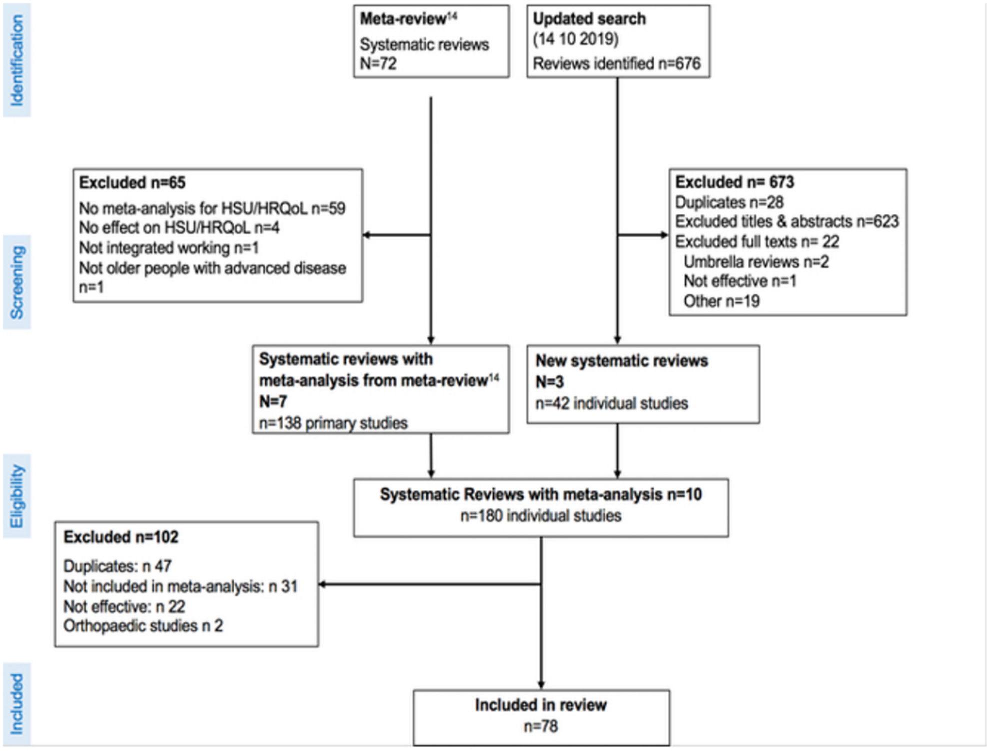

Figure 1. PRISMA flowchart for selection of primary studies

89x89mm (300 x 300 DPI)

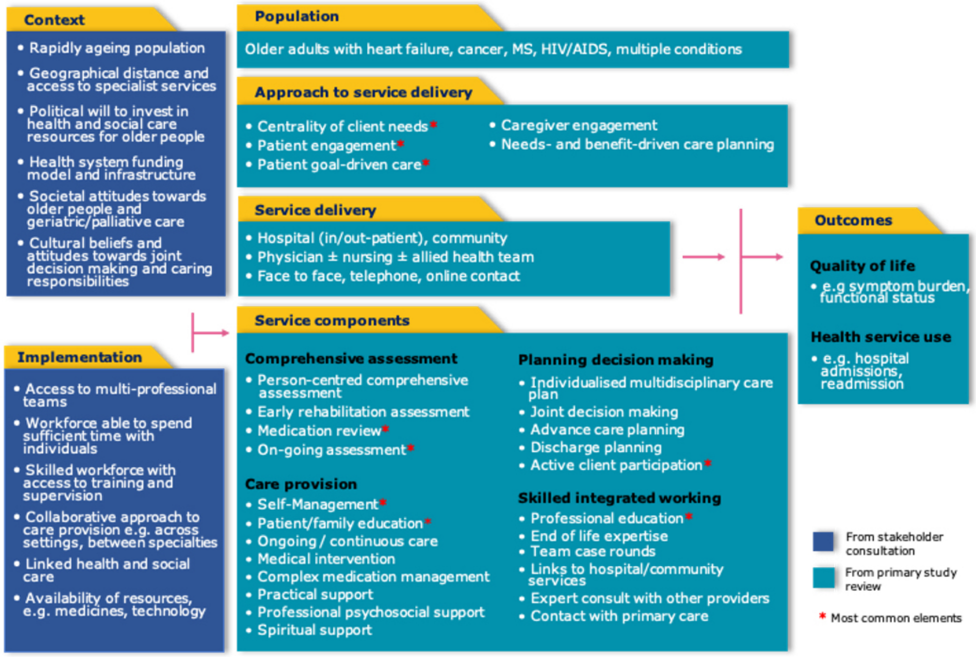

Figure 2. Common components logic model detailing effective service delivery models for older people with advanced progressive conditions

89x89mm (300 x 300 DPI)

Supplementary material 1. Eligibility criteria for WHO 'Rapid review of service delivery models for older people at the end of life to maximise quality of life.'

	**Inclusion**		**Exclusion**
A	Participants at the end of life or living with advanced disease	Where information is available patients described as being in the last 1-2 years of life, or with advanced disease defined as advanced or metastatic cancer; chronic respiratory disease GOLD stage III-IV / grade C-D; heart failure New York Heart Association stage III or IV; progressive neurological disease; and frailty (excluding pre-frail)	Participants not described as being at the end of life or do not have advanced disease
B	Participants are older people	Where information is available at least 50% of the population must be greater than 60 years old or mean age greater than 60 years old	Where the information is available less than 50% of participants are older than 60 years old or mean age greater than 60 years old
C	Intervention must be a service delivery model aiming to improve quality of life	Service model must be an overarching model of health care provision with multiple components and interacting elements	Intervention is a single component intervention or focussing on post death intervention.
D	Outcome must be focussed on quality of life, function and dignity or cost-effectiveness	Outcomes of quality of life, function and dignity to include wellbeing, resilience, personal satisfaction, empowerment, goal attainment, autonomy, independence, mastery, adaptation, symptoms including pain, breathlessness, anxiety, depression, constipation, falls, any measure of psychosocial or spiritual distress, patient and caregiver satisfaction Outcome of cost effectiveness	Outcome not focussed on quality of life, function or dignity
E	Design must be a review	Review must have searched at least 2 sources, one of which must be an electronic database	Non-review level paper e.g., primary intervention
F	Review may include controlled or non-controlled trials	Review can include trials that are randomised (cluster, parallel, single-stage or cross-over design), non-randomised trials, controlled before-after studies, interrupted time series studies and repeated measures studies. Control group can include usual care, attention control, active control or no control	Review focussing on opinion piece, case studies, case series or descriptive studies

For peer review only

Supplementary material 2. Search Strategy for Medline

The search strategy was adapted for searches on The Cochrane Database of Systematic Reviews, CINAHL and Embase databases [14] and included studies published between January 2000 and October 2017.

	Population EoL /advanced disease	Intervention e.g. hospital	Outcome
MESH terms	Exp Terminally ill /<br>Exp Terminal care/<br>Palliative Care/<br>Frailty/	Exp Patient admission/<br>Exp Patient readmission/<br>Geriatric nursing/<br>Primary nursing/<br>Hospice and palliative care nursing/<br>Exp Nursing services/<br>Symptom Assessment/<br>Geriatric Assessment/<br>Needs assessment/<br>Hospital volunteers/<br>Nursing process/<br>Exp Patient care planning/<br>Exp Progressive patient care/<br>Exp Caregivers/<br>Exp Home care services/<br>Exp Hospice care/<br>Exp Patient Care Team<br>Exp Continuity of Patient Care/	Exp Quality of life/<br>Exp Pain/<br>Exp Pain management/<br>Exp Dyspnea/<br>Exp Anxiety/<br>Exp Anxiety disorders/<br>Depression/<br>Exp Depressive disorder/<br>Personal satisfaction/<br>Exp Activities of daily living/<br>Constipation/<br>Accidental Falls/<br>Exp Mental health/<br>Exp Social isolation/<br>Exp Social support/<br>Exp Patient satisfaction/<br>Exp Budgets/<br>Exp Costs and cost analysis/<br>Economics/<br>Exp Economics, hospital/<br>Exp Economics, medical/<br>Economics, nursing/<br>Exp Fees and charges/<br>Exp Resource allocation/<br>Value of life/
Key terms	EoL.tw<br>End?of?life.tw<br>Dying.tw<br>Palliative.tw<br>Last adj4 life.tw<br>Hospice.tw<br>Life limit*tw<br>Advanced disease*tw<br>Palliative treatment.tw<br>Palliative medicine.tw<br>Terminal care.tw<br>Terminally ill.tw<br>End-of-life care.tw<br>Hospice care.tw<br>Palliation.tw.<br>Palliative care$.tw.<br>Multi*morbidity.tw<br>Co*morbidity.tw<br>((Frail old*) AND (people OR adult* OR person*)).ti,ab<br>Frail*.tw<br>Frail elder*.ti,ab<br>Frailty syndrome*.ti,ab<br>Advanced illness.tw	Integrated care.tw<br>Model adj4 care.tw<br>Multi?disciplin*tw<br>Multi?disciplinary team.tw<br>Volunteer* tw<br>Volunt*tw<br>Hospital adj3 home.tw<br>Comprehensive assess*tw<br>Holistic assess*<br>(special$ adj2 palliat$).tw.<br>Nurse-led.tw<br>Co?ordination adj3 care.tw<br>Care plans.tw<br>Care?giver*.tw<br>Person?centr*.tw<br>Self?manage*.tw<br>Community health worker*.tw<br>Service delivery.tw<br>Community?based.tw<br>Home visit*.tw<br>Case management.tw<br>Care management.tw	Good death.tw<br>Symptom*.tw<br>Concern*.tw<br>Attainment<br>Dignity.tw<br>Empowerment.tw<br>Transition*.tw<br>Pain.tw<br>Dyspn?ea.tw<br>Breathless*.tw<br>Anxiety.tw<br>Anxious.tw<br>Depress*.tw<br>Quality of life.tw<br>Qol.tw<br>(quality adj2 life).tw.<br>Distress.tw<br>Wellbeing.tw<br>ADL*.tw<br>Activities of daily living.tw<br>Constipat*.tw<br>Fall*.tw<br>Mobil*.tw<br>Symptom management.tw.<br>Psychosocial.tw.<br>(psycho adj social).tw.<br>Psychological distress.tw.<br>Enablement.tw<br>Mastery.tw<br>Resilience.tw<br>Stress.tw<br>Financ*tw

For peer review only

			(Cost* or economic*).ti<br>(Cost* adj2 (effective* or utilit* or benefit* or minimi*)).ab.<br>Economic model*.tw<br>(Budget* or fee* or financ* or pricing or price* or resource* allocat* or (value adj2 (monetary or money))).ti,ab
BOLEAN TERMS	OR	OR	OR
	AND		
LIMIT	((Overview*.ti OR Review.ti OR Synthesis.ti OR Summary.ti OR Cochrane.ti OR Analysis.ti) AND (reviews.ti OR meta-analyses.ti OR articles.ti OR umbrella.ti)) OR ''umbrella review''.ti,ab OR (meta-review.ti.ab ORMetareview.ti,ab) OR ((overview*.ti OR Reviews.ti) AND (systematic.ti OR Cochrane.ti)) OR (reviews.ti,ab and (meta.ti,ab OR Published.ti,ab OR Quality.ti,ab OR Included.ti,ab OR summar*.ti,ab)) OR (''cochrane reviews''.ti,ab) OR (evidence.ti AND (reviews.ti OR meta-analyses.ti))		

For peer review only

Supplementary material 3. Data extraction framework: CATWOE elements

Service Delivery Model area (CATWOE)	Model elements / processes	Operational definition
**C(customers):** Target population and case mix	Population needs assessment	Population targeted by the intervention
	Setting	Where intervention is delivered: *Hospital in-patients/ hospital out-patients/ home/ primary care/community / mixed settings*
**A(actors):** Workforce including professions, level of skill and training	Multi-disciplinary team care	Multi-disciplinery team comprises ≥3 disciplines
	Rehabilitation expertise or training	Recognised rehabilitation expertise or training (i.e. Allied Health Professionals)
	End of life expertise or training	Recognised Palliative Care expertise or training (i.e. Palliative Care physician/or specialist Palliative Clinical Nurse Specialist or explicit statement of palliative and end of life care training)
	Professional education	Persons delivering intervention are educated and trained to nationally recognised standards and regulations.
**T(transformation process):** Service model elements / components	Comprehensive Assessment	I.e. comprehensive assessment- across multiple domains including physical/psychological/social/spiritual
	Case Management	Each person's overall care assigned to a team or individual
	Collaborative Working	Working across disciplines to plan services and deliver care to meet needs
	Route(s) of access, source and criteria for referral	How are participants recruited or eligible to participate?
	Professional psychosocial support	Explicit psychological support offered as component of intervention (i.e. psychologist/counsellor/Social Worker)
	Contact established with primary care or attending physician	Does interventionist contact physician as part of intervention?
	Patient and family education	Education for patient &/or family caregiver
	Individual multi-disciplinary care plan	Explicit description of multi-disciplinary team care plan
	Medical intervention	Medical intervention part of intervention, not alongside
	Team case rounds	Intervention includes team meetings, not usual care meetings
	Practical support	Any practical help i.e. in home, with medication boxes, equipment

For peer review only

	Early rehabilitation assessment	Intervention includes rehabilitation early in course of persons integrated geriatric care or integrated palliative care
	Systematic risk screening	Risk screening part of intervention delivery
	Discharge planning	Discharge planning a component of intervention
	Bereavement support	As stated
	Spiritual support	As stated
	Advance care planning	Formal advanced care planning
	Emergency response plan	Emergency only or plan for acute changes, i.e. worsening symptoms
	Self-management	As stated
	Medication review	Review part of intervention
	Complexity/medication management	Ongoing management of medication during intervention
**T:** Mode of delivery	Physician home visits	As part of intervention
	Physician available around the clock	As stated
	Interaction between professional and patient	Face to face/telephone/online or combination
	Access to dedicated inpatient beds	As stated
	Around the clock home visits available	As stated
	Ongoing assessment	Intervention includes multiple points of or ongoing assessment
**T:** Operational tools & guidance to support practice, e.g. assessment or decision support tools	Chart in the home	Diary, manual, medical/nursing record
	Medical review: standardized admission assessment	Explicitly reports standardised assessment is used
	Patient-centred care: standardized comprehensive assessment	Evidence of use comprehensive assessment tools or guidance relating to patient needs
**W (worldview):** Methods of integrated working	Joint provision across health and social care	Care involves explicit links between health and social care (in residential/nursing home care or home) providers
	Linkage with hospital	Intervention involves links with hospital services or is provided by hospital
	Linkage between community services	Intervention involves links with community services
	Expert consultation with other providers	Intervention involves consultation with other multi-disciplinary teams.
	Linkage with residential hospice	As stated
	One contact number	As stated- but reports contact number given

For peer review only

	Ongoing / continuous care	Ongoing care following the intervention made explicit
**W**: Conceptual model	Patient directed goal driven care	Patient involved in setting goals
	Centrality of patient* needs	Intervention focuses on individual patients needs
	Care mandate -service driven or needs- and benefits-driven	Service driven intervention = same intervention delivered to everyone with customisation and tailoring<br>Needs driven = patients' needs determine delivery of individualised intervention components
	Joint decision-making	Patient involved in decision making during delivery of intervention
	Active patient participation	Involves client or patient actively participating in behaviours
	Patient engagement	Intervention targets patient
	Caregiver engagement	Intervention targets caregiver
**W:** Provider Sector(s)	Visiting volunteer sectors	Volunteers explicitly involved in delivery of intervention
**O (Owners)**	Location	Country name
	World Bank status	High, Upper middle, Low Middle, Low
	Health service funding	State, private for profit, private non-profit, voluntary sector, other
**E (environmental constraints):** Country setting, sites, human resources,	Enabling environment	Policy, infrastructure, workforce training, rural or urban settings
	Resource requirements -human resources	Human resources- name all professionals involved in intervention delivery

*for consistency, we decided to use term 'patient' while acknowledging that in some settings the term client may be interchangeable or preferred.

For peer review only

Supplementary material 4. Mapping CATWOE domains to Logic Model template domains

CATWOE Domains →	Logic Model Domains
**C** (Customers)	**Population**<br>Target population and case mix
**A** (Actors)	**Service delivery**<br>Workforce including professions, level of skill and training
**T** (Transformation processes)	**Service delivery**<br>Mode of delivery<br>**Service components**<br>Service model elements/components<br>Operational tools to support practice
**W** (Worldview)	**Service components**<br>Methods of integrated working<br>**Approach to service delivery**<br>Conceptual model
**E** (Environmental constraints)	**Context**<br>Setting, sites, size of population served, infrastructure<br>**Implementation**<br>Policy, workforce, training<br>resource requirements

Supplementary material 5. Included study characteristics

Author / year	WHO Region	Country	WBC Income status	Population	Setting	Sample Size	QoL Outcome Measure	HSU
**Integrated Geriatric Care**								
Applegate 1990[1]	Americas	USA	High	Acutely ill older people	Hospital in-pts	156	Basic self-care activities	n/a
Asplund 2000[2]	Europe	Sweden	High	Acutely ill older people	Hospital in-pts	190	n/a	Shorter hospital length of stay and reduced hospital readmissions
Austin 2005[3]	Europe	UK	High	People with heart failure	Hospital out-pts	200	Functional performance (6MWT), perceived exertion (Borg RPE), Minnesota living with heart failure	n/a
Barnes 2012[4]	Americas	USA	High	Acutely ill older people	Hospital in-pts	1632	n/a	Shorter hospital length of stay and reduced hospital readmissions
Blue 2001[5]	Europe	UK	High	People with heart failure	Home	165	n/a	All cause and Heart Failure hospital readmission rates
Burton 2013[6]	W. Pacific	Australia	High	Older people	Home	80	Physical activity tests (i.e. sit to stand), Late life Disability Instrument, Late life function Instrument.	n/a
Capomollo 2002[7]	Europe	Italy	High	People with heart failure	Hospital out-pts	234	n/a	All cause hospital readmission rates
Chang 2005 [8]	Americas	USA	High	People with heart failure	Hospital out-pts	95	Minnosota Living with Heart Failure (MLwHF) and peace subscale of the spiritual quality of life.	n/a
Clark 2013[9]	Americas	USA	High	People with advanced cancer	Home	129	Functional Assessment of Cancer Therapy-General (FACT-G) scale	n/a
Clemson 2004[10]	W. Pacific	Australia	High	Older people	Community	310	Number of falls; falls & mobility efficacy scales; Physical Activity Scale for the	n/a

For peer review only

							Elderly; worry scale; SF36 (physical components and mental components)	
Clemson 2012[11]	W. Pacific	Australia	High	Older people	Home	317	Number of falls, balance and strength, EQ-5D, EQ-VAS, National Health and Nutrition Examination Survey (I), Late life function index, PASE.	n/a
Cline 1998[12]	Europe	Sweden	High	People with heart failure	Mixed secttings (IP, OP)	190	The quality of life in heart failure questionnaire; Nottingham health profile, patient global health assessment	All cause hospital readmission rate
Close 1999[13]	Europe	UK	High	Acutely ill older people	Mixed settings (ER, H)	397	Number of falls, Barthel Index	n/a
Collard 1985[14]	Americas	USA	High	Acutely ill older people	Hospital in-pts	720	Fewer Falls	n/a
Counsell 2007[15]	Americas	USA	High	Older people	Home	951	SR36; Assets & Health Dynamics of the oldest old (AHEAD) survey	Shorter hospital length of stay and reduced hospital readmissions
Covinsky 1997[16]	Americas	USA	High	Acutely ill older people	Hospital in-pts	650	n/a	Shorter hospital length of stay and reduced hospital readmissions
de Lusignan 2001[17]	Europe	UK	High	People with heart failure	Mixed settings (OP, H)	20	General Health Questionnaire and Chronic heart failure symptomology questionnaire	n/a
Doughty 2002[18]	W. Pacific	New Zealand	High	People with heart failure	Mixed settings (IP, H, OP)	197	Minnesota Living with Heart Failure Questionnaire	Hospital Readmission rate for Heart Failure,
Dunbar 2015[19]	Americas	USA	High	People with heart failure	Mixed settings (IP, H, OP)	134	Minnesota Living with Heart Failure Questionnaire, EQ-5D, 6 minute walk test	n/a
Ekman 1998[20]	Europe	Sweden	High	People with heart failure	Hospital out-pts	158	n/a	All cause hospital readmission rate
Fretwell 1990[21]	Americas	USA	High	Acutely ill older people	Mixed settings (IP, OP)	436	n/a	Hospital length of stay

For peer review only

Gary 2010[22]	Americas	USA	High	People with heart failure	Home	74	Minnesota Living with Heart Failure Questionnaire	n/a
Gitlin 2006[23]	Americas	USA	High	Older people	Home	319	Falls Efficacy Scale; three items from Activities-specific Balance Confidence Scale (confident walking up/down stairs, bending picking up slipper from floor., getting in/out of car without falling).	n/a
Goldberg 2003[24]	Americas	USA	High	People with heart failure	Home	282	Medical Outcome Study 12 Item Short Form (SF-12), Medical Outcomes Study Health Distress Scale, Minnesota Living with Heart Failure Questionnaire, and overall Patient Satisfaction (single item) with heart failure care.	n/a
Harrison 2002[25]	Americas	Canada	High	People with heart failure	Mixed settings (IP, H)	192	Minnesota living with Heart Failure Questionnaire, SF 36	Hospital Readmission rate
Jaarsma 1999[26]	Europe	Netherlands	High	People with heart failure	Mixed settings (IP, H)	179	Heart failure Self-Care Behaviour	Hospital Readmission rate
Jerant 2001[27]	Americas	USA	High	People with heart failure	Home	37	n/a	All cause and Heart Failure hospital readmission rate
Kasper 2002[28]	Americas	USA	High	People with heart failure	Mixed settings (IP, OP)	200	Minnesota living with heart failure, Duke activity status index	Readmissions
Krumholz 2002[29]	Americas	USA	High	People with heart failure	Mixed settings	88	n/a	Hospital Readmission rate
Lang 2018[30]	Europe	UK	High	People with heart failure	Home	50	Minnesota Living with Heart Failure Questionnaire; Hospital anxiety and depression scale; EQ-5D	n/a
Laramee 2003[31]	Americas	USA	High	People with heart failure	Mixed settings (IP, H)	287	n/a	Hospital Readmission rate for Heart Failure
Ledwidge 2003[32]	Europe	Ireland	High	People with heart failure	Mixed settings (IP, H)	98	n/a	Heart Failure hospital readmission rates

For peer review only

Luskin 2002[33]	Americas	USA	High	People with heart failure	Hospital out-pts	33	Geriatric Depression Scale, Perceived Stress Scale, Life Orientation Test, State Trait Anxiety Inventory, Medical Outcome Survey Questions 3 and 9, Minnesota Living with Heart Failure, Self-report physical fitness, Six-minute walk,	n/a
Markle-Reid 2010[34]	Americas	Canada	High	Older people	Home	109	Mean number of falls during 6-month Follow-Up	n/a
McVey 1989[35]	Americas	USA	High	Acutely ill older people	Hospital in-pts	178	Measurements of Activities of Daily Living	n/a
Naylor 1994[36]	Americas	USA	High	Acutely ill older people	Mixed settings (IP, H)	276	n/a	Hospital Readmission rate
Naylor 1999[37]	Americas	USA	High	Acutely ill older people	Mixed settings (IP, OP, H)	363	n/a	All cause hospital readmission rate
Northouse 2007[38]	Americas	USA	High	People with cancer	Home	263	Functional Assessment of Cancer Therapy-General (FACT-G) scale	n/a
Pugh 2001[39]	Americas	USA	High	People with heart failure	Mixed settings	58	n/a	Hospital Readmission rate for Heart Failure
Rainville 1999[40]	Americas	USA	High	People with heart failure	Mixed settings (OP, H)	34	n/a	Heart Failure hospital readmission rate
Rich 1995[41]	Americas	USA	High	People with heart failure	Mixed settings (IP, OP)	282	Chronic Heart Failure Questionnaire	All cause hospital readmission rate
Rich 1993[42]	Americas	USA	High	People with heart failure	Mixed setting (IP, OP)	98	Chronic Heart Failure Questionnaire	Readmission during follow-up
Riegel 2002[43]	Americas	USA	High	People with heart failure	Home	358	n/a	All cause and Heart Failure hospital readmission rate
Rubenstein 1984[44]	Americas	USA	High	Acutely ill older people	Hospital in-pts	123	Personal Self-maintenance scale;	n/a
Rubin 1993[45]	Americas	USA	High	Acutely ill older people	Community	200	Katz Activities of daily living Index, Instrumental Activities of Daily Living (Five-Item OARS Scale)	n/a

For peer review only

Saltvedt 2006[46]	Europe	Norway	High	Acutely ill older people	Hospital in-pts	254	Barthel Index	n/a
Serxner 1998[47]	Americas	USA	High	People with heart failure	Hospital out-pts	109	n/a	Hospital Readmission rate
Sherwood 2017[48]	Americas	USA	High	People with heart failure	Community	180	Global score Kansas City Cardiomyopathy Questionnaire (KCCQ); Beck Depression Inventory II; Speilberger State-Trait Anxiety Inventory; Heart Failure Attitudes about Impairment Questionnaire, 6-minute walking test.	Reducing worsening heart failure hospitalisations
Stewart S 1998[49]	W. Pacific	Australia	High	People with heart failure	Mixed settings (IP, H)	97	n/a	All cause and Heart Failure hospital readmission rate
Stewart M 1999 [50]	Americas	USA	High	Acutely ill older people	Hospital in-pts	61	n/a	All cause and Heart Failure hospital readmission rate
Stromberg 2003[51]	Europe	Sweden	High	People with heart failure	Hospital out-pts	106	n/a	Heart Failure hospital readmission rate
Thomas 1993[52]	Americas	USA	High	Acutely ill older people	Hospital in-pts	120	Katz Functional activity rating scale ADL	Hospital length of stay
Trochu 2004[53]	Europe	France	High	People with heart failure	Mixed settings (OP, H)	202	n/a	All cause and Heart Failure hospital readmission rate
Tsuyuki 2004[54]	Americas	Canada	High	People with heart failure	Mixed settings (IP, OP, H)	276	n/a	Hospital Readmission rate for Heart Failure
Varma 1999[55]	Europe	UK	High	People with heart failure	Mixed settings (OP, H)	83	Minnesota living with Heart Failure Questionnaire and the SF-36	n/a
Vidan 2009[56]	Europe	Spain	High	Acutely ill older people	Hospital in-pts	542	Independence in 6 basic Activities of daily living, bathing, dressing, toileting, transferring from bed to chair, continence, and eating.	Length of hospital stay
Wang 2016[57]	SE Asia	Taiwan	High	People with heart failure	Hospital out-pts	92	Piper fatigue Scale (PFS) , Minnesota living with HF	n/a

For peer review only

							questionnaire (MLHFQ) symptom distress, anxiety	
Yu 2010[58]	SE Asia	Hong Kong, China	High	People with heart failure	Hospital out-pts	158	World Health Organization Quality of Life Questionnaire (WHOQOL-BREF-HK)	n/a
Zelada 2009[59]	Americas	Peru	High middle	Acutely ill older people	Hospital in-pts	143	Katz Scale	Length of hospital stay
**Integrated Palliative Care**								
Bakitas 2009[60]	Americas	USA	High	People with advanced cancer	Home	322	Functional Assessment of Chronic Illness Therapy for Palliative Care; Edmonton Symptom Assessment Scale	n/a
Bakitas 2015[61]	Americas	USA	High	People with advanced cancer	Mixed settings (OP, H)	207	Functional Assessment of Chronic Illness Therapy for Palliative Care (FACIT-PAL); FACIT-PAL Treatment Outcome Index; Quality at End of Life (Qual-E), Center for Epidemiological Studies-Depression Scale (CES-D)	n/a
Brannstrom 2014[62]	Europe	Sweden	High	People with heart failure	Mixed settings (OP, H)	72	Edmonton Symptom Assessment System (ESAS), EQ-5D, Kansas City Cardiomyopathy Questionnaire (KCCQ)	n/a
Edmonds 2010[63]	Europe	UK	High	People with multiple sclerosis	Mixed settings (OP, H)	52	Mulitple Sclerosis Impact Scale (MSIS), Palliative Outcome Scale, Modified Lawton Positivity Questionnaire	n/a
Given 2002[64]	Americas	USA	High	People with cancer	Home	113	SF-36	n/a
Higginson 2014[65]	Europe	UK	High	People with advanced diseases	Mixed settings (OP, H)	105	Chronic Respiratory Disease Querstionnaire (mastery), Breathlessness severity, London Chest Actiities of Daily Living Questionnaire, EQ-5D & EQ-VAS, Palliative	n/a

For peer review only - http://bmjopen.bmj.com/site/about/guidelines.xhtml

							outcome scale, Hospital anxiety and depression scale.	
Jordhoy 2001[66]	Europe	Norway	High	People with advanced cancer	Mixed settings (IP, OP, H)	434	European Organisation for Research and Treatment of Cancer Quality of Life Questionnaire C30 (EORTC QLQ-C30)	n/a
Lowther 2015[67]	Africa	Kenya	Low middle	People with HIV	Hospital out-pts	120	African Palliative Care Outcome Scale	n/a
Maltoni 2016[68]	Europe	Italy	High	People with advanced cancer	Hospital out-pts	207	Functional Assessment of Cancer Therapy-Hepato-biliary (FACT-HEP) and FACT-HEP Trial Outcome Index.	n/a
Ozcelik 2014[69]	Europe	Turkey	High middle	People with advanced cancer	Mixed settings (IP, OP, H)	44	Edmonton Symptom Assessment Scale (ESAS) and European Organisation for Research and Treatment of Cancer Quality of Life Questionnaire C30 (EORTC QLQ C30)	n/a
Rogers 2017[70]	Americas	USA	High	People with heart failure	Mixed settings (IP, OP, H)	150	Kansas City Cardiomyopathy questionnaire (KCCQ), Functional assessment of chronic illness therapy palliative care scale (FACIT-Pal) assessed at 6 months. Hospital Anxiety and Depression Scale (HADS), Functional Assessment of Chronic Illness Therapy—Spiritual Well-Being [FACIT-Sp)	n/a
Rummans 2006[71]	Americas	USA	High	People with advanced cancer	Hospital out-pts	115	Spitzer QoL Uniscale and Linear analog scales of assessment	n/a
Sidebottom 2015[72]	Americas	USA	High	People with heart failure	Hospital in-pts	232	Minnesota living with Heart Failure Questionnaire	n/a
Steel 2016[73]	Americas	USA	High	People with advanced cancer	Mixed settings (OP, H)	261	Center for epidemiological studies Depression scale	n/a

For peer review only - http://bmjopen.bmj.com/site/about/guidelines.xhtml

							(CES-D), Brief pain inventory, FACT-Hepatobiliary.	
Tattersall 2014[74]	W. Pacific	Australia	High	People with advanced cancer	Hospital out-pts	120	McGill QoL questionnaire, Rotterdam Symptom Checklist	n/a
Temel 2010[75]	Americas	USA	High	People with advanced cancer	Hospital out-pts	151	Functional Assessment of Cancer Therapy - Lung; Hospital Anxiety and Depression Scale; Patient Health Questionnaire	n/a
Temel 2017[76]	Americas	US	High	People with advanced cancer	Hospital out-pts	350	Functional Assessment of Cancer Therapy-General (FACT-G) scale; Patient Health Questionnaire-9 (PHQ-9); Hospital Anxiety and Depression Scale(HADS)	n/a
Wong 2016[77]	SE Asia	China	High	People with heart failure	Home	84	MQOL-HK, McGill Quality of Life Questionnaire–Hong Kong adaptation	n/a
Zimmermann 2014[78]	Americas	Canada	High	People with advanced cancer	Mixed settings (OP, H)	461	Functional Assessment of Chronic Illness Therapy Spiritual Well-Being [FACIT-Sp); Quality of Life at the End of Life (Qual E); Edmonton Symptom Assessment System (ESAS); satisfaction with care (FAMCARE); Cancer Rehabilitation Evaluation System Medical Interaction Subscale CARES-MIS	n/a

Key: IP =In-patients; OP = out-patients, ER =Emergency Room, H= home; WBC= World Bank Classification n/a = not assessed or not assessed in meta-analysis

For peer review only - http://bmjopen.bmj.com/site/about/guidelines.xhtml

Supplementary material 6. Assessment of Methodological Quality in Included Reviews (AMSTAR)

First Author, Year	A priori design provided	Duplicate study selection/ data extraction	Systematic literature search performed	Status of publication used as an inclusion criterion	List of studies (included and excluded) provided	Characteristics of the included studies provided	Scientific quality of included studies assessed and documented	Scientific quality of included studies used appropriately in formulating conclusions?	Were the methods used to combine the findings of the studies appropriate?	Was the likelihood of publication bias assessed?	Was the conflict of interest included?	Total
**Cui 2019**	No	Yes	Yes	Yes	No	No	Yes	No	Yes	Yes	Yes	**7**
**De Coninck, 2017**	Yes	No	Yes	No	Yes	Yes	Yes	Yes	Yes	No	No	**7**
**Ekdahl 2015**	No	Yes	Yes	No	No	Yes	Yes	Yes	Yes	No	No	**6**
**Fox 2012**	Yes	Yes	Yes	No	Yes	Yes	Yes	Yes	Yes	Yes	Yes	**9**
**Fulton 2019**	Yes	Yes	Yes	No	Yes	Yes	Yes	Yes	Yes	No	Yes	**9**
**Haun 2017**	Yes	Yes	Yes	Yes	Yes	Yes	Yes	Yes	Yes	Yes	No	**9**
**Kavalieratos 2016**	Yes	Yes	Yes	No	No	Yes	Yes	Yes	Yes	Yes	No	**8**
**McAlister 2004**	Yes	Yes	Yes	No	No	No	No	No	Yes	No	No	**4**
**Phillips 2004**	Yes	Yes	Yes	No	No	Yes	Yes	Yes	Yes	Yes	No	**8**
**Kassianos 2018**	Yes	Yes	Yes	Yes	No	Yes	Yes	Yes	Yes	Yes	Yes	**10**
											**Median**	**8**

Supplementary material 7. Risk of Bias Table for included studies

Author/ Year	Randomisation Sequence generation	Allocation Concealment	Blinding of participants and personnel	Blinding of outcome assessments	Incomplete outcome assessment	Selective reporting	Other bias
**Integrated Geriatric Care**							
Applegate 1990	Low	High	High	High	Low	Low	Low
Asplund 2000	Low	Low	High	High	High	Unclear	Low
Austin 2005	Low	Low	Low	Unclear	Low	Low	Unclear
Barnes 2012	Low	High	High	High	Unclear	Low	Low
Blue 2001	Unclear	Unclear	High	Unclear	Unclear	High	Unclear
Burton 2013	Low	Low	High	High	Low	Low	Unclear
Capamello 2002	High	High	High	Unclear	Unclear	High	High
Chang 2005	Low	Unclear	Unclear	Unclear	Low	Low	Unclear
Clark M 2013	High	High	High	High	High	Unclear	Unclear
Clemson 2004	High	Low	High	Low	High	Low	Unclear
Clemson 2012	Low	Low	High	Low	Low	Low	Unclear
Cline 1998	Low	Low	High	Unclear	Low	Low	Unclear
Close 1999	Low	Low	High	High	Low	High	Low
Collard 1985	Low	High	Low	Unclear	High	High	Low
Counsell 2007	Low	Low	Low	Low	Unclear	Unclear	Low
Covinsky 1997	Low	Low	Unclear	Unclear	Low	Low	Low
de Lusigan 2001	Low	Unclear	High	Unclear	Low	Low	Unclear
Doughty 2002	Low	Low	High	Unclear	Low	Low	Unclear
Dunbar 2015	Low	Low	Low	Unclear	Low	Low	Unclear
Ekman 1998	Low	Unclear	High	Unclear	Unclear	High	Unclear
Fretwell 1990	Unclear	Unclear	Unclear	Unclear	Low	Low	Low
Gary 2010	Unclear	Unclear	High	Low	Low	Low	Unclear
Gitlin 2006	Low	Low	High	Low	Low	Low	Low
Goldberg 2003	Low	Low	High	High	Low	Low	Unclear
Harrison 2002	Low	Low	Low	Unclear	Low	Low	Unclear
Jaarsma 1999	Low	Unclear	Unclear	Low	Low	Low	Unclear
Jerant 2001	Low	Unclear	High	High	Low	High	Unclear
Kasper 2002	Low	Unclear	Unclear	Unclear	Low	Low	Unclear
Krumholz 2002	Unclear	Unclear	High	Low	Low	High	Unclear
Lang 2018	Low	Unclear	High	Low	Low	Low	Unclear
Laramee 2003	Unclear	Unclear	High	High	Unclear	High	Unclear
Ledwidge 2003	High	High	High	Unclear	Low	High	Unclear
Luskin 2002	High	Unclear	High	Unclear	Low	Low	Unclear
Markle-Reid 2010	Low	Low	High	Low	Low	Low	Unclear
McVey 1989	Low	Low	High	Low	High	High	Unclear
Naylor 1994	Low	Low	Low	Unclear	Low	High	Unclear
Naylor 1999	Low	Low	Low	Low	Low	High	Unclear
Northouse 2007	Low	Low	High	High	Low	Low	Low
Pugh 2001	High	High	High	High	High	High	High
Rainville 1999	High	High	Unclear	High	Low	High	High
Rich 1995	Low	Low	Low	Unclear	Low	Low	Unclear

Rich 1993	High	High	High	High	Low	High	High
Riegel 2002	High	High	High	Unclear	Unclear	High	High
Rubenstein 1984	Unclear	Unclear	High	Unclear	High	Low	Unclear
Rubin 1993	Low	Low	High	Low	High	Low	Unclear
Saltvedt 2006	Low	Low	High	Low	High	Low	Low
Serxner 1998	Unclear	Unclear	High	High	High	High	High
Sherwood 2017	Low	Low	High	Unclear	Low	Low	Unclear
Stewart S 1998	Unclear	Unclear	High	High	Low	High	High
Stewart M 1999 (Fox)	High	Unclear	Unclear	Unclear	Unclear	Unclear	Low
Stromberg 2003	Low	Low	High	Low	Low	High	Unclear
Thomas 1993	Low	Low	High	High	High	Low	Low
Trochu 2004	Unclear	Unclear	Unclear	Unclear	Unclear	Unclear	Unclear
Tsuyuki 2004	Low	Low	High	Unclear	Low	Unclear	Unclear
Varma 1999	Unclear	Unclear	Unclear	High	High	High	High
Vidan 2009	High	Unclear	Unclear	Unclear	High	Unclear	Low
Wang 2016	Unclear	Unclear	High	Low	High	Unclear	Unclear
Yu 2010	High	High	High	High	High	Unclear	Unclear
Zeleda 2009	High	Unclear	Unclear	Unclear	High	Unclear	Low
**Integrated Palliative Care**							
Bakitas 2009	Low	High	High	Unclear	Low	Low	Low
Bakitas 2015	Low	Unclear	High	Low	Low	Low	High
Brannstrom 2014	Unclear	Low	High	Low	High	High	High
Edmonds 2010	Low	Low	High	Low	Unclear	Low	Unclear
Given 2002	Low	Unclear	High	High	Unclear	High	Low
Higginson 2014	Low	Low	High	High	Low	Low	Low
Jordhoy 2001	Unclear	Unclear	High	High	Low	Low	Low
Lowther 2015	Low	Low	High	High	Low	Low	Low
Maltoni 2016	Low	Low	High	Unclear	Low	Low	Low
Ozcelik 2014	High	High	High	High	Low	High	Unclear
Rogers 2017	Low	Unclear	High	High	Low	Low	Unclear
Rummans 2006	Low	Low	High	Low	Low	Low	Low
Sidebottom 2015	Unclear	Unclear	High	High	Low	Low	Low
Steel 2016	Low	Low	High	High	Low	High	High
Tattersall 2014	Low	Low	High	Unclear	High	Unclear	Unclear
Temel 2010	Low	High	High	Unclear	Low	Low	Low
Temel 2017	Low	Low	High	Low	Low	Unclear	Unclear
Wong 2016	Low	Low	Unclear	High	Low	High	Low
Zimmerman 2014	Low	High	Low	High	Low	Low	Low

References

1. Applegate, W.B., et al., *A randomized, controlled trial of a geriatric assessment unit in a community rehabilitation hospital.* New England Journal of Medicine, 1990. **322**(22): p. 1572-1578.
2. Asplund, K., et al., *Geriatric-based versus general wards for older acute medical patients: a randomized comparison of outcomes and use of resources.* Journal of the American Geriatrics Society, 2000. **48**(11): p. 1381-1388.
3. Austin, J., et al., *Randomised controlled trial of cardiac rehabilitation in elderly patients with heart failure.* European Journal of Heart Failure, 2005. **7**(3): p. 411-417.

For peer review only - http://bmjopen.bmj.com/site/about/guidelines.xhtml

4. Barnes, D.E., et al., *Acute care for elders units produced shorter hospital stays at lower cost while maintaining patients' functional status.* Health Affairs, 2012. **31**(6): p. 1227-1236.
5. Blue, L., et al., *Randomised controlled trial of specialist nurse intervention in heart failure.* BMJ, 2001. **323**(7315): p. 715-718.
6. Burton, E., et al., *Effectiveness of a lifestyle exercise program for older people receiving a restorative home care service: a pragmatic randomized controlled trial.* Clinical interventions in aging, 2013. **8**: p. 1591.
7. Capomolla, S., et al., *Cost/utility ratio in chronic heart failure: comparison between heart failure management program delivered by day-hospital and usual care.* Journal of the American College of Cardiology, 2002. **40**(7): p. 1259-1266.
8. Chang, B.-H., et al., *A relaxation response randomized trial on patients with chronic heart failure.* Journal of Cardiopulmonary Rehabilitation and Prevention, 2005. **25**(3): p. 149-157.
9. Clark, M.M., et al., *Randomized controlled trial of maintaining quality of life during radiotherapy for advanced cancer.* Cancer, 2013. **119**(4): p. 880-887.
10. Clemson, L., et al., *The effectiveness of a community-based program for reducing the incidence of falls in the elderly: A randomized trial.* Journal of the American Geriatrics Society, 2004. **52**(9): p. 1487-1494.
11. Clemson, L., et al., *Integration of balance and strength training into daily life activity to reduce rate of falls in older people (the LiFE study): randomised parallel trial.* Bmj, 2012. **345**: p. e4547.
12. Cline, C., et al., *Cost effective management programme for heart failure reduces hospitalisation.* Heart, 1998. **80**(5): p. 442-446.
13. Close, J., et al., *Prevention of falls in the elderly trial (PROFET): a randomised controlled trial.* The Lancet, 1999. **353**(9147): p. 93-97.
14. Collard, A.F., S.S. Bachman, and D.F. Beatrice, *Acute care delivery for the geriatric patient: an innovative approach.* QRB. Quality review bulletin, 1985. **11**(6): p. 180-185.
15. Counsell, S.R., et al., *Geriatric care management for low-income seniors: a randomized controlled trial.* Jama, 2007. **298**(22): p. 2623-2633.
16. Covinsky, K.E., et al., *Do acute care for elders units increase hospital costs? A cost analysis using the hospital perspective.* Journal of the American Geriatrics Society, 1997. **45**(6): p. 729-734.
17. de Lusignan, S., et al., *Compliance and effectiveness of 1 year's home telemonitoring. The report of a pilot study of patients with chronic heart failure.* European journal of heart failure, 2001. **3**(6): p. 723-730.
18. Doughty, R.N., et al., *Randomized, controlled trial of integrated heart failure management. The Auckland Heart Failure Management Study.* European Heart Journal, 2002. **23**(2): p. 139-146.
19. Dunbar, S.B., et al., *Randomized clinical trial of an integrated self-care intervention for persons with heart failure and diabetes: quality of life and physical functioning outcomes.* Journal of cardiac failure, 2015. **21**(9): p. 719-729.
20. Ekman, I., et al., *Feasibility of a nurse-monitored, outpatient-care programme for elderly patients with moderate-to-severe, chronic heart failure.* European Heart Journal, 1998. **19**(8): p. 1254-1260.
21. Fretwell, M.D., et al., *The senior care study: A controlled trial of a consultative/unit-based geriatric assessment program in acute care.* Journal of the American Geriatrics Society, 1990. **38**(10): p. 1073-1081.
22. Gary, R.A., et al., *Combined exercise and cognitive behavioral therapy improves outcomes in patients with heart failure.* Journal of psychosomatic research, 2010. **69**(2): p. 119-131.

For peer review only - http://bmjopen.bmj.com/site/about/guidelines.xhtml

23. Gitlin, L.N., et al., *A randomized trial of a multicomponent home intervention to reduce functional difficulties in older adults.* Journal of the American Geriatrics Society, 2006. **54**(5): p. 809-816.
24. Goldberg, L.R., et al., *Randomized trial of a daily electronic home monitoring system in patients with advanced heart failure: the Weight Monitoring in Heart Failure (WHARF) trial.* American heart journal, 2003. **146**(4): p. 705-712.
25. Harrison, M.B., et al., *Quality of life of individuals with heart failure: a randomized trial of the effectiveness of two models of hospital-to-home transition.* Medical care, 2002: p. 271-282.
26. Jaarsma, T., et al., *Effects of education and support on self-care and resource utilization in patients with heart failure.* European heart journal, 1999. **20**(9): p. 673-682.
27. Jerant, A.F., R. Azari, and T.S. Nesbitt, *Reducing the Cost of Frequent Hospital Admissions for Congestive Heart Failure: A Randomized Trial of a Home Telecare Intervention.* Medical Care, 2001. **39**(11): p. 1234-1245.
28. Kasper, E.K., et al., *A randomized trial of the efficacy of multidisciplinary care in heart failure outpatients at high risk of hospital readmission.* Journal of the American College of Cardiology, 2002. **39**(3): p. 471-480.
29. Krumholz, H.M., et al., *Randomized trial of an education and support intervention to preventreadmission of patients with heart failure.* Journal of the American College of Cardiology, 2002. **39**(1): p. 83-89.
30. Lang, C.C., et al., *A randomised controlled trial of a facilitated home-based rehabilitation intervention in patients with heart failure with preserved ejection fraction and their caregivers: the REACH-HFpEF Pilot Study.* BMJ open, 2018. **8**(4): p. e019649.
31. Laramee, A.S., et al., *Case Management in a Heterogeneous Congestive Heart Failure Population: A Randomized Controlled Trial.* JAMA Internal Medicine, 2003. **163**(7): p. 809-817.
32. Ledwidge, M., et al., *Is multidisciplinary care of heart failure cost-beneficial when combined with optimal medical care?* European Journal of Heart Failure, 2003. **5**(3): p. 381-389.
33. Luskin, F., et al., *A controlled pilot study of stress management training of elderly patients with congestive heart failure.* Preventive cardiology, 2002. **5**(4): p. 168-174.
34. Markle-Reid, M., et al., *The effects and costs of a multifactorial and interdisciplinary team approach to falls prevention for older home care clients 'at risk'for falling: a randomized controlled trial.* Canadian Journal on Aging/La Revue canadienne du vieillissement, 2010. **29**(1): p. 139-161.
35. McVey, L.J., et al., *Effect of a geriatric consultation team on functional status of elderly hospitalized patients: a randomized, controlled clinical trial.* Annals of internal medicine, 1989. **110**(1): p. 79-84.
36. Naylor, M., et al., *Comprehensive discharge planning for the hospitalized elderly: a randomized clinical trial.* Annals of internal Medicine, 1994. **120**(12): p. 999-1006.
37. Naylor, M.D., et al., *Comprehensive Discharge Planning and Home Follow-up of Hospitalized EldersA Randomized Clinical Trial.* JAMA, 1999. **281**(7): p. 613-620.
38. Northouse, L.L., et al., *Randomized clinical trial of a family intervention for prostate cancer patients and their spouses.* Cancer: Interdisciplinary International Journal of the American Cancer Society, 2007. **110**(12): p. 2809-2818.
39. Pugh, L.C., et al., *Case management for elderly persons with heart failure: the quality of life and cost outcomes.* MedSurg Nursing, 2001. **10**(2): p. 71.
40. Rainville, E.C., *Impact of pharmacist interventions on hospital readmissions for heart failure.* American Journal of Health-System Pharmacy, 1999. **56**(13): p. 1339-1342.

For peer review only

41. Rich, M.W., et al., *A Multidisciplinary Intervention to Prevent the Readmission of Elderly Patients with Congestive Heart Failure.* New England Journal of Medicine, 1995. **333**(18): p. 1190-1195.
42. Rich, M.W., et al., *Prevention of readmission in elderly patients with congestive heart failure.* Journal of General Internal Medicine, 1993. **8**(11): p. 585-590.
43. Riegel, B., et al., *Effect of a Standardized Nurse Case-Management Telephone Intervention on Resource Use in Patients With Chronic Heart Failure.* JAMA Internal Medicine, 2002. **162**(6): p. 705-712.
44. Rubenstein, L.Z., et al., *Effectiveness of a geriatric evaluation unit: a randomized clinical trial.* New England Journal of Medicine, 1984. **311**(26): p. 1664-1670.
45. Rubin, C.D., et al., *A randomized, controlled trial of outpatient geriatric evaluation and management in a large public hospital.* Journal of the American Geriatrics Society, 1993. **41**(10): p. 1023-1028.
46. Saltvedt, I., et al., *Randomised trial of in-hospital geriatric intervention: impact on function and morale.* Gerontology, 2006. **52**(4): p. 223-230.
47. Serxner, S., M. Miyaji, and J. Jeffords, *Congestive heart failure disease management study: a patient education intervention.* Congestive Heart Failure, 1998. **4**: p. 23-28.
48. Sherwood, A., et al., *Effects of coping skills training on quality of life, disease biomarkers, and clinical outcomes in patients with heart failure: a randomized clinical trial.* Circulation: Heart Failure, 2017. **10**(1): p. e003410.
49. Stewart, S., S. Pearson, and J.D. Horowitz, *Effects of a Home-Based Intervention Among Patients With Congestive Heart Failure Discharged From Acute Hospital Care.* JAMA Internal Medicine, 1998. **158**(10): p. 1067-1072.
50. Stewart, M., et al., *The impact of a geriatrics evaluation and management unit compared to standard care in a community teaching hospital.* Maryland medical journal (Baltimore, Md.: 1985), 1999. **48**(2): p. 62-67.
51. Strömberg, A., et al., *Nurse-led heart failure clinics improve survival and self-care behaviour in patients with heart failure: Results from a prospective, randomised trial.* European Heart Journal, 2003. **24**(11): p. 1014-1023.
52. Thomas, D.R., R. Brahan, and B.P. Haywood, *Inpatient community-based geriatric assessment reduces subsequent mortality.* Journal of the American Geriatrics Society, 1993. **41**(2): p. 101-104.
53. Trochu, J., S. Baleynaud, and G. Mialet, *Efficacy of a multidisciplinary management of chronic heart failure patients: one year results of a multicentre randomized trial in French medical practice.* Eur Heart J, 2004.
54. Tsuyuki, R.T., et al., *A multicenter disease management program for hospitalized patients with heart failure.* Journal of Cardiac Failure, 2004. **10**(6): p. 473-80.
55. Varma, S., et al., *Pharmaceutical care of patients with congestive heart failure: interventions and outcomes.* Pharmacotherapy: The Journal of Human Pharmacology and Drug Therapy, 1999. **19**(7): p. 860-869.
56. Vidán, M.T., et al., *An intervention integrated into daily clinical practice reduces the incidence of delirium during hospitalization in elderly patients.* Journal of the American Geriatrics Society, 2009. **57**(11): p. 2029-2036.
57. Wang, T.-C., et al., *Effects of a supportive educational nursing care programme on fatigue and quality of life in patients with heart failure: a randomised controlled trial.* European Journal of Cardiovascular Nursing, 2016. **15**(2): p. 157-167.
58. Yu, D.S., D.T. Lee, and J. Woo, *Improving health-related quality of life of patients with chronic heart failure: effects of relaxation therapy.* Journal of advanced nursing, 2010. **66**(2): p. 392-403.
59. Zelada, M.A., R. Salinas, and J.J. Baztán, *Reduction of functional deterioration during hospitalization in an acute geriatric unit.* Archives of gerontology and geriatrics, 2009. **48**(1): p. 35-39.

For peer review only - http://bmjopen.bmj.com/site/about/guidelines.xhtml

60. Bakitas, M., et al., *Effects of a palliative care intervention on clinical outcomes in patients with advanced cancer: the Project ENABLE II randomized controlled trial.* Jama, 2009. **302**(7): p. 741-749.
61. Bakitas, M.A., et al., *Early versus delayed initiation of concurrent palliative oncology care: patient outcomes in the ENABLE III randomized controlled trial.* Journal of Clinical Oncology, 2015. **33**(13): p. 1438.
62. Brännström, M. and K. Boman, *Effects of person-centred and integrated chronic heart failure and palliative home care. PREFER: a randomized controlled study.* European journal of heart failure, 2014. **16**(10): p. 1142-1151.
63. Edmonds, P., et al., *Palliative care for people severely affected by multiple sclerosis: evaluation of a novel palliative care service.* Multiple Sclerosis Journal, 2010. **16**(5): p. 627-636.
64. Given, B., et al. *Pain and fatigue management: results of a nursing randomized clinical trial*. in *Oncology nursing forum*. 2002.
65. Higginson, I.J., et al., *An integrated palliative and respiratory care service for patients with advanced disease and refractory breathlessness: a randomised controlled trial.* Lancet Respiratory Medicine, 2014. **2**(12): p. 979-987.
66. Jordhøy, M.S., et al., *Quality of life in palliative cancer care: results from a cluster randomized trial.* Journal of Clinical Oncology, 2001. **19**(18): p. 3884-3894.
67. Lowther, K., et al., *Nurse-led palliative care for HIV-positive patients taking antiretroviral therapy in Kenya: a randomised controlled trial.* The lancet HIV, 2015. **2**(8): p. e328-e334.
68. Maltoni, M., et al., *Systematic versus on-demand early palliative care: results from a multicentre, randomised clinical trial.* European Journal of Cancer, 2016. **65**: p. 61-68.
69. Ozcelik, H., et al., *Examining the effect of the case management model on patient results in the palliative care of patients with cancer.* American Journal of Hospice and Palliative Medicine®, 2014. **31**(6): p. 655-664.
70. Rogers, J.G., et al., *Palliative care in heart failure: the PAL-HF randomized, controlled clinical trial.* Journal of the American College of Cardiology, 2017. **70**(3): p. 331-341.
71. Rummans, T.A., et al., *Impacting quality of life for patients with advanced cancer with a structured multidisciplinary intervention: a randomized controlled trial.* Journal of Clinical Oncology, 2006. **24**(4): p. 635-642.
72. Sidebottom, A.C., et al., *Inpatient palliative care for patients with acute heart failure: outcomes from a randomized trial.* Journal of palliative medicine, 2015. **18**(2): p. 134-142.
73. Steel, J.L., et al., *W eb-based collaborative care intervention to manage cancer-related symptoms in the palliative care setting.* Cancer, 2016. **122**(8): p. 1270-1282.
74. Tattersall, M., et al., *Early contact with palliative care services: A randomised trial in patients with newly detected incurable metastatic cancer.* 2014.
75. Temel, J.S., et al., *Early palliative care for patients with metastatic non–small-cell lung cancer.* New England Journal of Medicine, 2010. **363**(8): p. 733-742.
76. Temel, J.S., et al., *Effects of early integrated palliative care in patients with lung and GI cancer: a randomized clinical trial.* Journal of Clinical Oncology, 2017. **35**(8): p. 834.
77. Wong, F.K.Y., et al., *Effects of a transitional palliative care model on patients with end-stage heart failure: a randomised controlled trial.* Heart, 2016. **102**(14): p. 1100-1108.
78. Zimmermann, C., et al., *Early palliative care for patients with advanced cancer: a cluster-randomised controlled trial.* The Lancet, 2014. **383**(9930): p. 1721-1730.

For peer review only

PRISMA 2009 Checklist

Section/topic	#	Checklist item	Reported on page #
**TITLE**			
Title	1	Identify the report as a systematic review, meta-analysis, or both.	1
**ABSTRACT**			
Structured summary	2	Provide a structured summary including, as applicable: background; objectives; data sources; study eligibility criteria, participants, and interventions; study appraisal and synthesis methods; results; limitations; conclusions and implications of key findings; systematic review registration number.	2
**INTRODUCTION**			
Rationale	3	Describe the rationale for the review in the context of what is already known.	3 & 4
Objectives	4	Provide an explicit statement of questions being addressed with reference to participants, interventions, comparisons, outcomes, and study design (PICOS).	4 & 5
**METHODS**			
Protocol and registration	5	Indicate if a review protocol exists, if and where it can be accessed (e.g., Web address), and, if available, provide registration information including registration number.	5
Eligibility criteria	6	Specify study characteristics (e.g., PICOS, length of follow-up) and report characteristics (e.g., years considered, language, publication status) used as criteria for eligibility, giving rationale.	5
Information sources	7	Describe all information sources (e.g., databases with dates of coverage, contact with study authors to identify additional studies) in the search and date last searched.	5<br>Supplementary material 1 & 2
Search	8	Present full electronic search strategy for at least one database, including any limits used, such that it could be repeated.	Supplementary material 2
Study selection	9	State the process for selecting studies (i.e., screening, eligibility, included in systematic review, and, if applicable, included in the meta-analysis).	5
Data collection process	10	Describe method of data extraction from reports (e.g., piloted forms, independently, in duplicate) and any processes for obtaining and confirming data from investigators.	5 & 6
Data items	11	List and define all variables for which data were sought (e.g., PICOS, funding sources) and any assumptions and simplifications made.	5 & 6<br>Supplementary material 3
Risk of bias in individual studies	12	Describe methods used for assessing risk of bias of individual studies (including specification of whether this was done at the study or outcome level), and how this information is to be used in any data synthesis.	6<br>Supplementary

For peer review only - http://bmjopen.bmj.com/site/about/guidelines.xhtml

PRISMA 2009 Checklist

			material 5 & 6
Summary measures	13	State the principal summary measures (e.g., risk ratio, difference in means).	n/a
Synthesis of results	14	Describe the methods of handling data and combining results of studies, if done, including measures of consistency (e.g., I^2) for each meta-analysis.	n/a

Section/topic	#	Checklist item	Reported on page #
Risk of bias across studies	15	Specify any assessment of risk of bias that may affect the cumulative evidence (e.g., publication bias, selective reporting within studies).	7
Additional analyses	16	Describe methods of additional analyses (e.g., sensitivity or subgroup analyses, meta-regression), if done, indicating which were pre-specified.	n/a
**RESULTS**			
Study selection	17	Give numbers of studies screened, assessed for eligibility, and included in the review, with reasons for exclusions at each stage, ideally with a flow diagram.	7<br>Figure 1
Study characteristics	18	For each study, present characteristics for which data were extracted (e.g., study size, PICOS, follow-up period) and provide the citations.	7<br>Table 1<br>Supplementary Material 4
Risk of bias within studies	19	Present data on risk of bias of each study and, if available, any outcome level assessment (see item 12).	8<br>Supplementary material 6
Results of individual studies	20	For all outcomes considered (benefits or harms), present, for each study: (a) simple summary data for each intervention group (b) effect estimates and confidence intervals, ideally with a forest plot.	n/a
Synthesis of results	21	Present results of each meta-analysis done, including confidence intervals and measures of consistency.	n/a
Risk of bias across studies	22	Present results of any assessment of risk of bias across studies (see Item 15).	8
Additional analysis	23	Give results of additional analyses, if done (e.g., sensitivity or subgroup analyses, meta-regression [see Item 16]).	n/a
**DISCUSSION**			
Summary of evidence	24	Summarize the main findings including the strength of evidence for each main outcome; consider their relevance to key groups (e.g., healthcare providers, users, and policy makers).	10

For peer review only

For peer review only - http://bmjopen.bmj.com/site/about/guidelines.xhtml

PRISMA 2009 Checklist

Limitations	25	Discuss limitations at study and outcome level (e.g., risk of bias), and at review-level (e.g., incomplete retrieval of identified research, reporting bias).	12
Conclusions	26	Provide a general interpretation of the results in the context of other evidence, and implications for future research.	13<br>Box 1
**FUNDING**			
Funding	27		14

From: Moher D, Liberati A, Tetzlaff J, Altman DG, The PRISMA Group (2009). Preferred Reporting Items for Systematic Reviews and Meta-Analyses: The PRISMA Statement. PLoS Med 6(6): e1000097. doi:10.1371/journal.pmed1000097

For more information, visit: www.prisma-statement.org.

For peer review only
